# Early Phase of Plasticity-Related Gene Regulation and SRF Dependent Transcription in the Hippocampus

**DOI:** 10.1371/journal.pone.0068078

**Published:** 2013-07-23

**Authors:** Giovanni Iacono, Claudio Altafini, Vincent Torre

**Affiliations:** 1 Department of Functional Analysis, International School for Advanced Studies, Trieste, Italy; 2 IIT Italian Institute of Technology, Genova, Italy; Tel-Aviv University, Israel

## Abstract

Hippocampal organotypic cultures are a highly reliable *in vitro* model for studying neuroplasticity: in this paper, we analyze the early phase of the transcriptional response induced by a 20 µ*M* gabazine treatment (GabT), a *GABA-Ar* antagonist, by using Affymetrix oligonucleotide microarray, *RT-PCR* based time-course and *chromatin-immuno-precipitation*. The transcriptome profiling revealed that the pool of genes up-regulated by GabT, besides being strongly related to the regulation of growth and synaptic transmission, is also endowed with neuro-protective and pro-survival properties. By using *RT-PCR*, we quantified a time-course of the transient expression for 33 of the highest up-regulated genes, with an average sampling rate of 10 minutes and covering the time interval [10∶90] minutes. The cluster analysis of the time-course disclosed the existence of three different dynamical patterns, one of which proved, in a statistical analysis based on results from previous works, to be significantly related with *SRF*-dependent regulation (p-value<0.05). The *chromatin immunoprecipitation (chip)* assay confirmed the rich presence of working *CArG* boxes in the genes belonging to the latter dynamical pattern and therefore validated the statistical analysis. Furthermore, an *in silico* analysis of the promoters revealed the presence of additional conserved *CArG* boxes upstream of the genes *Nr4a1* and *Rgs2*. The *chip* assay confirmed a significant *SRF* signal in the *Nr4a1 CArG* box but not in the *Rgs2 CArG* box.

## Introduction

Cognitive processes such as learning and memory originate from plastic modifications in the central nervous system CNS: these plastic changes affect the structure and the functions of neurons and of synapses and lead to experience-dependent alterations in neural network wiring and behavior. The introduction of high-throughput assays and large-scale approaches in neuroplasticity has contributed to encompass the broad extent of this phenomenon, which involves the cooperative interplay of numerous cellular processes that not only regulate the synaptic transmission itself but also cell survival [1], neuronal growth [2] and neurogenesis [3].

The modulation of gene transcription has proven to be playing a key role in neuroplasticity: increased synaptic activity leads to calcium influx into the post-synaptic spines, dendrites and soma, which activates calcium dependent signaling pathways that in turn regulate transcription factors within the nucleus [4] [5] [6]. In our previous work with dissociated rat neuronal cultures [5] we combined transcriptome profiling with electrophysiological recordings in order to describe the role of different calcium sources in the regulation of gene expression changes. The variations of calcium dynamics driven by synaptic activity, as well as the resulting activation/deactivation changes in the relative signaling pathways, have shown to be tightly regulated both in time [7] [8] and space [9] [10] [11]. For instance, the modulation of the neurotrophin *Bdnf* (brain derived neurotropic factor) gene expression, following synaptic activity, requires a series of phosphorylation/dephosphorylation steps of the transcription factors *CREB*, *MEF2* and *MEcp2* in order to keep the *Bdnf* expression bound to the desired dynamics [12]. The expression level of many other plasticity-related genes is governed by sophisticated controls of dynamics [13]: this result is often achieved thanks to the interplay of a large number of transcription factors and is often related to signaling changes which are triggered within a time-scale of minutes [14] [15] [16].

Alterations in the dynamical pattern of activity-induced programs may result in pathological states: for example, the removal of the phosphatase *MKP-1/DUSP1* negative feedback loop on the kinase *JNK* alters the proper *JNK*-activation dynamics and leads to the inability of forming new axonal branching during mice cortex development [17]. Despite the importance of the dynamical aspects of transcriptional changes, the information currently available is limited to time-courses with low temporal resolution, i.e. a few time points, and/or concerning a reduced number of genes, such as [15] [18] [19]. The purpose of the present study is to trace with high temporal resolution the early transcriptional dynamics associated with plasticity, using the gabazine treatment of rat organotypic cultures as hippocampal plasticity model: organotypic culture preparation has the advantage of retaining the general morphological and functional properties of the intact hippocampus [20] [21]. Besides, if compared to acute slices, organotypic cultures are able, within one week, to remodel the synaptic connections altered by the slicing procedure, which is not possible for acute slices [22]. In this work we will begin with a preliminary microarray-based assessment of the transcriptional response of hippocampal cultures to a 20 µM gabazine (also known as SR95531, a *GABA-A* receptor antagonist) treatment: the aim of this step is to obtain a general outline of the cellular activities involved in the response to *GABA-A* blocking. GABA-A channels are ionotropic channels that, upon binding of Gaba molecules, exert an inhibitory effect on neuronal excitability by specifically increasing the chloride conductance. Drugs such as gabazine, bicuculline or picrotoxin (PTX) act as GABA-A antagonists and therefore induce an increase of the overall neuronal excitability: these drugs have been extensively used as models for various types of plasticity (epilepsy, long term potentiation, homeostatic plasticity etc.), according to the tissue, dosage, duration of the treatment and possible concomitant stimuli. The 20 µM dosage was adopted in accordance to the evidences provided in [5], where we have previously studied the electrophysiological effects of a 20 µM GabT in dissociated hippocampal cultures.

Following the microarray assay, we will then quantify and analyze a high temporal resolution time course comprising a large set (33) of plasticity-related genes and we will relate the main features of the dynamical profiles with the putative biological functions of the relative genes/proteins. Following that, we will link one cluster of genes to a *SRF*-dependent regulation, by means of statistical and *in silico* analysis, and we will finally develop a *chip* (*chromatin immunoprecipitation*) assay in order to gain novel information about the role of *SRF* in the early phase of activity-dependent regulation of gene expression.

## Results

### Microarray analysis

A transcriptome profiling of a *GABA-A* receptor antagonist treatment is still lacking in the case of organotypic hippocampal cultures. Therefore, we decided to start the analysis with a preliminary, microarray-based, assessment of the response of rat organotypic hippocampal cultures to a 20 µ*M* gabazine treatment (GabT): the purpose of this step was to obtain a complete profile of the tissue reaction to a prolonged *GABA-A* receptor blockade, which is strictly associated with a sudden and powerful increase in the tissue synaptic activity and in the intensity of calcium dynamics [6] [23] [24].

Three independent biological replicas were collected and analyzed on the *Affymetrix rat 230.2 chip*; for each replicas the expression of the gabazine-treated sample was then compared to the control-untreated sample and the probes/genes of the chip were arranged in ascending up-regulation/p-value score. The results of a *GO* enrichment analysis, performed considering the genes with an up-regulation value higher than 2, approximately corresponding to a p-value≤0.005, are presented in table 1. The complete list of probes/genes data used in the present and in the subsequent analysis is provided in the table A in file S1.

**Table 1:** Table presenting the principal families of GO terms found to be enriched in the microarray-based analysis of gabazine treatment.

GO term	num. genes	p-value
*regulation of synaptic transmission*	7	1.90·10^−4^
*nucleus*	14	1.20·10^−2^
*regulation of transcription*	19	8.60·10^−4^
*positive regulation of transcription*	15	3.70·10^−7^
*learning or memory*	9	3.40·10^−7^
*feeding behavior*	5	6.40·10^−7^
*regulation of calcium ion transport*	5	3.60·10^−4^
*transmembrane protein*	6	1.20·10^−1^
*regulation of apoptosis*	11	2.20·10^−3^
*negative regulation of apoptosis*	6	2.80·10^−2^
*positive regulation of cell death*	6	2.80·10^−2^

The first column specifies the GO term, the second column contains the number of genes associated with the GO term and the last column presents the p-value score of the enrichment.

The sudden increase of synaptic activity induces the up-regulation of a variety of genes involved in several cellular processes and localized into different cellular compartments. A significant component (p-value≤1.90·10^−4^, modified Fisher Exact P-value) of the up-regulated genes, including for instance the effectors *Arc* and *Rgs2*, is involved in the regulation of synaptic transmission itself, by acting directly in axon terminals and dendritic spines. Another group of genes (p-value≤1.90·10^−5^) consists in a large pool of transcription factors, like for example *Cfos* and *Klf4*, that is responsible for driving the second wave of cellular responses, possibly related to longer lasting changes in neuron metabolism, morphology and functions [7]. Interestingly, the same group of transcription factors results to be highly enriched in the positive regulation of transcription term (p-value≤3.70·10^−7^): this indicates that, despite the presence of transcriptional repressors, such as *Icer* and *Nfil3*, the longer lasting changes are mainly based on the activation of not-expressed genes rather than on the suppression of already expresses ones. A consistent (p-value≤2.20·10^−3^) component of genes is involved in the regulation of cell survival: interestingly, according to the *GO*, they appear to influence the survival in both a positive and a negative manner. However, it appears that GabT treatment induces a strong push (p-value≤1.7·10^−2^) towards growth, neurogenesis and neuritogenesis. Finally, it is worth mentioning that the *MAPK* signaling pathway as well as the small-gtpase family are confirmed as the most important mediators of the aforementioned processes (p-value≤2.9·10^−2^).

To verify the up-regulation values observed in the microarray assay, we selected a group of 33 genes among the highest up-regulated ones and we measured their expression level in gabazine vs. untreated samples by RT-PCR. These 33 transcripts correspond to the top-fifty up-regulated probes deprived of those pointing to “*predicted*” transcripts and deprived of those characterize by low values of *mRNA* abundance (i.e. intensity of microarray signal). The latter ones were excluded mainly because their low amounts of *mRNA* were causing the RT-PCR data to be excessively noisy. The final list of transcripts whose up-regulation was verified by RT-PCR is presented in Table 1, while the RT-PCR data is presented in table C in file S1.

As a next step, we wanted to validate the previous Gene Ontology analysis. The functions associated to the genes in the Gene Ontology database (www.geneontology.org) are often derived from bioinformatics predictions, such as inference from sequence ortology or from common expression patterns: these kind of predictions, although likely reliable, have not been verified experimentally. In order to assess the consistency of our GO analysis, we proceeded by creating a manually compiled “vocabulary” of gene functions for each of the genes belonging to the set confirmed by RT-PCT; this vocabulary was based on an extensive search in the literature and built by considering only the most reliable results. More precisely, we preferentially considered only functional evidences derived from hippocampal tissues such as organotypic slices, acute slices, dissociated cultures or in vivo conditions. When hippocampus-based studies were lacking, we collected proofs from other types of nervous tissues, such as cortical neurons, dorsal root ganglion cells or glioma tissue. The complete list of gene/protein roles extracted from the literature is available in file S2, while a brief summary of them is available in Table 2.

**Table 2:** Table containing the list of 33 genes whose up-regulation was confirmed by RT-PCR.

GENE NAME	ROLE	FUNCTION(S)	REFERENCES
Arc	EF	NEUROGENESIS/SURVIVAL/anti-GROWTH/Reg.Syn.trasmission	[25] [26] [27] [28] [29] [30] [31] [32] [33] [34]
Atf3	TF	SURVIVAL	[1] [35] [36]
Bdnf	EF	NEUROGENESIS/SURVIVAL/GROWTH/Pos.Reg.Syn.transmission	[37] [38] [39] [40] [41] [42] [43] [44] [45] [46]
Btg2	TF	NEUROGENESIS/SURVIVAL	[1] [3] [47] [48] [49]
Cfos	TF	GROWTH/Pos.Reg.Syn.transmission	[18] [50] [51] [52] [53] [54] [55] [56] [57]
Cited2	TF		[58] [59] [60]
Crem/Icer	TF	anti-SURVIVAL/Neg.Reg.Syn.transmission	[61] [62] [63] [64] [65] [66] [67]
Cyr61	EF	GROWTH	[68] [69] [70]
Dusp1	EF	erk inactivation/anti-GROWTH	[17] [71] [72]
Dusp5	EF	erk inactivation	[73] [74]
Dusp6	EF	erk inactivation	[75] [76]
Egr1	TF	Pos.Reg.Syn.transmission	[77] [78] [79] [80] [81] [82]
Egr2	TF		[83] [84]
Egr3	TF	Pos.Reg.Syn.transmission	[82] [85] [86] [87]
Egr4	TF		[88] [89] [90]
Gadd45b	EF	NEUROGENESIS/SURVIVAL/GROWTH	[1] [2]
Homer1a	EF	Reg.Syn.trasmission/GROWTH	[91] [92] [93] [94] [95]
Irs2	EF	GROWTH/Reg.Syn.trasmission	[96] [97] [98]
Klf4	TF	anti-GROWTH	[99] [100] [101] [102] [103]
Mapk10	EF	SURVIVAL/GROWTH/Reg. Syn. Transmission	[104] [105] [106] [107] [108] [109] [110] [111] [112] [113] [114]
Nfil3	TF	SURVIVAL	[115] [116] [117]
Nptx2	EF	Neg.Reg.Syn.Transmission	[118] [119] [120] [121]
Npy1r	EF	NEUROGENESIS/Pos.Reg.Syn.trasmission	[122] [123] [124] [125] [126]
Nr4a1	TF	SURVIVAL/anti-GROWTH	[1] [127] [128]
Nr4a2	TF	SURVIVAL/Reg.Syn.transmission	[81] [128] [129] [130] [131] [132]
Nr4a3	TF	SURVIVAL/GROWTH	[19] [128] [133] [134]
NTF3	EF	GROWTH/SURVIVAL	[135] [136] [137] [138] [139] [140] [141] [142]
Pcdh8	EF	Neg.Reg.Syn.Transmission	[143] [144]
Plk2	EF	Neg.Reg.Syn.Transmission	[24] [145]
Ptgs2	EF	SURVIVAL/Pos.Reg.Syn.Transmission	[146] [147] [148] [149] [150] [151]
Rasl11b	EF		[152]
Rgs2	EF	Neg.Reg.Syn.Transmission	[153] [154] [155] [156]
Srf	TF	Reg.Syn. Transmission	[12] [69] [72] [157] [158] [159] [160] [161]

The first column contains the official gene symbol, the second column assigns the role of EF, effector, or TF, transcription factor, while the last column summarizes the validated gene functions, which always refer to tissues or experimental conditions coherent with the present work. For the complete details please refer to the survey presented in the supplementary file S2.

**Figure 1:**
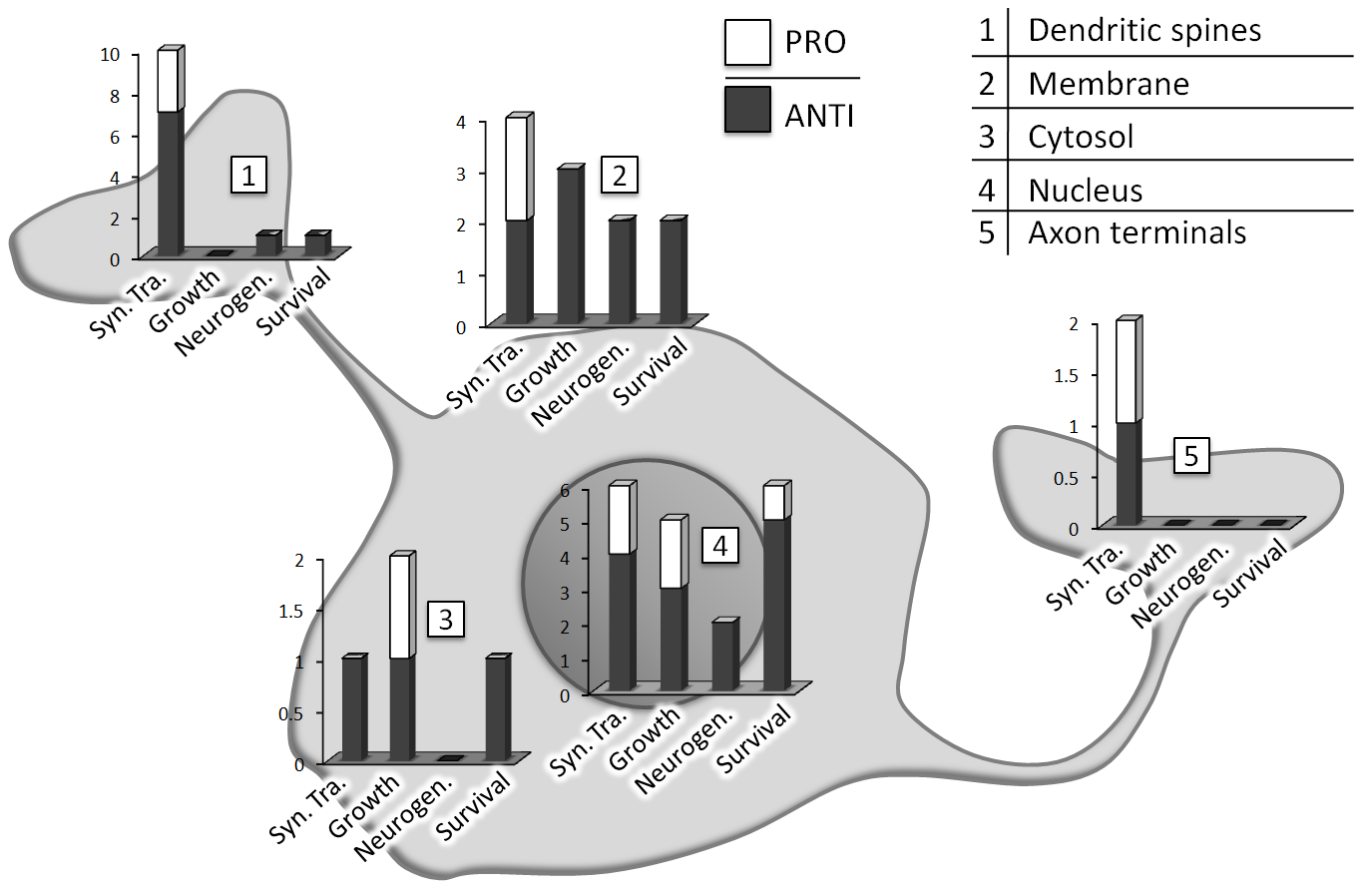
Cellular localization of the functions attributable to the genes up-regulated by GABAr blockage. Gene functions, extracted from findings reported in the literature, are categorized in four main groups, indicated by the terms: Syn.Tra, regulation of synaptic transmission, growth, regulation of growth, neurogen., regulation of hippocampal neurogenesis, survival, regulation of survival. Each gene is counted as PRO when it positively regulates these processes, while it is counted as ANTI when it negatively affects these processes. The cellular compartment of action for each protein(gene) is chosen according to the indications reported in the literature. For example, it has been shown that the pentraxin Nptx2 (neuronal activity–regulated pentraxin) is localized in the excitatory synapses, where it exerts a homeostatic effect by recruiting AMPAr, AMPA receptors, at excitatory projections onto gabaergic interneurons [118]–[121].

Since it is well established that certain genes/proteins listed in Table 2 can exert different roles according to the cellular context [1] [110] [162] (see file S2 for more details), we also tried to avoid considering functional results obtained from excessive pathological stimuli, which could alter the physiological native role of a gene/protein. For instance in [60] the neurons were treated with *Camptothecin* to cause DNA damage and the *Cbp/p300-interacting transactivator 2*, also known as *Cited2*, was related to the activation of apoptosis: we found these circumstances too dissimilar from the gabazine-treatment of the present work and therefore we decided not to consider this as a functional evidence. The Fig. 1 represents the distribution of the literature-extrapolated functions with respect to the cellular compartments. The similarity between the functions/processes highlighted by GO and those derived from selected literature appears to be good, nonetheless we can make at least two considerations:

1. In regard to the equilibrium of pro and anti-survival genes emerged from the GO, we must point out that the resulting situation from the literature analysis is quite different: instead of an equilibrium, we can actually notice a substantial shift towards pro-survival genes in response to gabazine. This difference arises from a different attribution of functions to the genes *Nr4a1*, *Ptgs2*, *Arc*, *Atf3*, *Gadd45β* and *Nfil3*. More precisely, all of these genes have proven, in the past years, to consistently promote neuron survival by protecting them from various oxidative, genotoxic and exitotoxic stresses; see file S2 for a complete review. (In short, we can confirm that a strong neuroprotective shield is induced by the synaptic activity associated with *GABA-Ar* blockage.)
2. Fig. 1 depicts more clearly how the effector early genes induced by the GABA-A blockade are mainly involved in the regulation of synaptic transmission and are localized in the synaptic terminals. Vice versa, those genes with growth, survival and neurogenesis promoting effects are mainly acting in the nucleus as transcription factors, thus their effects will realize only in conjunction with the subsequent wave of up-regulated genes.

### Gene expression time course

To gain better insights into the mechanisms of the transcriptional response to GabT we decided to investigate whether the up-regulation value found after 1.5 hours (for the genes induced by gabazine) is reached following different temporal dynamics or, on the contrary, all genes share the same induction pattern.

Previous studies [50] [163] [164] have already suggested that, following episodes of synaptic activity or during synaptic plasticity processes, the induced immediate-early-genes (IEGs) are characterized by different up-regulation dynamics: nonetheless, the time-course data collected so far in the literature is mainly obtained by microarray analysis, such as [7] [165], and not by a reliable and accurate RT-PCR analysis: more precisely, the information currently available is limited to time-courses with low temporal resolution, i.e. a few time points, and/or concerning a reduced number of genes, such as [15] [18] [19]. In all of these cases the time-course measurement was not the main aim of the paper, but it was rather an instrument to verify the effects of certain blockers/conditions, therefore a particularly high temporal resolution was simply not needed.

**Figure 2:**
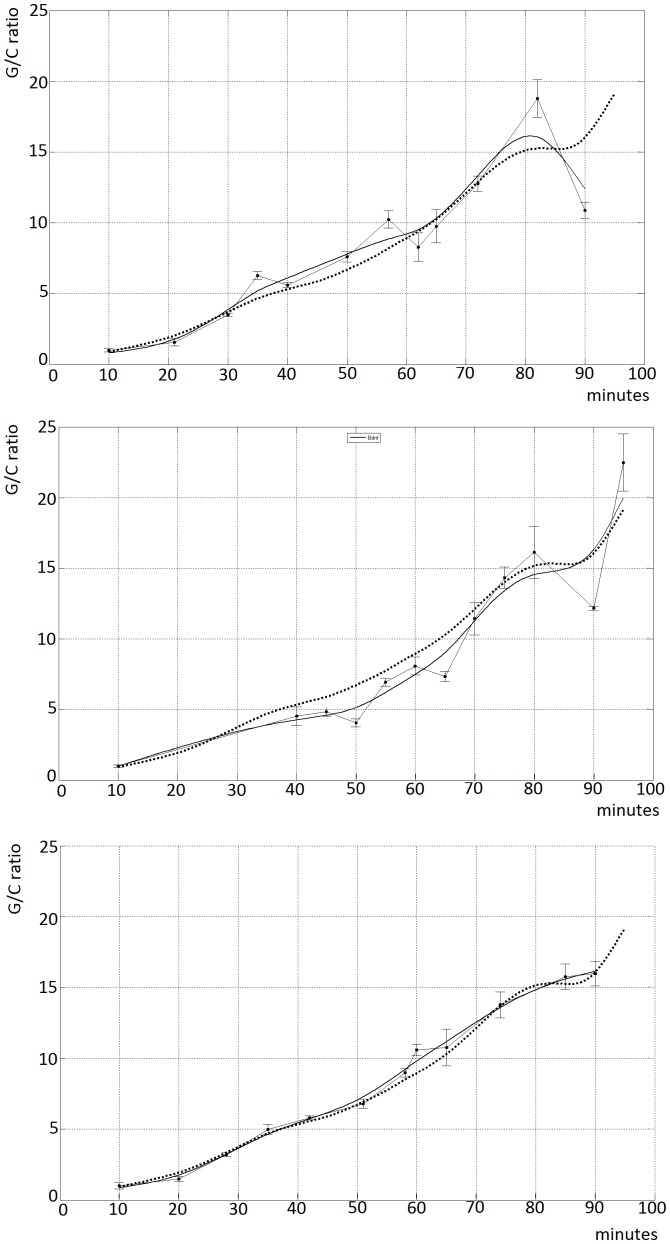
Bdnf time course: Graphs representing the three independent replicas of the Bdnf mRna time course. Time (minutes) on the x-axis, ratio Gabazine/Control on the y-axis. The error-bar plot refers directly to the RT-PCR data. In each of the sub-pictures, it is presented the trend resulting from the single biological replica of the RT-PCR time-course, which is superimposed to the averaged one, calculated as the mean of the three replicates. Thanks to this representation, it is possible to directly compare the single measures (biological replicates) to the averaged one, which eventually was the one used for all of the subsequent clustering analysis. The neurotrophin *Bdnf*, one of the master regulators of learning and memory, will prove in the end to be up-regulated according to a pattern which is representative for almost 50% of the genes of the set under study.

The rat organotypic hippocampal cultures were subjected to a 20 µ*M* gabazine treatment and the total Rna was gathered at 12 different time points spanning from 10 minutes to 95 minutes, with an average inter-sample time (sampling period) of 10 minutes. The procedure was eventually repeated three times, each time with a different twin rats couple, in order to obtain three independent replicas of the time-course, and RT-PCR was then performed for every gene in order to measure the up-regulation values at the different time points. The genes included in the time course analysis are those presented in Table 2. The time points of each replicas were then interpolated with a smoothing spline in order to emphasize the major trend underlying the up-regulation process; afterwards, the three interpolations derived from the replicas were combined into an average one, which was considered as the reference trend in all of the subsequent analysis. As an example, the resulting time course for the *Bdnf* gene (exon IV ) is shown in Fig. 2, together with the original and interpolated results for each replicas.

The first step of analysis that we carried out was a clustering of the temporal data, aimed to unveil the existence of distinct temporal patterns. Given that the measured time series are highly non stationary, we decided to discard correlation-based methods in favor of a k-means clustering algorithm based on Euclidean-distance; after a preliminary normalization, which reduced all the expression values of each gene to the interval [0∶1], the Euclidean-distance method proved to be able to correctly group together genes sharing a similar temporal pattern, regardless the absolute values of up-regulation. This methodology is the same applied in [164].

**Figure 3:**
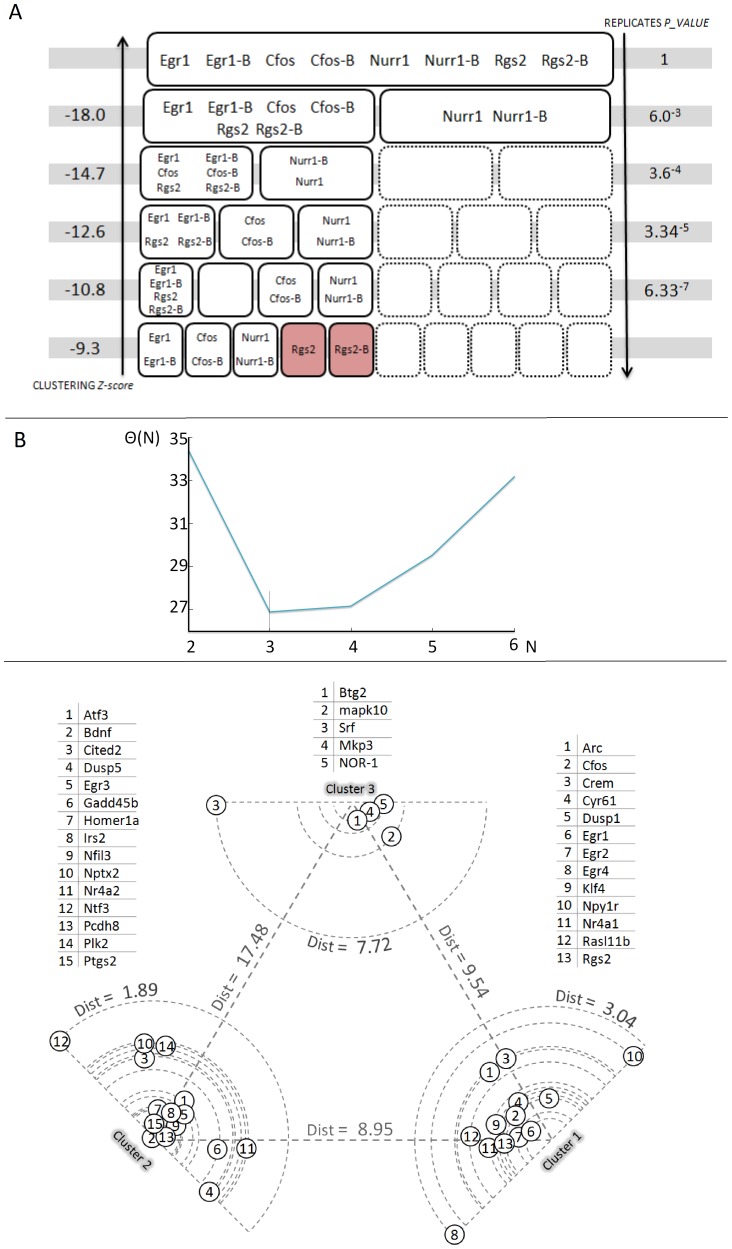
Analysis of the clustering quality for the time course data. A) Outcome of the clustering algorithm, with progressive increase in the number of clusters k: the picture represents, at each different k, the grouping of the 4 couples of alternative primers pointing to the same gene. For k = 2,4,6,8 the alternative primers were correctly grouped together. The “replicas p-value”, on the right, indicates the statistical consistency of the alternative primer grouping, which reaches it maximum value when the algorithm is forced to split the 33 genes into 8 different clusters. On the left, the Z-value of the global clustering, indicating the consistency of the temporal dynamics discrimination. B) Outcome of the algorithm aimed at determining the optimal value for k. The number of clusters N is plotted against a function Θ(N): the minimum of Θ(N), i.e. N = 3, coincides with the optimal value for k. See Materials and Methods section for further details. C) Visual representation, with k = 3, of the distances between trajectories and cluster centroids for all the 33 genes. For each cluster, the genes are disposed at increasing distances from the centroid, proportionally to their normalized Euclidean distances. The distance of the farthest gene is indicated in the proximity of the outer circle. The orientation of the genes reflects the proximity to the remaining two clusters. The distances between the cluster centroids are also indicated.

The main drawback of the k-means algorithm is the necessity to manually set k, i.e. the number of desired clusters [166]. The ability of the algorithm to distinguish among potential different temporal dynamics increases as k increases, but, on the contrary, the Z-score of the grouping outcome becomes less significant at higher k values, which means that a random grouping would have produced similar results, as illustrated in Fig. 3A.

To further test the consistency of the clustering procedure, we designed four new control primers for the genes *Egr1*, *Cfos*, *Rgs2* and *Nurr1*: these alternative primers point to different exons and different exon-exon junctions with respect to the original ones. With k = 2 the control primers were correctly grouped together with their counterparts, as highlighted in figure 3. Most importantly, even at higher fragmentation levels, with k = 4, k = 6 and k = 8, the control primers remain associated to the proper original ones: the probability that this correct grouping might be due to chance is p = 6.33·10^−7^ when k = 8.

We decided to use the approach described in [167] to determine the optimal value for k in a unsupervised manner; the method is based on the minimization of a function *Θ(N)*, where *N* is the number of clusters. Intuitively, the minimum of *Θ(N)* coincides with the number of clusters where the addition of a further one does not reduce significantly the average intra-cluster distance. More details about this approach are presented in the Materials and Methods section. The final result, presented in Fig. 3B, indicates that k = 3 is the optimal value for the cluster number. In Fig. 3C the outcome of the clusterization process with k = 3 is represented in a two-dimensional plane.

Cluster 1, which comprises genes such as *Arc*, *cFos* and *Klf4*, is characterized by a fast rise in the expression values, which peak at about 50 minutes and subsequently remains steady till the end of the measurement. The *Arc* gene was reported in several works to be rapidly induced by episodes of synaptic activity, with a peak within the first 60 minutes. Thus, for the *Arc* gene, our result is coherent with [168], [169] and [170]; furthermore, it extends the results to the other 12 IEGs characterized with the same dynamic of *Arc*, thus suggesting the existence of a common regulation system responsible for the induction of these faster-rising IEGs. Cluster 2, which comprises genes such as *Bdnf*, *Irs2* and *Homer1a*, is characterized instead by a slower but constant increase, almost linear up to 90 minutes. The differential dynamics characterizing the *Bdnf* gene (cluster 2) with respect to the *Cfos* and *Egr1* genes (cluster 1) are coherent with a previous study [15] of Schaffer-collateral HFS-induced LTP: again, here we extend the results to other 25 IEGs which result to be similar to *Cfos*/*Egr1* or to *Bdnf* dynamics. Besides, the longer lasting duration of *Cited2* (cluster 2) mRna up-regulation with respect to the faster and shorter up-regulation timings of *Cfos* (cluster 1) and *NOR-1* (cluster 3) also recalls the results obtained in [19] with an ECS stimulation of the Dentate gyrus. The last cluster, which is smaller than the previous ones and comprises genes such as *NOR-1* and *Btg2*, presents a marked peak which is concurrent to cluster 1 peak, but that is successively followed by a pronounced decrease of the expression value.

### Relationship between clustering and function

Since previous studies have already supported the notion that temporally clustered genes are likely involved in the same biological functions [165] [171], we next wanted to determine whether it was possible to relate the different temporal profiles previously extracted with particular inherent functions. Therefore, for each temporal cluster of gene expression we performed an enrichment analysis of functional evidence collected in the manually compiled vocabulary, introduced in the “*microarray analysis*” section.

**Figure 4:**
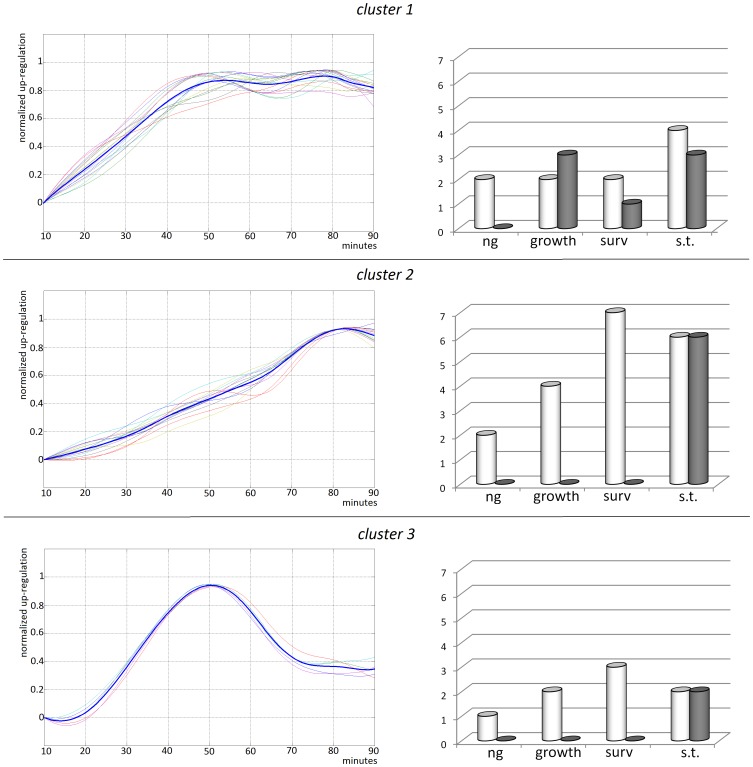
Graphs of time-course data and their associated functions. The bold line, representing the average temporal pattern of each cluster, is superimposed to the patterns of the single genes. The histograms depicts the amounts of positive (white bars) and negative (black bars) regulators of the indicated processes for each cluster: n.g., regulation of hippocampal neurogenesis, growth, regulation of growth, surv., regulation of survival, S.T., regulation of synaptic transmission.

The recent developments in the study of hippocampal plasticity have consolidated the idea that episodes of intense physiological synaptic activity strongly promote neurogenesis [3] [34] [37], growth [2] [97] [136] and survival [1] [36] [150]. Our data confirm the up-regulation of numerous genes endowed with these properties already in the early phase (10–90 minutes) of transcriptional regulation, see Fig. 4, indicating that a strong neuroprotective shield is quickly activated by synaptic activity in organotypic cultures, together with an increase in the Dentate Granule Cells neurogenesis and an increase in the growth rate of neurons and synaptic connections. However, also genes with negative effects on growth (namely *Icer*, *Klf4*, *Nr4a1* and *Mkp-1*) are induced in association with the mentioned majority of positive regulators, see Fig. 4. Interestingly, these four genes were all grouped in the cluster 1 temporal pattern, thus making the cluster 1 significantly enriched with anti-growth properties (p-value≤0.017, Fisher's exact test). Vice versa, the cluster 2 comprises only genes providing a positive effect on growth.

In the past decade the mechanisms involved in the homeostatic regulation of synaptic strength have emerged as a fundamental complement to Hebbian plasticity [172] [173] [174]. In the present work we report that in rat organotypic cultures, following chronic blockade of *GABA-Ar*, many genes involved in homeostatic-scaling (weakening) processes, namely *Narp*/*Nptx2*, *Arc*, *rgs2*, *arcadlin*, *plk2*, *Homer1a*, *Icer*, *Dusp1*, *Dusp3*, *Dusp6*, of which the single contributes to plasticity have been partially unveiled [24] [33] [67] [91] [121] [144] [154], are induced in concert already in the first minutes of synaptic activity, thus suggesting the existence of a sensitive and fast feedback mechanism that is activated almost contextually to the perturbation. As illustrated in Fig. 4, the homeostatic genes are equally spread among the three clusters (p-value≤0.43, Fisher's exact test) indicating that there is no particular relationship between the homeostatic function and the up-regulation timings in the early phase (0–90 min.) of the hippocampal response to perturbation. Interestingly, we noticed that the homeostatic genes are tightly associated, in every cluster, with genes exerting the opposite function, i.e. the potentiation of synaptic transmission, as depicted in Fig. 4. Therefore, differently from the survival and growth functions, for the regulation of synaptic transmission we observe a functional equilibrium between homeostatic-plasticity (weakening) genes and Hebbian-plasticity (potentiation) genes.

Another crucial step of the homeostatic response is the re-establishment of the basal level of active *MAPKs* [15] [111]; this process is carried out mainly by means of a negative feedback loop involving the *MAPKs* themselves, together with the *Dusp* family of phosphatases [72] [76] [134]. Here we report that *Dusp1*, *Dusp5* and *Dusp6* are induced together by GabT, but with different temporal patterns, since they are grouped into different clusters, see Fig. 4. This result, which is coherent with previous studies [175] [176], indicates that each of the *DUSPs* is dynamically tied to a different group of genes: in this way, each cluster of the induced genes is synchronized with a relative homeostatic feedback to the *MAPKs*.

The peculiar distribution of the *Dusp* family members, as well as the in-cluster balance between homeostatic and Hebbian plasticity genes, led us to notice that, concerning the regulation of synaptic transmission, genes endowed with different, but at the same time complementary/counterbalancing, functions seem to be bound together into the same temporal dynamics in order to favor global robustness of the system: indeed in this case the dysregulation of a pathway caused by a pathological state would not create excessive imbalances since the inner genes compensate each other. It is interesting to point out that the present observation about global stability recalls the conclusions of previous work [177], in which a bioinformatic analysis of the CA1 hippocampal intracellular pathways [178] revealed the existence of robustness, stability and adaptability properties.

### Relationship between clustering and regulation

In order to investigate the possible relationships between the different temporal patterns of gene induction and the regulators of gene transcription, we performed an accurate and extensive literature research aimed to recreate the complete network of pathways involved in the regulation of hippocampal gene transcription: the complete survey is available in file S2. By a cross comparison between pathways and transcription factors on one side and time-course patterns on the other side, it emerged that cluster 1, which was characterized by a fast increase in the expression values followed by a flat/stationary state, is particularly enriched in *SRF*, serum response factor, dependent regulations (p-value≤0.02, Fisher's exact test). On the contrary, the cluster 2 does not present any *SRF* dependent regulation (p - value≤0.05 ). This data indicates that the *SRF* dependent regulation is consistently biased towards the cluster 1, which is the cluster of genes such as *Arc*, *Cfos*, *Cyr61*, *Egr1* and *Egr2*, all of which have shown to be regulated by Serum Response Factor in various plasticity models [69] [179] [180] [181] [182] [183].

**Figure 5:**
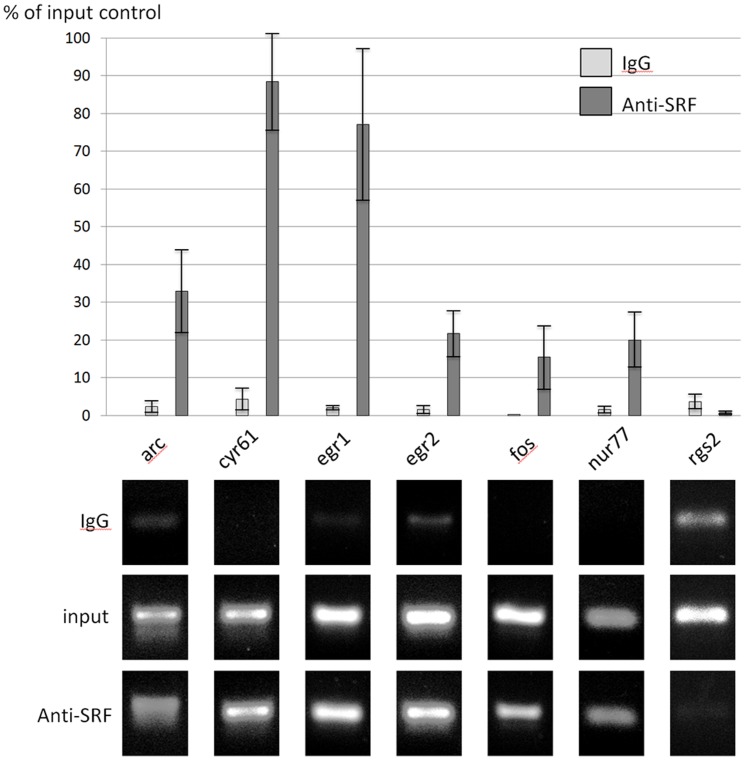
Analysis of SRF binding sites by chromatin immunoprecipitation. Chromatin fragments of hippocampal organotypic cultures were immunoprecipitated with anti-SRF antibody. A) Immunoprecipitation levels normalized to input control: the s.e.m. is calculated over three different replicas. B) Immunoprecipitation of each promoter region, together with input control and IgG antibody, was amplified by PCR. Each sample is derived from three independent replicas.

To assess the validity of the above mentioned SRF regulatory evidence for the genes *Arc*, *Cfos*, *Cyr61*, *Egr1* and *Egr2* in our model of hippocampal plasticity, i.e. GabT of organotypic cultures, we performed *chip* (*chromatin immunoprecipitation*) experiments to detect *SRF* binding levels in their promoters during GabT. Besides, we carried out an *in silico* analysis of the promoters of the remaining 8 genes belonging to the same cluster in order to detect other possible active *CArG* boxes, the DNA sequence motif *CC[A/T]_6_GG* that has a high affinity for SRF. As a result, *CArG* boxes conserved among humans, rats and mice were found in the upstream region of *RGS2* and *NR4A1* genes, respectively at −5 kb and −123-111; therefore, those genes were included in the chip experiment together with the previous ones. The results of *chip*, presented in Fig. 5, show that a strong SRF signal was detected in *Arc*, *Cfos*, *Cyr61*, *Egr1* and *Egr2* and *Nr4a1* while no significant signal was found for *RGS2*, indicating that the latter gene is likely not to be regulated by *SRF* in our plasticity model.

## Discussion

The present article identifies three different dynamical patterns in the early-phase (10–90 min) of the transcriptional response induced by GabT of organotypic hippocampal cultures and provides novel information about the role of Serum Response Factor. The blockage of GABA-A ionotropic channels by means of gabazine/bicuculline/PTX is a widespread [1] [145] [184] [185] model of plasticity where the increased synaptic activity triggered by GabT leads to the up-regulation of a plethora of activity-dependent genes. While the electrophysiology of GABA-A antagonists in organotypic hippocampal cultures has been extensively studied [6] [24], the relative variations in the transcriptome have been so far conducted in dissociated cultures [1] [5] [7] [186]. This last aspect prompted us to develop a preliminary assessment with a microarray-based transcriptome profiling.

The Gene Ontology analysis of microarray data revealed that the major functions of the 346 genes up-regulated by GabT (p-value≤0.005) are related to the regulation of synaptic transmission, calcium ions transport, transcription, apoptosis, feeding behavior, learning and memory. With respect to apoptosis regulation, the GO analysis further indicates that both positive and negative regulators of survival are up-regulated in organotypic cultures and therefore the general effects of GabT on cell fate seems not to be predictable. Nevertheless, a manual annotation of the gene functions actually revealed that GabT promotes a push towards survival, neurogenesis and neuroprotection, confirming the results obtained in dissociated cultures [1] [185] and extending them to the case of hippocampal organotypic cultures.

To further investigate the dynamics underlying the early-phase of the regulation of activity-dependent genes, we developed the quantification of a high temporal resolution time course, ranging from 10 to 90 minutes, with an average inter-sample time of 10 minutes. The trajectories of the 33 genes included in the time-course were subjected to a unsupervised k-means clustering: the unsupervised clustering identified three different dynamical patterns, as depicted in Fig. 3 and Fig. 4. By crossing the cluster grouping with the gene functions listed in the manually compiled vocabulary (see file S2) we found that the group of genes characterized by a fast rise to a plateau value (cluster 1) seems to be significantly (p-value<0.05) provided with anti-growth and anti-survival properties. Since this cluster is characterized by the fastest response, peaking already at 50 minutes, this data suggests that a rapid activation of negative regulators of growth, possibly involved in the initial disassembly of existent structures, is subsequently followed by an induction of growth promoting genes (cluster 2, slow up-regulation).

Besides, we also found that cluster 1 is also enriched in *SRF*, serum response factor, dependent regulations (p-value≤0.02, Fisher's exact test). Interestingly, in a previous work [187] with dissociated cultures we showed that the genes *Nr4a1*, *Arc*, *Egr1*, *Egr2* and *Egr3*, which belong, in the present paper, to cluster 1 (with the exception of Egr3), are characterized by a marked dependence on *MAPK*-dependent regulation when compared to *Bdnf* and *Homer1a*, which instead here belong to cluster 2. Moreover, a strong dependence *Dusp1* and *Fos*, which again belong to cluster 1, on *MAPK* regulation was previously emphasized in rat neuroendocrine cells [188] [189]. These data suggest that the cluster is particularly dependent on *SRF*/*MAPK* and motivated us to investigate whether the aforementioned *SRF* dependent regulations, which were extrapolated from the literature and derived from different experimental conditions, are still valid in the case of GabT of organotypic cultures.

To this end, we performed chip, chromatin immunoprecipitation, for detecting *SRF* binding levels during GabT and we found that *Cyr61*, *Egr1*, *Egr2*, *Fos* and *Arc* present a significant *SRF* binding signal. While genes *Fos* and *Egr1* have already been reported to be regulated by *SRF* in hippocampal organotypic cultures [179], ours is the first report for genes *Cyr61*, *Arc* and *Egr2*.

To complete the survey of working *CArG* boxes in cluster 1, we analyzed the sequences upstream of *TSS* for the remaining genes and we found conserved *CArG* boxes also upstream of *Rgs2* and *Nr4a1*. Eventually, the chip assay revealed that the *Nr4a1 CArG* box presents a significant SRF signal while no signal was found for *Rgs2*. This result is interesting in particular for *Nr4a1* gene, for which the functionality of the aforementioned *CArG* box has so far provided motley evidences. In fact, in serum stimulation of *NIH-3T3* fibroblasts [190] and platelet-derived growth factor (*PDGF*) stimulation of *T98G*-glioblastoma [181] the *CArG* box has proven to be functional but in hippocampal neuronal cultures [191] [192], cerebellar cortex [127] and in vivo [193] conditions general findings are in favor of a *CREB* and *MEF2* determinant role. Therefore, our latter result suggests that, in organotypic cultures, *SRF* may play a role in the regulation of *Nr4a1* gene during the intense synaptic activity triggered by GabT.

In conclusion, this study provides novel insights into the early dynamics of transcriptional regulation in a plasticity model, showing how a large group of co-expressed activity-dependent genes is characterized by consistently different patterns of induction in the first 90 minutes of tissue response and linking these patterns to different inherent functions and regulatory mechanisms. We believe that unveiling the finest tuning in the regulatory dynamics of plasticity is the key step to gain a more quantitative awareness of the phenomenon.

## Materials and Methods

### Ethics Statement

Rat hippocampi were dissected from Wistar rats (P4–P5), in accordance with the regulations of the Italian Animal Welfare Act, and the procedure was approved by the local authority veterinary service (Dr. R Zucca). Every possible effort was taken in order to minimize both the number and the suffering of used animals. The experiments were carried out in accordance with the European Communities Council Directive of 24 November 1986 (86/609/EEC) and formal approval for experimental procedures was provided by the Ministry of Health(protocol 13/97–A).

### Tissue, pharmacology and Rna extraction

Rat hippocampi were dissected from Wistar rats (P4–P5). Organotypic cultures were prepared following the roller tube method [194]. Gabazine was purchased from Tocris (Bristol, UK). Gabazine treatment (GabT) for microarray samples consisted in treating the cultures for 90 min with 20 µ*M* of gabazine, a specific GABA-A receptor antagonist [195]. Gabazine treatment (GabT) for time course samples consisted in treating the cultures with 20 µ*M* of gabazine for a variable time with time samples ranging from 10 minutes to 90 minutes. The total RNA for the microarray samples and the time-course samples was extracted using the TRIzol reagent (Sigma, Milano, Italy) according to the manufacturer's instructions followed by a DNase I (Invitrogen, Carlsbad, California, USA) treatment to remove any genomic DNA contamination. The total RNA was further purified using RNeasy Mini Kit Column (Qiagen, Valencia, CA) and subsequently quantified by ND-1000 Nanodrop spectrophotometer (Agilent Technologies, Palo Alto, CA).

### Analysis of Microarray data and P-value calculation

For the microarray data, three biological replicas were collected at 90 min of GabT and Standard Affymetrix protocols were applied for amplification and hybridization. Gene profiling was carried out with the Affymetrix RAT2302 GeneChip containing 31099 probes, corresponding to 14181 probes with a gene symbol. Low level analysis was performed using an Robust Multi-array Average (RMA) algorithm [196] directly on the scanned images.

Data were organized in matrices “*m*×*n*” (*m*, number of genes; *n*, number of replicas). Two samples were considered: an untreated culture (*C_ij_: i = 1,..,n j = 1,..,m*), a culture treated with gabazine (*G_ij_*). Data were analyzed by considering log_2_ changes of gene expression in each replicas against its own untreated control, that is, log_2_ (*G_ij_/C_ij_*). Thus, from the microarray data we obtained an “*m*×*n*” ratio-matrix for each treatment. Considering the three replicas as independent variables, this matrix was treated as a multivariate variable in three dimensions. We derived the empirical cumulative distribution function with upper and lower bounds of the multivariate variable, using the Kaplan–Meier estimator (Kaplan and Meier, 1958) so to assign a p-value to all the genes and select the most significant ones. The microarray data can be found in the GEO database, accession number: GSE46864.

### GO enrichment analysis

GO enrichment analysis for microarray data was performed with Gene David [197] (http://david.abcc.ncifcrf.gov/). GO analysis for the manually annotated vocabulary was performed according to the following formulas:

The probability to have exactly 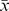 genes characterized with a certain “GO term” (for example, “SRF regulation” or “positive regulation of synaptic transmission”), in a cluster of dimension *n*, is
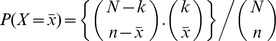
Where N is the total number of genes(elements), n is the dimension of the cluster, k is the total number of genes(elements) which present the “GO term” under consideration. The cumulative probability to have an amount of terms equal or higher than 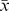, in a cluster of dimension *n*, is
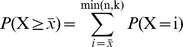



### Quantitative RT-PCR and time-course analysis

For the time course experiment, the expression level of the target mRNA was quantified bt RT-PCR. RNA (250 ng) was reverse-transcribed using SuperScript II reverse transcriptase and random hexamer (Invitrogen). qRT-PCR was performed using iQ SYBR Green supermix (Bio-Rad, Munich, Germany) and the iQ5 LightCycler (Bio-Rad). Gene specific primers were designed using Primer3 [198](http://frodo.wi.mit.edu/). The thermal cycling conditions comprised 3 min at 95C, and 45 cycles of 10 sec for denaturation at 95C and 45 sec for annealing and extension at 58C. The expression level of the target mRNA was normalized to the relative ratio of the expression of Gapdh mRNA. Fold change calculations were made between treated and untreated samples at each time point using the DDCT method. Three organotypic cultures were used for each sample. The 36 primers used for the time course analysis are provided in table B in file S1.

The resulting time-course data-set consists of three biological replicas, each one containing 12 time points ranging from 10 m to 90 m. Each raw data time-course replicas obtained from RT-PCR data was independently fitted with a smoothing spline (Matlab environment) and normalized to the [0∶1] interval. Subsequently, the three replicas were jointed together and analyzed via a k-means clustering, based on Euclidean distance (same method as [[164]]). To identify the optimal number of clusters we adopted the approach proposed in [167]. Briefly, a function
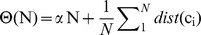
is computed at every *k*, i.e. cluster number. *N* is the number of clusters, *dist(c_i_)* is the intra-cluster distance, i.e. the scaled average squared distance between shapes in the cluster *c_i_* and *α* is a parameter controlling the grain of the clustering. The minimum of the function *Θ(N)* corresponds to the optimal number of clusters.

The enrichment score for the transcription factors regulatory evidences was computed using the same approach described in one of the previous section, “Analysis of Microarray data”.

### Identification of upstream sequences and transcription factor binding sites

The 10 k-bp upstream regions for mouse, rat and human of the cluster 1 genes were extracted from *mapviewer* (http://www.ncbi.nlm.nih.gov/mapview/). To identify the putative transcription factor binding sites within each upstream sequence, a preliminary verification of the conserved regions among mouse, rat and human was performed by aligning the sequences with *blast-bl2seq* (http://blast.ncbi.nlm.nih.gov/Blast.cgi), using a word letter size 16. To refine the blast results a further analysis was carried out with *Evoprinter* (http://evoprinter.ninds.nih.gov/) [199]. Finally, conserved domains were analyzed with *Jaspar* [200] (http://jaspar.cgb.ki.se/), using the MA0083.1 *SRF* binding matrix with a threshold score of 0.8.

### Chromatin immunoprecipitation

The chromatin immunoprecipitation assay was performed using the MAGnify Chromatin Immunoprecipitation System (Invitrogen, Catalog Number49-2024) according to the manufacturer's instructions with slight modifications. Briefly, organotypic cultures (ten cultures per condition) were cross-linked at room temperature, immediately after the GabT, using a PBS solution with formaldehyde 1%. Shearing was performed with a MSE Soniprep 150 (7 pulses of 5 seconds) to yield an average length of 300 bp. Samples were immunoprecipitated with 10 ug of anti-SRF antibody (Santa Cruz Biotechnology, Heidelberg, Germany, cat.no sc-335x) and with 1 ug of anti-rabbit IgG negative control antibody. Promoter specific primers were used for amplification:

*Nr4a1 Forward*: 5′-TTAAGAGGTGGGTCGGGTTC-3′

*Reverse*: 5′-GCAATCCTTCTCGCACACTA-3′

*C-fos*: *Forward*: 5′-CTCGCCTTCTCTGCCTTTC-3′

*Reverse*: 5′-GTAGGATTTCGGGGATGGTT-3′

*Egr1*: *Forward*: 5′-TGGGAGGTCTTCACGTCACT-3′

*Reverse*: 5′-GAATCGGCCTCTATTTCAAGG-3′

*Egr2*: *Forward*: 5′-ATGTGACCGGCAAAAGCTAC-3′

*Reverse*: 5′-AATGAATCGCTGCTCTCTCAG-3′

*Cyr61*: *Forward*: 5′-TCAAGAATGCCTTGTGGTTG-3′

*Reverse*: 5′-ACGGGGTAGAAGGAGGTGAT-3′

*Rgs2*: *Forward*: 5′-TGCCACCCCAGTAGTTACG-3′

*Reverse*: 5′-TTTGCCGAGAGATGAACAGA-3′

*Arc*: *Forward*: 5′-GTGGGGAAGCTCCTTGCT-3′

*Reverse*: 5′-CCAGTTAGAGGGCGGTGTT-3′

## Supporting Information

File S1 **Supplementary tables.** - Supplementary Table A: The complete list of microarray probes/genes. - Supplementary Table B: List of primers used for the time course analysis. - Supplementary Table C: RT-PCR validation data.
(DOCX)

File S2 **Supplementary notes.**
(DOCX)
