# Supplementary material for: Early Phase of Plasticity-Related Gene Regulation and SRF Dependent Transcription in the Hippocampus
Source: PLoS One. 2013 Jul 23;8(7):e68078. doi: 10.1371/journal.pone.0068078 (PMC3720722; doi:10.1371/journal.pone.0068078)
Supplement: File S1 — Supplementary tables. - Supplementary Table A: The complete list of microarray probes/genes. - Supplementary Table B: List of primers used for the time course analysis. - Supplementary Table C: RT-PCR validation data. (DOCX) [file pone.0068078.s001.docx]

**SUPPLEMENTARY TABLES for**

**Early phase of plasticity-related gene regulation and SRF dependent transcription in the hippocampus.**

*Giovanni Iacono^1^, Claudio Altafini^1^ and Vincent Torre^1,2,*^*

**Table A** Microarray results

**Table B** List of primers used in the time-course analysis

**Table C** RT-PCR data

**Table A**

Microarray results: the first three columns present the information concerning the affymetrix probe details (probe_ID, gene name and gene description). The columns “Abs.V.” contains the absolute values of the probe intensities, averaged over the theee biological replicates. The last column, “Ratio”, contains the ratio between gabazine treated and untreated samples, averaged over the three biological replicates.

| **Probe_ID** | **Name** | **Description** | **Abs.V.** | **Ratio** |
| --- | --- | --- | --- | --- |
| 1369217_at | Nr4a3 | nuclear receptor subfamily 4, group A, member 3 | 20,43 | 10,49 |
| 1387410_at | Nr4a2 | nuclear receptor subfamily 4, group A, member 2 | 18,51 | 9,49 |
| 1373759_at | --- | Transcribed locus | 21,72 | 8,86 |
| 1370997_at | Homer1 | homer homolog 1 (Drosophila) | 22,04 | 8,50 |
| 1392108_at | --- | RM2 mRNA, partial sequence | 20,71 | 7,85 |
| 1387068_at | Arc | activity regulated cytoskeletal-associated protein | 22,63 | 6,88 |
| 1386935_at | Nr4a1 | nuclear receptor subfamily 4, group A, member 1 | 22,45 | 6,20 |
| 1369007_at | Nr4a2 | nuclear receptor subfamily 4, group A, member 2 | 22,07 | 5,34 |
| 1386995_at | Btg2 | B-cell translocation gene 2, anti-proliferative | 18,79 | 5,32 |
| 1368144_at | Rgs2 | regulator of G-protein signaling 2 | 21,41 | 5,06 |
| 1387788_at | Junb | Jun-B oncogene | 20,76 | 5,00 |
| 1387306_a_at | Egr2 | early growth response 2 | 23,64 | 4,89 |
| 1380383_at | Arf4l_predicted | ADP-ribosylation factor 4-like (predicted) | 20,61 | 4,87 |
| 1385757_at | --- | Activity and neurotransmitter-induced early gene 1 (ania-1) mRNA, 3UTR | 20,44 | 4,86 |
| 1387442_at | Egr4 | early growth response 4 | 21,37 | 4,72 |
| 1383860_at | Fosl2 | Fos-like antigen 2 | 18,36 | 4,58 |
| 1396563_at | Nr4a3 | Nuclear receptor subfamily 4, group A, member 3 | 15,94 | 4,53 |
| 1386994_at | Btg2 | B-cell translocation gene 2, anti-proliferative | 21,46 | 4,51 |
| 1368146_at | Dusp1 | dual specificity phosphatase 1 | 22,57 | 4,21 |
| 1388792_at | Gadd45g | growth arrest and DNA-damage-inducible 45 gamma | 22,52 | 4,09 |
| 1368527_at | Ptgs2 | prostaglandin-endoperoxide synthase 2 | 21,05 | 3,90 |
| 1397999_at | Irs2 | Insulin receptor substrate 2 | 17,33 | 3,74 |
| 1368487_at | Serpinb2 | serine (or cysteine) proteinase inhibitor, clade B, member 2 | 11,35 | 3,73 |
| 1383354_a_at | Fbxo33_predicted | F-box only protein 33 (predicted) | 20,72 | 3,68 |
| 1368147_at | Dusp1 | dual specificity phosphatase 1 | 13,75 | 3,64 |
| 1396114_at | Fbxo33_predicted | F-box only protein 33 (predicted) | 14,49 | 3,32 |
| 1386152_at | Atoh7_predicted | atonal homolog 7 (Drosophila) (predicted) | 15,29 | 3,26 |
| 1385110_at | RGD1311122 | Similar to RIKEN cDNA 1110003E01 | 18,36 | 3,17 |
| 1387074_at | Rgs2 | regulator of G-protein signaling 2 | 25,32 | 3,15 |
| 1368956_at | Pcdh8 | protocadherin 8 | 20,33 | 3,12 |
| 1396891_at | RGD1564957_predicted | Similar to RIKEN cDNA 3110007P09 (predicted) | 18,79 | 3,09 |
| 1393782_at | Plagl1 | Pleiomorphic adenoma gene-like 1 | 13,42 | 3,08 |
| 1368124_at | Dusp5 | dual specificity phosphatase 5 | 18,81 | 3,08 |
| 1367598_at | Ttr | transthyretin | 19,99 | 3,03 |
| 1385407_at | Tiparp_predicted | TCDD-inducible poly(ADP-ribose) polymerase (predicted) | 14,33 | 2,95 |
| 1379401_a_at | Fbxo33_predicted | F-box only protein 33 (predicted) | 21,72 | 2,95 |
| 1385023_at | --- | --- | 15,19 | 2,95 |
| 1368290_at | Cyr61 | cysteine rich protein 61 | 18,97 | 2,88 |
| 1380020_at | Ppig | Peptidylprolyl isomerase G | 13,54 | 2,78 |
| 1381430_at | Numbl | Numb-like | 11,03 | 2,73 |
| 1376569_at | Klf2_predicted | Kruppel-like factor 2 (lung) (predicted) | 20,05 | 2,72 |
| 1370652_at | Ntrk2 | neurotrophic tyrosine kinase, receptor, type 2 | 15,42 | 2,71 |
| 1368677_at | Bdnf | brain derived neurotrophic factor | 22,75 | 2,67 |
| 1381903_at | Fbxo33_predicted | F-box only protein 33 (predicted) | 23,70 | 2,63 |
| 1372389_at | Ier2 | immediate early response 2 | 21,44 | 2,62 |
| 1397823_at | Cacna2d1 | Calcium channel, voltage-dependent, alpha2/delta subunit 1 | 18,61 | 2,60 |
| 1368321_at | Egr1 | early growth response 1 | 26,75 | 2,49 |
| 1380969_at | RGD1564670_predicted | Similar to mKIAA0940 protein (predicted) | 17,02 | 2,45 |
| 1370454_at | Homer1 | homer homolog 1 (Drosophila) | 22,59 | 2,44 |
| 1381498_at | Arf4 | ADP-ribosylation factor 4 | 13,92 | 2,42 |
| 1387530_a_at | Fosb /// Fosl2 | fos-like antigen 2 /// FBJ osteosarcoma oncogene B | 15,00 | 2,39 |
| 1368488_at | Nfil3 | nuclear factor, interleukin 3 regulated | 21,54 | 2,38 |
| 1391643_at | --- | --- | 20,46 | 2,38 |
| 1398266_a_at | Egr2 | early growth response 2 | 12,18 | 2,37 |
| 1386041_a_at | Klf2_predicted | Kruppel-like factor 2 (lung) (predicted) | 10,38 | 2,36 |
| 1390943_at | RGD1359529 | similar to chromosome 1 open reading frame 63 | 16,40 | 2,34 |
| 1392791_at | Egr3 | Early growth response 3 | 24,87 | 2,33 |
| 1369640_at | Gja1 | gap junction membrane channel protein alpha 1 | 18,06 | 2,32 |
| 1381864_at | --- | --- | 13,96 | 2,32 |
| 1382478_at | Btbd3_predicted | BTB (POZ) domain containing 3 (predicted) | 15,52 | 2,30 |
| 1386889_at | Scd2 | stearoyl-Coenzyme A desaturase 2 | 19,64 | 2,29 |
| 1373179_at | LOC689994 /// RGD1306921_predicted | similar to mKIAA1107 protein (predicted) /// similar to H43E16.1 | 21,97 | 2,25 |
| 1387662_at | Syt4 | synaptotagmin IV | 17,68 | 2,25 |
| 1380533_at | App | amyloid beta (A4) precursor protein | 17,66 | 2,24 |
| 1398497_at | --- | Transcribed locus | 17,53 | 2,24 |
| 1371019_at | Trib1 | tribbles homolog 1 (Drosophila) | 15,68 | 2,24 |
| 1390060_at | RGD1309360 | Hypothetical LOC294715 | 16,04 | 2,23 |
| 1391668_at | --- | --- | 17,59 | 2,22 |
| 1387260_at | Klf4 | Kruppel-like factor 4 (gut) | 19,76 | 2,22 |
| 1387737_at | Mat2a | methionine adenosyltransferase II, alpha | 19,14 | 2,22 |
| 1396545_at | --- | Transcribed locus | 14,04 | 2,22 |
| 1368662_at | Rnf39 | ring finger protein 39 | 22,34 | 2,21 |
| 1383322_at | Rasl11b | RAS-like family 11 member B | 20,95 | 2,20 |
| 1393237_at | --- | --- | 19,48 | 2,18 |
| 1387707_at | Slc2a3 | solute carrier family 2 (facilitated glucose transporter), member 3 | 13,53 | 2,15 |
| 1384331_at | Srxn1 | Sulfiredoxin 1 homolog (S. cerevisiae) | 14,89 | 2,13 |
| 1376275_at | --- | Transcribed locus | 22,66 | 2,12 |
| 1397107_at | RGD1559968_predicted | Similar to ADP-ribosylation factor guanine nucleotide factor 6 isoform a (predicted) | 14,63 | 2,12 |
| 1371091_at | Irs2 | insulin receptor substrate 2 | 21,39 | 2,11 |
| 1368678_at | Bdnf | brain derived neurotrophic factor | 18,67 | 2,11 |
| 1370760_a_at | Gad1 | glutamic acid decarboxylase 1 | 13,69 | 2,10 |
| 1368506_at | Rgs4 | regulator of G-protein signaling 4 | 20,93 | 2,09 |
| 1369609_at | Cldn11 | claudin 11 | 16,01 | 2,09 |
| 1370174_at | Myd116 | myeloid differentiation primary response gene 116 | 20,70 | 2,07 |
| 1375123_at | Sox4_predicted | SRY-box containing gene 4 (predicted) | 16,57 | 2,06 |
| 1381024_at | Ppp2r1a | protein phosphatase 2 (formerly 2A), regulatory subunit A (PR 65), alpha isoform | 19,64 | 2,05 |
| 1388202_at | RT1-Aw2 | RT1 class Ib, locus Aw2 | 14,92 | 2,05 |
| 1381923_at | RGD1564664_predicted | similar to LOC387763 protein (predicted) | 18,47 | 2,05 |
| 1373559_at | --- | --- | 22,83 | 2,03 |
| 1396820_at | Hdac1_predicted | histone deacetylase 1 (predicted) | 14,98 | 2,03 |
| 1378998_at | --- | Transcribed locus | 19,22 | 2,03 |
| 1387848_at | Hmgcr | 3-hydroxy-3-methylglutaryl-Coenzyme A reductase | 17,61 | 2,03 |
| 1369129_at | Rasgrp1 | RAS guanyl releasing protein 1 | 15,35 | 2,02 |
| 1369152_at | Ppp3r1 | protein phosphatase 3, regulatory subunit B, alpha isoform (calcineurin B, type I) | 17,71 | 2,01 |
| 1375043_at | Fos | FBJ murine osteosarcoma viral oncogene homolog | 27,55 | 2,01 |
| 1392437_at | Alg2 | asparagine-linked glycosylation 2 homolog (yeast, alpha-1,3-mannosyltransferase) | 12,07 | 2,00 |
| 1387630_at | Elovl5 | ELOVL family member 5, elongation of long chain fatty acids (yeast) | 16,09 | 2,00 |
| 1387655_at | Cxcl12 | chemokine (C-X-C motif) ligand 12 | 13,51 | 2,00 |
| 1370131_at | Cav | caveolin | 14,69 | 1,99 |
| 1395561_at | Hnrpa2b1_predicted | heterogeneous nuclear ribonucleoprotein A2/B1 (predicted) | 18,34 | 1,99 |
| 1382497_at | --- | Transcribed locus | 18,54 | 1,98 |
| 1374446_at | Tiparp_predicted | TCDD-inducible poly(ADP-ribose) polymerase (predicted) | 23,56 | 1,98 |
| 1385568_at | Dio2 | Deiodinase, iodothyronine, type II | 14,09 | 1,98 |
| 1369297_at | Ppp2r2c | protein phosphatase 2 (formerly 2A), regulatory subunit B (PR 52), gamma isoform | 11,58 | 1,98 |
| 1370488_a_at | Ptprd | protein tyrosine phosphatase, receptor type, D | 14,63 | 1,98 |
| 1387024_at | Dusp6 | dual specificity phosphatase 6 | 23,19 | 1,97 |
| 1368813_at | Cebpd | CCAAT/enhancer binding protein (C/EBP), delta | 19,27 | 1,97 |
| 1370746_at | Prkacb | protein kinase, cAMP dependent, catalytic, beta | 12,22 | 1,96 |
| 1367633_at | Glul | glutamate-ammonia ligase (glutamine synthase) | 21,36 | 1,96 |
| 1375795_at | --- | Transcribed locus | 15,88 | 1,96 |
| 1369083_at | Cirbp | cold inducible RNA binding protein | 16,46 | 1,96 |
| 1374404_at | Jun | Jun oncogene | 18,99 | 1,96 |
| 1380840_at | Fgfr1 | Fibroblast growth factor receptor 1 | 11,46 | 1,96 |
| 1381798_at | LMO7 | LIM domain only protein 7 | 19,81 | 1,95 |
| 1369597_at | Vapb | vesicle-associated membrane protein, associated protein B and C | 16,02 | 1,95 |
| 1367601_at | Cited2 | Cbp/p300-interacting transactivator, with Glu/Asp-rich carboxy-terminal domain, 2 | 22,70 | 1,95 |
| 1369067_at | Nr4a3 | nuclear receptor subfamily 4, group A, member 3 | 25,13 | 1,95 |
| 1368750_a_at | Pde4d | phosphodiesterase 4D, cAMP specific | 13,73 | 1,94 |
| 1393550_at | Crem | CAMP responsive element modulator | 17,02 | 1,94 |
| 1397516_at | Alg2 | asparagine-linked glycosylation 2 homolog (yeast, alpha-1,3-mannosyltransferase) | 14,57 | 1,94 |
| 1382809_at | Cirbp | cold inducible RNA binding protein | 18,63 | 1,93 |
| 1395340_at | LOC498358 | similar to solute carrier family 30 (zinc transporter), member 9 | 16,16 | 1,93 |
| 1395343_at | RGD1564625_predicted | similar to transmembrane protein TM9SF3 (predicted) | 12,16 | 1,93 |
| 1375130_at | LOC679612 /// LOC687408 /// Zfp216_predicted | zinc finger protein 216 (predicted) /// hypothetical protein LOC679612 /// hypothetical protein LOC687408 | 17,40 | 1,92 |
| 1391759_at | Sh3glb1 | SH3-domain GRB2-like B1 (endophilin) | 15,55 | 1,91 |
| 1392026_at | --- | Transcribed locus | 16,78 | 1,91 |
| 1380381_at | Alg2 | asparagine-linked glycosylation 2 homolog (yeast, alpha-1,3-mannosyltransferase) | 13,35 | 1,91 |
| 1376137_at | Plekhb2_predicted | pleckstrin homology domain containing, family B (evectins) member 2 (predicted) | 15,62 | 1,91 |
| 1382524_at | Zbtb20_predicted | zinc finger and BTB domain containing 20 (predicted) | 14,39 | 1,90 |
| 1385073_at | --- | --- | 18,26 | 1,90 |
| 1369268_at | Atf3 | activating transcription factor 3 | 23,43 | 1,90 |
| 1379687_at | --- | --- | 18,96 | 1,90 |
| 1395173_at | Gpiap1 | GPI-anchored membrane protein 1 | 14,08 | 1,90 |
| 1369718_at | Ssr3 | signal sequence receptor, gamma | 17,45 | 1,89 |
| 1391793_at | --- | Transcribed locus | 11,78 | 1,89 |
| 1369614_at | Rap2b | RAP2B, member of RAS oncogene family | 11,64 | 1,88 |
| 1396409_at | Dtnb | dystrobrevin, beta | 11,76 | 1,88 |
| 1367851_at | Ptgds | prostaglandin D2 synthase | 24,27 | 1,87 |
| 1368577_at | Gjb6 | gap junction membrane channel protein beta 6 | 16,37 | 1,87 |
| 1398175_at | Ppfia2_predicted | Protein tyrosine phosphatase, receptor type, f polypeptide (PTPRF), interacting protein (liprin), alpha 2 (predicted) | 15,70 | 1,87 |
| 1385044_at | RGD1306214_predicted | similar to TGF-beta induced apotosis protein 2 (predicted) | 18,17 | 1,87 |
| 1369291_at | Agtr1a | angiotensin II receptor, type 1 (AT1A) | 12,24 | 1,86 |
| 1368050_at | Ccnl1 | cyclin L1 | 23,48 | 1,85 |
| 1395533_at | Dnd1 | dead end homolog 1 (zebrafish) | 16,07 | 1,85 |
| 1372016_at | Gadd45b | growth arrest and DNA-damage-inducible 45 beta | 20,02 | 1,84 |
| 1382850_at | Syn2 | synapsin II | 18,75 | 1,83 |
| 1369256_at | Bace1 | beta-site APP cleaving enzyme 1 | 13,86 | 1,83 |
| 1376856_at | RGD1310414 | similar to hypothetical protein FLJ23263 | 11,57 | 1,83 |
| 1387882_at | Klf9 | Kruppel-like factor 9 | 16,46 | 1,83 |
| 1370216_at | Ddr1 | discoidin domain receptor family, member 1 | 16,74 | 1,83 |
| 1395586_at | --- | Transcribed locus | 17,28 | 1,82 |
| 1378460_at | RGD1309360 | Hypothetical LOC294715 | 9,37 | 1,82 |
| 1390532_at | --- | Transcribed locus | 17,80 | 1,82 |
| 1388203_x_at | RT1-149 /// RT1-A3 /// RT1-Aw2 /// RT1-CE10 /// RT1-CE15 /// RT1-CE2 /// RT1-T24-1 | RT1 class Ib, locus Aw2 /// RT1 class I, A3 /// histocompatibility 2, T region locus 24 /// RT1 class I, CE2 /// RT1-149 protein... <Preview truncated at 128 characters> | 15,48 | 1,82 |
| 1396541_at | Jmjd1c | jumonji domain containing 1C | 10,20 | 1,82 |
| 1378593_at | --- | Transcribed locus | 16,33 | 1,82 |
| 1368808_at | Cap1 | CAP, adenylate cyclase-associated protein 1 (yeast) | 19,42 | 1,81 |
| 1367795_at | Ifrd1 | interferon-related developmental regulator 1 | 24,73 | 1,81 |
| 1387420_at | Clic4 | chloride intracellular channel 4 | 13,33 | 1,81 |
| 1368984_at | Sept2 | septin 2 | 15,69 | 1,81 |
| 1392758_at | RGD1562563_predicted | similar to RIKEN cDNA G430041M01 (predicted) | 15,12 | 1,80 |
| 1394931_at | LOC686892 | similar to muscleblind-like 1 isoform d | 16,39 | 1,80 |
| 1378182_at | --- | Transcribed locus | 15,82 | 1,80 |
| 1383485_at | Mdm2_predicted | transformed mouse 3T3 cell double minute 2 homolog (mouse) (predicted) | 14,30 | 1,79 |
| 1372375_at | Atg16l1_predicted | ATG16 autophagy related 16-like 1 (S. cerevisiae) (predicted) | 21,84 | 1,79 |
| 1390881_at | Abra | actin-binding Rho activating protein | 13,39 | 1,79 |
| 1369885_at | --- | --- | 15,13 | 1,78 |
| 1395575_at | --- | --- | 13,86 | 1,78 |
| 1389831_at | Arih1 | Ariadne ubiquitin-conjugating enzyme E2 binding protein homolog 1 (Drosophila) | 14,58 | 1,77 |
| 1371108_a_at | Atp1a1 | ATPase, Na+/K+ transporting, alpha 1 polypeptide | 19,76 | 1,77 |
| 1395695_at | Aebp1_predicted | AE binding protein 1 (predicted) | 11,98 | 1,77 |
| 1370556_at | Vamp1 | vesicle-associated membrane protein 1 | 17,56 | 1,77 |
| 1374855_at | Per1 | period homolog 1 (Drosophila) | 21,61 | 1,76 |
| 1386961_at | Pfkm | phosphofructokinase, muscle | 19,52 | 1,76 |
| 1379515_at | Pigt_predicted | phosphatidylinositol glycan, class T (predicted) | 16,24 | 1,76 |
| 1370773_a_at | Kcnip2 | Kv channel-interacting protein 2 | 14,71 | 1,76 |
| 1392785_at | Kctd12_predicted | Potassium channel tetramerisation domain containing 12 (predicted) | 18,54 | 1,76 |
| 1381646_at | --- | --- | 17,11 | 1,76 |
| 1370792_at | Mapre1 | microtubule-associated protein, RP/EB family, member 1 | 18,39 | 1,75 |
| 1379088_x_at | Mgat1 | Mannoside acetylglucosaminyltransferase 1 | 10,45 | 1,75 |
| 1387341_a_at | Mbp | myelin basic protein | 21,56 | 1,75 |
| 1389837_at | Rnf187_predicted | ring finger protein 187 (predicted) | 18,82 | 1,74 |
| 1396939_at | Pcsk5 | Proprotein convertase subtilisin/kexin type 5 | 14,55 | 1,74 |
| 1385423_at | Immt | inner membrane protein, mitochondrial | 12,94 | 1,74 |
| 1382128_at | --- | --- | 21,28 | 1,74 |
| 1394077_at | Rnd3 | Rho family GTPase 3 | 18,25 | 1,74 |
| 1369704_at | Xtrp3 | X transporter protein 3 | 17,28 | 1,73 |
| 1393657_at | Prcp_predicted | prolylcarboxypeptidase (angiotensinase C) (predicted) | 15,91 | 1,73 |
| 1387372_at | Slc6a13 | solute carrier family 6 (neurotransmitter transporter, GABA), member 13 | 16,10 | 1,73 |
| 1396024_at | Sptlc1_predicted | serine palmitoyltransferase, long chain base subunit 1 (predicted) | 13,36 | 1,73 |
| 1394405_at | RGD1310139_predicted | Similar to KIAA0303 (predicted) | 14,34 | 1,73 |
| 1384716_at | Syt1 | synaptotagmin I | 16,45 | 1,73 |
| 1394427_at | RGD1311678 | Similar to 4921517L17Rik protein | 19,14 | 1,73 |
| 1397492_at | LMO7 | LIM domain only protein 7 | 14,15 | 1,73 |
| 1369689_at | Nsf | N-ethylmaleimide sensitive fusion protein | 19,79 | 1,72 |
| 1368948_at | Msn | moesin | 14,13 | 1,72 |
| 1384608_at | Opa1 | Optic atrophy 1 homolog (human) | 16,48 | 1,72 |
| AFFX_Rat_beta-actin_5_at | --- | --- | 23,21 | 1,72 |
| 1369627_at | Sv2b | synaptic vesicle glycoprotein 2b | 18,25 | 1,72 |
| 1375475_at | Dusp5 | Dual specificity phosphatase 5 | 13,71 | 1,72 |
| 1368704_a_at | Cspg5 | chondroitin sulfate proteoglycan 5 | 18,46 | 1,72 |
| 1395886_at | Actr3 | ARP3 actin-related protein 3 homolog (yeast) | 17,24 | 1,71 |
| 1396462_at | Vps4a | vacuolar protein sorting 4a (yeast) | 14,95 | 1,71 |
| 1389874_at | LOC498425 | similar to U2 small nuclear ribonucleoprotein auxiliary factor 35 kDa subunit related-protein 1 (U2(RNU2) small nuclear RNA auxi... <Preview truncated at 128 characters> | 15,14 | 1,71 |
| 1377580_at | RGD1565474_predicted | similar to mKIAA0738 protein (predicted) | 12,81 | 1,71 |
| 1385270_s_at | LOC682488 /// MGC105830 | similar to Ras-related protein Rab-1B | 14,66 | 1,71 |
| 1382832_at | Rcc2_predicted | regulator of chromosome condensation 2 (predicted) | 15,17 | 1,71 |
| 1371687_at | Canx | calnexin | 18,67 | 1,71 |
| 1393606_at | RGD735140 | hypothetical protein LK44 | 12,89 | 1,71 |
| 1382616_at | Gls | glutaminase | 14,13 | 1,70 |
| 1396742_at | --- | --- | 10,88 | 1,70 |
| 1368596_at | Snf1lk | SNF1-like kinase | 19,38 | 1,70 |
| 1370728_at | Il13ra1 | interleukin 13 receptor, alpha 1 | 12,72 | 1,70 |
| 1387512_at | Zfp238 | zinc finger protein 238 | 16,46 | 1,70 |
| 1369560_at | Gpd1 | glycerol-3-phosphate dehydrogenase 1 (soluble) | 13,97 | 1,70 |
| 1385016_at | RGD1560784_predicted | similar to RIKEN cDNA B630019K06 (predicted) | 20,59 | 1,70 |
| 1371731_at | Mest | Mesoderm specific transcript | 19,21 | 1,70 |
| 1386762_at | Snag1_predicted | sorting nexin associated golgi protein 1 (predicted) | 14,27 | 1,70 |
| 1397130_at | Mapk10 | Mitogen activated protein kinase 10 | 15,94 | 1,70 |
| 1397675_at | Wbscr1 | Williams-Beuren syndrome chromosome region 1 homolog (human) | 15,48 | 1,69 |
| 1385691_at | RGD1560454_predicted | similar to RIKEN cDNA 5830435K17 (predicted) | 15,18 | 1,69 |
| 1388157_at | Marcks | myristoylated alanine rich protein kinase C substrate | 16,90 | 1,69 |
| 1385658_at | Zfp313 | zinc finger protein 313 | 15,53 | 1,69 |
| 1387374_at | Tcf12 | transcription factor 12 | 12,73 | 1,69 |
| 1370116_at | Sept3 | septin 3 | 15,30 | 1,69 |
| 1375177_at | RGD1565099_predicted | similar to BTEB3 protein (predicted) | 16,20 | 1,69 |
| 1370599_a_at | Ptprd | protein tyrosine phosphatase, receptor type, D | 19,34 | 1,68 |
| 1383072_at | Pygm | muscle glycogen phosphorylase | 12,01 | 1,68 |
| 1382561_at | --- | --- | 13,10 | 1,68 |
| 1374888_at | Ccdc49_predicted | coiled-coil domain containing 49 (predicted) | 21,72 | 1,68 |
| 1383486_at | --- | Transcribed locus | 21,28 | 1,68 |
| 1394078_at | --- | --- | 19,61 | 1,68 |
| 1387388_at | Chp /// RGD1564956_predicted /// RGD1565588_predicted | calcium binding protein p22 /// similar to calcium binding protein P22 (predicted) | 16,06 | 1,68 |
| 1395394_at | Gga2 | golgi associated, gamma adaptin ear containing, ARF binding protein 2 | 12,09 | 1,68 |
| 1397460_at | LOC686523 | Similar to G protein-coupled receptor 158 isoform a | 15,60 | 1,67 |
| 1381746_at | Elmo1_predicted | Engulfment and cell motility 1, ced-12 homolog (C. elegans) (predicted) | 16,79 | 1,67 |
| 1369520_a_at | Bcat1 | branched chain aminotransferase 1, cytosolic | 21,30 | 1,67 |
| 1390726_at | Bcat1 | Branched chain aminotransferase 1, cytosolic | 17,50 | 1,67 |
| 1395273_at | Ahcyl1_predicted | S-adenosylhomocysteine hydrolase-like 1 (predicted) | 16,62 | 1,67 |
| 1389528_s_at | Jun | Jun oncogene | 23,45 | 1,67 |
| 1368106_at | Plk2 | polo-like kinase 2 (Drosophila) | 25,68 | 1,67 |
| 1380331_at | RGD1564695_predicted | similar to A830059I20Rik protein (predicted) | 12,66 | 1,67 |
| 1393119_at | --- | Transcribed locus | 23,14 | 1,67 |
| 1375335_at | Hspcb | heat shock 90kDa protein 1, beta | 24,30 | 1,67 |
| 1378393_at | RGD1306214_predicted | similar to TGF-beta induced apotosis protein 2 (predicted) | 14,85 | 1,67 |
| 1375193_at | Lrp11_predicted | low density lipoprotein receptor-related protein 11 (predicted) | 13,40 | 1,66 |
| 1368030_at | Gnai3 | guanine nucleotide binding protein, alpha inhibiting 3 | 15,41 | 1,66 |
| 1395100_at | RGD1565549_predicted | similar to polybromo-1 (predicted) | 15,16 | 1,66 |
| 1382942_at | LOC299907 | Similar to Ext1 | 16,55 | 1,66 |
| 1367823_at | Timp2 | tissue inhibitor of metalloproteinase 2 | 18,08 | 1,66 |
| 1395370_at | Gmcl1 | germ cell-less homolog 1 (Drosophila) | 13,15 | 1,66 |
| 1375465_at | Otx2 | orthodenticle homolog 2 (Drosophila) | 13,49 | 1,66 |
| 1379706_at | --- | Transcribed locus | 13,80 | 1,66 |
| 1367602_at | Cited2 | Cbp/p300-interacting transactivator, with Glu/Asp-rich carboxy-terminal domain, 2 | 24,39 | 1,66 |
| 1398664_at | Gramd3 | GRAM domain containing 3 | 19,27 | 1,66 |
| 1369371_a_at | Gabbr1 | gamma-aminobutyric acid (GABA) B receptor 1 | 17,75 | 1,66 |
| 1397522_at | Sbf1_predicted | SET binding factor 1 (predicted) | 15,76 | 1,66 |
| 1369571_at | Golph3 | golgi phosphoprotein 3 | 18,30 | 1,66 |
| 1377445_at | Cdh22 | Cadherin 22 | 15,27 | 1,65 |
| 1387908_at | Rasd1 | RAS, dexamethasone-induced 1 | 19,39 | 1,65 |
| 1390923_a_at | Osbpl1a | oxysterol binding protein-like 1A | 16,58 | 1,65 |
| 1389402_at | --- | Transcribed locus | 19,27 | 1,65 |
| 1371090_at | Scamp2 | secretory carrier membrane protein 2 | 15,99 | 1,65 |
| 1367959_a_at | Scn1b | sodium channel, voltage-gated, type I, beta | 18,46 | 1,65 |
| 1395346_at | Aamp_predicted | angio-associated migratory protein (predicted) | 18,26 | 1,65 |
| 1375885_at | Mtmr1_predicted | myotubularin related protein 1 (predicted) | 12,13 | 1,65 |
| 1398538_at | Abhd8_predicted | abhydrolase domain containing 8 (predicted) | 15,81 | 1,65 |
| 1373093_at | Errfi1 | ERBB receptor feedback inhibitor 1 | 24,56 | 1,65 |
| 1373866_at | RGD1359509 | similar to hypothetical protein FLJ13448 | 24,34 | 1,65 |
| 1387359_at | Stx1a | syntaxin 1A (brain) | 15,16 | 1,64 |
| 1373975_at | LOC368066 | similar to indolethylamine N-methyltransferase | 18,96 | 1,64 |
| 1398476_at | Vcl_predicted | vinculin (predicted) | 17,33 | 1,64 |
| 1389538_at | Nfkbia | nuclear factor of kappa light chain gene enhancer in B-cells inhibitor, alpha | 21,37 | 1,64 |
| 1369733_at | Ctnnb1 | catenin (cadherin associated protein), beta 1 | 20,84 | 1,64 |
| 1397856_at | Pet112l_predicted | PET112-like (yeast) (predicted) | 11,32 | 1,64 |
| 1369234_at | Slc20a2 | solute carrier family 20, member 2 | 15,13 | 1,64 |
| 1392354_at | Prkg1 | Protein kinase, cGMP-dependent, type 1 (mapped) | 11,11 | 1,64 |
| 1398037_at | --- | Transcribed locus | 14,55 | 1,63 |
| 1397231_at | Sprn | Shadow of prion protein | 14,89 | 1,63 |
| 1368505_at | Rgs4 | regulator of G-protein signaling 4 | 25,10 | 1,63 |
| 1390070_at | --- | --- | 17,56 | 1,63 |
| 1380373_at | Gmps | guanine monphosphate synthetase | 13,26 | 1,63 |
| 1369568_at | Stx6 | syntaxin 6 | 13,97 | 1,63 |
| 1385455_at | LOC499691 | similar to sarcoma antigen NY-SAR-27 | 11,18 | 1,63 |
| 1380816_at | RGD1563235_predicted | similar to 1700054N08Rik protein (predicted) | 16,91 | 1,63 |
| 1395236_at | Ppp1r3c | protein phosphatase 1, regulatory (inhibitor) subunit 3C | 15,24 | 1,62 |
| 1384975_at | Fxna | putative aminopeptidase Fxna | 15,17 | 1,62 |
| 1382040_at | Eprs | glutamyl-prolyl-tRNA synthetase | 12,78 | 1,62 |
| 1370955_at | Adam10 | a disintegrin and metalloprotease domain 10 | 11,40 | 1,62 |
| 1381008_at | --- | Transcribed locus | 17,69 | 1,62 |
| 1380363_at | Klf7_predicted | Kruppel-like factor 7 (ubiquitous) (predicted) | 14,42 | 1,62 |
| 1369278_at | Gna12 | guanine nucleotide binding protein, alpha 12 | 14,90 | 1,62 |
| 1388064_a_at | Slc1a3 | solute carrier family 1 (glial high affinity glutamate transporter), member 3 | 19,20 | 1,62 |
| 1390049_at | Fhl1 | four and a half LIM domains 1 | 17,96 | 1,62 |
| 1395157_at | Nedd4a | neural precursor cell expressed, developmentally down-regulated gene 4A | 17,24 | 1,61 |
| 1388686_at | Dscr1 | Down syndrome critical region homolog 1 (human) | 21,99 | 1,61 |
| 1392381_at | --- | --- | 16,33 | 1,61 |
| 1387870_at | Zfp36 | zinc finger protein 36 | 19,34 | 1,61 |
| 1387282_at | Hspb8 | heat shock 22kDa protein 8 | 17,41 | 1,61 |
| 1397200_at | Chd4 | chromodomain helicase DNA binding protein 4 | 16,68 | 1,61 |
| 1385582_at | Osbpl2 | oxysterol binding protein-like 2 | 11,99 | 1,61 |
| 1371103_at | Rab6a | RAB6A, member RAS oncogene family | 20,22 | 1,61 |
| 1395914_at | --- | --- | 16,38 | 1,61 |
| 1394616_at | Mesdc2 | mesoderm development candiate 2 | 15,35 | 1,61 |
| 1375486_at | LOC679039 /// LOC680025 | similar to nuclear RNA export factor 2 | 17,31 | 1,61 |
| 1378540_at | LOC678970 | hypothetical protein LOC678970 | 20,72 | 1,61 |
| 1397960_at | LOC367902 | similar to ALEX3 protein | 13,87 | 1,61 |
| 1388125_a_at | Klc1 | kinesin light chain 1 | 18,54 | 1,61 |
| 1370993_at | Lamc1 | laminin, gamma 1 | 16,07 | 1,61 |
| 1375762_at | Dnajb5_predicted | DnaJ (Hsp40) homolog, subfamily B, member 5 (predicted) | 12,91 | 1,61 |
| 1369032_at | Blcap | bladder cancer associated protein homolog (human) | 17,05 | 1,61 |
| 1369427_at | Mpeg1 | macrophage expressed gene 1 | 13,48 | 1,60 |
| 1395228_at | --- | Transcribed locus | 11,45 | 1,60 |
| 1386977_at | Ca3 | carbonic anhydrase 3 | 15,94 | 1,60 |
| 1378587_at | LOC688144 /// LOC690586 | similar to ankyrin repeat domain 40 | 16,16 | 1,60 |
| 1385323_at | --- | Transcribed locus | 16,03 | 1,60 |
| AFFX-DapX-5_at | --- | --- | 17,31 | 1,60 |
| 1370558_a_at | Kcnc2 | potassium voltage gated channel, Shaw-related subfamily, member 2 | 10,37 | 1,60 |
| 1385302_at | Gdpd1_predicted | glycerophosphodiester phosphodiesterase domain containing 1 (predicted) | 14,46 | 1,60 |
| 1377765_at | Clic4 | chloride intracellular channel 4 | 15,02 | 1,60 |
| 1369778_at | Dio2 | deiodinase, iodothyronine, type II | 14,53 | 1,59 |
| AFFX_rat_5S_rRNA_at | --- | --- | 17,26 | 1,59 |
| 1387316_at | Cxcl1 | chemokine (C-X-C motif) ligand 1 | 20,44 | 1,59 |
| 1389754_at | --- | Transcribed locus, weakly similar to XP_529632.1 hypothetical protein XP_529632 [Pan troglodytes] | 21,64 | 1,59 |
| 1398825_at | Rab11b | RAB11B, member RAS oncogene family | 19,90 | 1,59 |
| 1371209_at | RT1-CE5 | RT1 class I, CE5 | 14,67 | 1,59 |
| 1371038_at | Cebpg | CCAAT/enhancer binding protein (C/EBP), gamma | 14,41 | 1,59 |
| 1369651_at | Thy1 | thymus cell antigen 1, theta | 19,78 | 1,59 |
| 1370668_a_at | Cnksr2 | connector enhancer of kinase suppressor of Ras 2 | 15,12 | 1,59 |
| 1383306_at | RGD1560454_predicted | similar to RIKEN cDNA 5830435K17 (predicted) | 23,40 | 1,59 |
| 1370123_a_at | Cttn | cortactin | 15,28 | 1,58 |
| 1380695_at | --- | Transcribed locus | 18,57 | 1,58 |
| 1395572_at | Dnaja4 | DnaJ (Hsp40) homolog, subfamily A, member 4 | 12,49 | 1,58 |
| 1383813_at | Arl2bp | ADP-ribosylation factor-like 2 binding protein | 19,46 | 1,58 |
| 1367571_a_at | Igf2 | insulin-like growth factor 2 | 20,84 | 1,58 |
| 1370540_at | Nr1d2 | nuclear receptor subfamily 1, group D, member 2 | 10,99 | 1,58 |
| 1396154_at | --- | --- | 13,36 | 1,58 |
| 1368986_at | Slc17a7 | solute carrier family 17 (sodium-dependent inorganic phosphate cotransporter), member 7 | 18,95 | 1,58 |
| 1393151_at | Etnk1_predicted | ethanolamine kinase 1 (predicted) | 18,25 | 1,58 |
| 1370210_at | Ris1 | Ras-induced senescence 1 | 15,51 | 1,58 |
| 1375153_at | RGD1565646_predicted | similar to SOX2 protein (predicted) | 15,77 | 1,58 |
| 1395512_at | Crlf1_predicted | cytokine receptor-like factor 1 (predicted) | 16,19 | 1,58 |
| 1387408_at | Siah2 | seven in absentia 2 | 18,98 | 1,58 |
| 1388082_at | Dusp4 | dual specificity phosphatase 4 | 13,95 | 1,58 |
| 1381565_at | --- | Transcribed locus | 11,77 | 1,58 |
| 1382976_at | Polr2a | Polymerase (RNA) II (DNA directed) polypeptide A (mapped) | 13,56 | 1,58 |
| 1368003_at | Aldh1a2 | aldehyde dehydrogenase family 1, subfamily A2 | 19,02 | 1,58 |
| 1387969_at | Cxcl10 | chemokine (C-X-C motif) ligand 10 | 15,32 | 1,58 |
| 1397469_at | Dst_predicted | dystonin (predicted) | 12,66 | 1,58 |
| 1387844_at | Lasp1 | LIM and SH3 protein 1 | 13,91 | 1,57 |
| 1397526_at | Gcdh_predicted | glutaryl-Coenzyme A dehydrogenase (predicted) | 15,70 | 1,57 |
| 1380878_at | Mapk10 | Mitogen activated protein kinase 10 | 16,09 | 1,57 |
| 1395338_at | Lrpprc | leucine-rich PPR-motif containing | 12,41 | 1,57 |
| 1369526_at | Acadsb | acyl-Coenzyme A dehydrogenase, short/branched chain | 11,16 | 1,57 |
| 1368046_at | Slc31a1 | solute carrier family 31 (copper transporters), member 1 | 14,19 | 1,57 |
| 1376499_at | Centg3_predicted | centaurin, gamma 3 (predicted) | 14,54 | 1,57 |
| 1377006_at | Cct6a | Chaperonin subunit 6a (zeta) | 18,18 | 1,57 |
| 1380808_at | Tollip_predicted | toll interacting protein (predicted) | 15,64 | 1,57 |
| 1382287_at | Rnps1 | ribonucleic acid binding protein S1 | 17,30 | 1,57 |
| 1369705_at | Xtrp3 | X transporter protein 3 | 21,14 | 1,57 |
| 1368964_at | Lrrn3 | leucine rich repeat protein 3, neuronal | 23,16 | 1,57 |
| 1392581_at | Ncald | neurocalcin delta | 18,58 | 1,57 |
| 1369130_at | Rasgrp1 | RAS guanyl releasing protein 1 | 17,13 | 1,57 |
| 1370758_at | Rab15 | RAB15, member RAS onocogene family | 16,11 | 1,57 |
| 1376963_at | Dyrk2_predicted | dual-specificity tyrosine-(Y)-phosphorylation regulated kinase 2 (predicted) | 17,53 | 1,57 |
| 1375696_at | Ifnar1_predicted | interferon (alpha and beta) receptor 1 (predicted) | 15,09 | 1,57 |
| AFFX-r2-Bs-thr-5_s_at | --- | --- | 15,15 | 1,57 |
| 1391938_at | Usp11 | ubiquitin specific protease 11 | 14,12 | 1,56 |
| 1382443_at | Pabpc4 /// RGD1562451_predicted | similar to Pabpc4_predicted protein (predicted) /// poly A binding protein, cytoplasmic 4 | 14,16 | 1,56 |
| 1391491_a_at | Rad23b | RAD23b homolog (S. cerevisiae) | 14,60 | 1,56 |
| 1383037_at | Poldip2_predicted | polymerase (DNA-directed), delta interacting protein 2 (predicted) | 18,02 | 1,56 |
| 1376008_at | Cbx8 | chromobox homolog 8 (Drosophila, Pc class) | 15,29 | 1,56 |
| 1374129_at | LOC690789 /// RGD1562933_predicted | similar to product is unknown~seizure-related gene (predicted) /// similar to Ornithine decarboxylase antizyme 2 (ODC-Az 2) (AZ2... <Preview truncated at 128 characters> | 19,82 | 1,56 |
| 1386716_at | --- | CDNA clone IMAGE:7320582 | 15,16 | 1,56 |
| AFFX-r2-Bs-dap-5_at | --- | --- | 18,33 | 1,56 |
| 1391771_at | Atp2a2 | ATPase, Ca++ transporting, cardiac muscle, slow twitch 2 | 16,40 | 1,56 |
| 1370662_a_at | Ap2b1 | adaptor-related protein complex 2, beta 1 subunit | 16,78 | 1,56 |
| 1369924_at | Sncb | synuclein, beta | 18,67 | 1,56 |
| 1387095_at | Gnaz | guanine nucleotide binding protein, alpha z subunit | 14,48 | 1,56 |
| 1395855_at | Arih2_predicted | ariadne homolog 2 (Drosophila) (predicted) | 13,88 | 1,56 |
| AFFX-r2-Bs-phe-5_at | --- | --- | 14,94 | 1,56 |
| 1383759_at | Agtpbp1_predicted | ATP/GTP binding protein 1 (predicted) | 15,76 | 1,56 |
| 1397512_at | RGD1560511_predicted | similar to Vps41 protein (predicted) | 17,70 | 1,56 |
| 1394668_at | [] | [] | 15,65 | 1,56 |
| 1395640_at | Cdc23 | CDC23 (cell division cycle 23, yeast, homolog) | 13,33 | 1,56 |
| 1397693_at | Eif2s3x | eukaryotic translation initiation factor 2, subunit 3, structural gene X-linked | 17,38 | 1,56 |
| 1393324_at | Jam2 | junction adhesion molecule 2 | 16,45 | 1,55 |
| 1388030_a_at | Gabbr1 | gamma-aminobutyric acid (GABA) B receptor 1 | 17,05 | 1,55 |
| 1370017_at | Emd | emerin | 20,47 | 1,55 |
| 1385011_at | Smarca4 | SWI/SNF related, matrix associated, actin dependent regulator of chromatin, subfamily a, member 4 | 15,22 | 1,55 |
| 1367948_a_at | Kdr | kinase insert domain protein receptor | 16,29 | 1,55 |
| 1398311_a_at | Kidins220 | kinase D-interacting substance 220 | 15,00 | 1,55 |
| 1387657_at | Kif3c | kinesin family member 3C | 16,80 | 1,55 |
| 1389851_at | Zfp36l2 | zinc finger protein 36, C3H type-like 2 | 11,51 | 1,55 |
| 1396464_at | Sh3gl2 | SH3-domain GRB2-like 2 | 11,55 | 1,55 |
| 1367725_at | Pim3 | serine/threonine-protein kinase pim-3 | 19,02 | 1,55 |
| 1386909_a_at | Vdac1 | voltage-dependent anion channel 1 | 21,14 | 1,55 |
| 1387541_at | Cspg3 | chondroitin sulfate proteoglycan 3 | 15,69 | 1,55 |
| 1371754_at | Slc25a25 | solute carrier family 25 (mitochondrial carrier, phosphate carrier), member 25 | 21,30 | 1,55 |
| 1383489_at | Il6st | Interleukin 6 signal transducer | 16,85 | 1,55 |
| 1368025_at | Ddit4 | DNA-damage-inducible transcript 4 | 21,60 | 1,55 |
| 1382631_at | LOC685277 | Similar to liver-specific bHLH-Zip transcription factor | 15,37 | 1,55 |
| 1376694_at | --- | --- | 15,85 | 1,54 |
| 1393166_at | --- | --- | 19,24 | 1,54 |
| 1369452_a_at | Picalm | phosphatidylinositol binding clathrin assembly protein | 17,45 | 1,54 |
| 1392024_at | --- | Transcribed locus | 15,31 | 1,54 |
| 1395274_at | Dst_predicted | dystonin (predicted) | 14,57 | 1,54 |
| 1375117_at | Akt1s1_predicted | AKT1 substrate 1 (proline-rich) (predicted) | 14,54 | 1,54 |
| 1379219_at | --- | --- | 13,31 | 1,54 |
| 1369236_at | Prdm4 | PR domain containing 4 | 13,72 | 1,54 |
| 1369738_s_at | Crem | cAMP responsive element modulator | 18,12 | 1,54 |
| 1397988_at | RGD1563580_predicted | similar to AP2 associated kinase 1 (predicted) | 13,70 | 1,54 |
| 1389146_at | LOC498796 | hypothetical protein LOC498796 | 21,13 | 1,54 |
| 1384511_at | Dnajb5_predicted | DnaJ (Hsp40) homolog, subfamily B, member 5 (predicted) | 15,87 | 1,54 |
| 1395557_at | --- | Transcribed locus | 17,16 | 1,54 |
| 1387624_at | Usf1 | upstream transcription factor 1 | 17,51 | 1,54 |
| 1368874_a_at | Mafg | v-maf musculoaponeurotic fibrosarcoma oncogene family, protein G (avian) | 14,54 | 1,54 |
| 1396691_at | --- | Transcribed locus | 14,98 | 1,54 |
| 1381668_at | Tspan5 | Tetraspanin 5 | 15,69 | 1,54 |
| 1370584_a_at | Adora1 | adenosine A1 receptor | 12,46 | 1,54 |
| 1389211_at | [] | Similar to protein phosphatase 1, regulatory (inhibitory) subunit 1C | 24,66 | 1,53 |
| 1368989_at | Timp3 | tissue inhibitor of metalloproteinase 3 (Sorsby fundus dystrophy, pseudoinflammatory) | 13,81 | 1,53 |
| 1375754_at | Impact | imprinted and ancient | 13,68 | 1,53 |
| 1382673_at | Nrd1 | Nardilysin, N-arginine dibasic convertase 1 | 17,70 | 1,53 |
| 1373860_at | Sox4_predicted | SRY-box containing gene 4 (predicted) | 21,19 | 1,53 |
| 1389961_at | LOC683605 | similar to serologically defined colon cancer antigen 3 isoform 1 | 13,68 | 1,53 |
| 1369857_a_at | Slc14a1 | solute carrier family 14 (urea transporter), member 1 | 16,33 | 1,53 |
| 1397218_at | Dpysl3 | Dihydropyrimidinase-like 3 | 13,43 | 1,53 |
| 1369583_at | Jundp2 | Jun dimerization protein 2 | 12,75 | 1,53 |
| 1375468_at | --- | --- | 16,28 | 1,53 |
| 1375627_at | RGD1307791_predicted | similar to hypothetical protein FLJ10342 (predicted) | 16,90 | 1,53 |
| 1369537_at | Mchr1 | melanin-concentrating hormone receptor 1 | 14,64 | 1,53 |
| 1376245_x_at | --- | --- | 16,27 | 1,53 |
| 1383023_at | --- | --- | 13,00 | 1,53 |
| 1387447_at | Arf3 | ADP-ribosylation factor 3 | 18,35 | 1,53 |
| 1392883_at | RGD1305269_predicted | similar to hypothetical protein (predicted) | 16,62 | 1,53 |
| 1370428_x_at | RT1-A2 /// RT1-A3 /// RT1-Aw2 | RT1 class Ib, locus Aw2 /// RT1 class Ia, locus A2 /// RT1 class I, A3 | 20,93 | 1,53 |
| 1384728_at | Ches1_predicted | checkpoint suppressor 1 (predicted) | 13,70 | 1,52 |
| 1389882_at | Ier5 | immediate early response 5 | 15,10 | 1,52 |
| 1392639_at | --- | --- | 14,46 | 1,52 |
| 1379554_at | Ppp2r5e_predicted | protein phosphatase 2, regulatory subunit B (B56), epsilon isoform (predicted) | 12,37 | 1,52 |
| 1379724_at | LOC685611 | similar to pleckstrin homology-like domain, family B, member 2 | 13,50 | 1,52 |
| 1370135_at | Cav2 | caveolin 2 | 13,38 | 1,52 |
| 1369650_at | Pak2 | p21 (CDKN1A)-activated kinase 2 | 15,70 | 1,52 |
| 1371060_at | Trim23 | tripartite motif protein 23 | 17,23 | 1,52 |
| 1379591_at | --- | --- | 15,91 | 1,52 |
| 1375973_at | Arih1 | ariadne ubiquitin-conjugating enzyme E2 binding protein homolog 1 (Drosophila) | 17,59 | 1,52 |
| 1371179_a_at | Fgfr2 | fibroblast growth factor receptor 2 | 10,24 | 1,52 |
| 1384240_at | Agtr1a | angiotensin II receptor, type 1 (AT1A) | 14,35 | 1,52 |
| 1369793_a_at | Mcam | melanoma cell adhesion molecule | 12,06 | 1,52 |
| 1396056_at | Dmxl1_predicted | Dmx-like 1 (predicted) | 14,56 | 1,52 |
| AFFX_Rat_Hexokinase_5_at | --- | --- | 14,30 | 1,52 |
| 1369554_at | Syngr2 | synaptogyrin 2 | 16,38 | 1,51 |
| 1383302_at | Dnajb1_predicted | DnaJ (Hsp40) homolog, subfamily B, member 1 (predicted) | 20,32 | 1,51 |
| 1377485_at | Gja12_predicted | gap junction protein, alpha 12, 47kDa (predicted) | 14,27 | 1,51 |
| 1397809_at | Mapk8ip3 | Mitogen-activated protein kinase 8 interacting protein 3 | 16,07 | 1,51 |
| 1391867_at | --- | --- | 16,28 | 1,51 |
| 1383899_at | Nedd4a | neural precursor cell expressed, developmentally down-regulated gene 4A | 20,82 | 1,51 |
| 1385214_at | Ddx3x | DEAD/H (Asp-Glu-Ala-Asp/His) box polypeptide 3, X-linked | 17,94 | 1,51 |
| 1391975_at | --- | Transcribed locus | 18,38 | 1,51 |
| 1391701_at | --- | Transcribed locus | 18,84 | 1,51 |
| 1393591_at | RGD1564996_predicted | similar to tumor necrosis factor receptor superfamily, member 19 (predicted) | 14,36 | 1,51 |
| 1370141_at | --- | --- | 18,24 | 1,51 |
| 1370780_at | Rab31 | RAB31, member RAS oncogene family | 16,45 | 1,51 |
| 1379822_at | RGD1307395 | similar to SR rich protein | 12,58 | 1,50 |
| 1368832_at | Akt2 | thymoma viral proto-oncogene 2 | 15,68 | 1,50 |
| 1391838_at | Ankrd11_predicted | ankyrin repeat domain 11 (predicted) | 13,01 | 1,50 |
| AFFX_Rat_beta-actin_M_at | --- | --- | 25,32 | 1,50 |
| 1393619_at | Cnot6l_predicted | CCR4-NOT transcription complex, subunit 6-like (predicted) | 15,31 | 1,50 |
| 1386106_at | --- | Transcribed locus | 17,00 | 1,50 |
| 1387513_at | Pscd3 | pleckstrin homology, Sec7 and coiled-coil domains 3 | 10,86 | 1,50 |
| 1381542_at | Ubxd2 | UBX domain containing 2 | 17,70 | 1,50 |
| 1377750_at | Arhgef3_predicted | Rho guanine nucleotide exchange factor (GEF) 3 (predicted) | 21,78 | 1,50 |
| 1376952_at | Diras2_predicted | DIRAS family, GTP-binding RAS-like 2 (predicted) | 16,61 | 1,50 |
| 1394315_at | LOC683788 | similar to Fascin (Singed-like protein) | 14,16 | 1,50 |
| 1388842_at | Srf_predicted | serum response factor (predicted) | 22,29 | 1,50 |
| 1367970_at | Pfn2 | profilin 2 | 20,35 | 1,50 |
| 1388299_at | --- | Transcribed locus | 12,52 | 1,50 |
| 1377518_at | Camk1g | calcium/calmodulin-dependent protein kinase I gamma | 13,46 | 1,50 |
| 1369643_a_at | Lphn2 | latrophilin 2 | 13,03 | 1,50 |
| 1387458_at | Rnf4 | ring finger protein 4 | 18,28 | 1,50 |
| 1369319_at | Arl6ip5 | ADP-ribosylation factor-like 6 interacting protein 5 | 22,05 | 1,50 |
| 1392842_at | --- | Transcribed locus | 12,90 | 1,50 |
| 1380220_at | Osbp2_predicted | Oxysterol binding protein 2 (predicted) | 13,23 | 1,49 |
| 1374283_at | Falz_predicted | fetal Alzheimer antigen (predicted) | 16,45 | 1,49 |
| 1388061_a_at | Epha7 | Eph receptor A7 | 17,18 | 1,49 |
| 1367802_at | Sgk | serum/glucocorticoid regulated kinase | 25,33 | 1,49 |
| 1398849_at | H3f3b | H3 histone, family 3B | 24,03 | 1,49 |
| 1384854_at | RGD1566359_predicted | Similar to RIKEN cDNA B230219D22 (predicted) | 15,12 | 1,49 |
| 1369518_at | Pik3r3 | phosphatidylinositol 3 kinase, regulatory subunit, polypeptide 3 | 15,28 | 1,49 |

**Table B**

List of the primer pairs used in the time-course analysis.

***Atf3***

LEFT PRIMER 5'-**CGCCATCCAGAACAAGCAC**-3'

RIGHT PRIMER 5'-**CGGCATTCACACTCTCCAGTT**-3'

***Arc***

LEFT PRIMER 5'-**CCACCTACCCCTCACCTGTCT**-3'

RIGHT PRIMER 5'-**TGCCTACTTTTCGTTGCCTTTC**-3'

***Bdnf***

LEFT PRIMER 5'-**CGATTAGGTGGCTTCATAGGAGAC**-3'

RIGHT PRIMER 5'-**GAAACAGAACGAACAGAAACAGAGG**-3'

***Btg2***

LEFT PRIMER 5'-**AGGTGGTGGAAGGGAGCAA**-3'

RIGHT PRIMER 5'-**CAGACGGAAGGGACAGGAGA**-3'

***Cfos***

LEFT PRIMER 5'-**CTGCAAGATCCCCAATGACC**-3'

RIGHT PRIMER 5'-**CAGACCCCCAGTCAAGTCCA**-3'

***CfosB***

LEFT PRIMER 5'-**CTCCAAGCGGAGACAGATCAA**-3'

RIGHT PRIMER 5'-**CAGACCCCCAGTCAAGTCCA**-3'

***Cited2***

LEFT PRIMER 5'-**CGACGAGGAAGTGCTTATGTCC**-3'

RIGHT PRIMER 5'-**GGCAGGGAGGGTGATTTCTT**-3'

***Crem***

LEFT PRIMER 5'-**GAATGCAGCTCTCCTCCCATT**-3'

RIGHT PRIMER 5'-**TCGGCTCTCCAGACACTTCAC**-3'

***Cyr61***

LEFT PRIMER 5'-**GGGACTAAAGGTCTCCTGGGTTT**-3'

RIGHT PRIMER 5'-**TCCAACTGCGACTGCGTTACT**-3'

***DUSP1***

LEFT PRIMER 5'-**AGCATCCCTGTGGAGGACAA**-3'

RIGHT PRIMER 5'-**TGAGGTAAGCAAGGCAGATGG**-3'

***DUSP5***

LEFT PRIMER 5'-**CGTCTGGGAGGAAGCACAAG**-3'

RIGHT PRIMER 5'-**CGTCTGGGAGGAAGCACAAG**-3'

***Dusp6***

LEFT PRIMER 5'-**AGTGTCTCATTCCTTCAGTTTCTCTTG**-3'

RIGHT PRIMER 5'-**TCCAGTCCCTGCTTGGGTAG**-3'

***Egr1***

LEFT PRIMER 5'-**GCGAACAACCCTACGAGCAC**-3'

RIGHT PRIMER 5'-**GAAGGCGCTGAGGATGAAGA**-3'

***Egr1B***

LEFT PRIMER 5'-**TCGGCTCCTTTCCTCACTCA**-3'

RIGHT PRIMER 5'-**GTGGTCAGGTGCTCGTAGGG**-3'

***Egr2***

LEFT PRIMER 5'-**CTGCCTGACAGCCTCTACCC**-3'

RIGHT PRIMER 5'-**ATGCCATCTCCAGCCACTCC**-3'

***Egr3***

LEFT PRIMER 5'-**CCTAGCAAGACCCCACTCCA**-3'

RIGHT PRIMER 5'-**CGCCCACAGAACTCACAGG**-3'

***Egr4***

LEFT PRIMER 5'-**TCCACCTGAGCGACTTCTCC**-3'

RIGHT PRIMER 5'-**GCACGCCTCTCCCATCAC**-3'

***Gadd45b***

LEFT PRIMER 5'-**CCTCATTCCCCAGAACAATCC**-3'

RIGHT PRIMER 5'-**CGCACACTCCCCTCTCCTC**-3'

***Homer1***

LEFT PRIMER 5'-**GTGTCCACAGAAGCCAGAGAGGG**-3'

RIGHT PRIMER 5'-**CTTGTAGAGGACCCAGCTTCAGT**-3'

***Irs2***

LEFT PRIMER 5'-**AGCATCCACAGCCAGGAGAC**-3'

RIGHT PRIMER 5'-**GCCCGCAGCACTTTACTCTTT**-3'

***Klf4***

LEFT PRIMER 5'-**CGGACCACCTTGCCTTACAC**-3'

RIGHT PRIMER 5'-**GCTGACTTGCTGGGAACTTGA**-3'

***Mapk10***

LEFT PRIMER 5'-**CCCAGCTAAACCAAGGCTCAC**-3'

RIGHT PRIMER 5'-**CCCAATGCACAATCGAACAA**-3'

***Nfil3***

LEFT PRIMER 5'-**GCCGGACAGCAGAAGGAATA**-3'

RIGHT PRIMER 5'-**AGACCCGATGGAGGAGGAGA**-3'

***Nptx2***

LEFT PRIMER 5'-**TCTGGGGAGTTCAAGGCATCT**-3'

RIGHT PRIMER 5'-**AGCATTTACAGGAGGGGTGGA**-3'

***Npy1r***

LEFT PRIMER 5'-**TGTGTCTCCCGTTCACCTTTG**-3'

RIGHT PRIMER 5'-**GTTTGGTCTCCACCCTCTTGG**-3'

***Nr4a1***

LEFT PRIMER 5'-**GGTAGTGTGCGAGAAGGATTGC**-3'

RIGHT PRIMER 5'-**GGCTGGTTGCTGGTGTTCC**-3'

***Nr4a2***

LEFT PRIMER 5'-**GTGAGGGCTGCAAAGGTTTCT**-3'

RIGHT PRIMER 5'-**CTGTGGGCTCTTCGGTTTTG**-3'

***Nr4a3***

LEFT PRIMER 5'-**AAGGAAGAGAGGCTGGTGTGG**-3'

RIGHT PRIMER 5'-**TAGAACGAAGGCAGGGAGGAG**-3'

***Ntf3***

LEFT PRIMER 5'-**GAGGCACCCAGAGAACCAGA**-3'

RIGHT PRIMER 5'-**TATAAGGGAGGGGGCTCCAA**-3'

***Pcdh8***

LEFT PRIMER 5'-**GAAAGACAGCGGGAAAGGAGA**-3'

RIGHT PRIMER 5'-**AAGGTTGACATCTGGGCTGGT**-3'

***Plk2***

LEFT PRIMER 5'-**ACCTCGGCTCTACCTCCTTCA**-3'

RIGHT PRIMER 5'-**CAGACATCAACAGGGTCGTCAG**-3'

***Ptgs2***

LEFT PRIMER 5'-**ATGACGAGCGACTGTTCCAAA**-3'

RIGHT PRIMER 5'-**GCGGATGCCAGTGATAGAGTG**-3'

***Rasl11b***

LEFT PRIMER 5'-**TGGGAGTAAGGGCAGATGGA**-3'

RIGHT PRIMER 5'-**AGAGAAAGCCTCGTAAATGCTCAC**-3'

***Rgs2***

LEFT PRIMER 5'-**CCGAGGACATTCCATTCTGTCT**-3'

RIGHT PRIMER 5'-**CCCTGTTCCCTCCCTCTGTT**-3'

***Rgs2B***

LEFT PRIMER 5'-**ATGCAAAGTGCCATGTTCCTG**-3'

RIGHT PRIMER 5'-**TTTTAAGAGTGTCCGCTTCATTTTCT**-3'

***Srf***

LEFT PRIMER 5'-**GGGCATTTGGGTGGCTTT**-3'

RIGHT PRIMER 5'-**TCACTCGCCCTGGCTCTATC**-3'

**Table C**

Table containing the list of 33 genes whose up-regulation was confirmed by RT-PCR. The first column contains the official gene symbol and the second and last column contain respectively the average (mean) up-regulation and the standard deviation as observed in three biological replicates.

| **GENE NAME** | **AVERAGE UP-REGULATION** | **STD** |
| --- | --- | --- |
| Atf3 | 3,85 | 0,28 |
| Arc | 7,36 | 1,54 |
| Bdnf | 18,77 | 4,58 |
| Btg2 | 2,49 | 0,42 |
| Cfos | 6,94 | 1,38 |
| Cfos_B | 4,45 | 0,73 |
| Cited2 | 2,89 | 0,14 |
| Crem/Icer | 2,75 | 0,56 |
| Cyr61 | 3,47 | 1,14 |
| Dusp1 | 7,49 | 0,87 |
| Dusp5 | 6,84 | 0,59 |
| Dusp6 | 2,01 | 0,49 |
| Egr1 | 8,05 | 2,05 |
| Egr1_B | 6,84 | 1,44 |
| Egr2 | 12,07 | 1,46 |
| Egr3 | 5,20 | 0,88 |
| Egr4 | 4,89 | 0,81 |
| Gadd45b | 8,33 | 1,62 |
| Homer | 10,90 | 2,66 |
| Irs2 | 2,95 | 0,13 |
| Klf4 | 4,17 | 0,22 |
| Mapk10 | 2,05 | 0,56 |
| Nfil3 | 5,37 | 0,73 |
| Nptx2 | 3,54 | 0,35 |
| Npy1r | 2,47 | 0,19 |
| Nr4a1 | 120,87 | 15,94 |
| Nr4a2 | 20,53 | 4,06 |
| Nr4a3 | 6,23 | 1,06 |
| Ntf3 | 2,70 | 0,86 |
| Pcdh8 | 3,48 | 0,35 |
| Plk2 | 2,65 | 0,43 |
| Ptgs2 | 10,48 | 0,72 |
| Rasl11b | 2,59 | 0,377 |
| Rgs2 | 2,95 | 0,54 |
| Rgs2_B | 3,71 | 0,83 |
| Srf | 2,25 | 0,21 |
