# Supplementary material for: Early Phase of Plasticity-Related Gene Regulation and SRF Dependent Transcription in the Hippocampus
Source: PLoS One. 2013 Jul 23;8(7):e68078. doi: 10.1371/journal.pone.0068078 (PMC3720722; doi:10.1371/journal.pone.0068078)
Supplement: File S2 — Supplementary notes. (DOCX) [file pone.0068078.s002.docx]

**SUPPLEMENTARY NOTES for**

**Early phase of plasticity-related gene regulation and SRF dependent transcription in the hippocampus**

*Giovanni Iacono, Claudio Altafini and Vincent Torre*

**Atf3** is transcriptional repressor that protects hippocampal neurons from apoptosis induced by growth factor withdrawal and from NMDA induced death [[1](file:///F:\varie%20submission\plos\biologia.html#XZhang:2011:J-Neurosci:21451036)]. In vivo, Atf3 reduces brain damage following ischemic insult [[1](file:///F:\varie%20submission\plos\biologia.html#XZhang:2011:J-Neurosci:21451036)]. It also protects in vivo by KA, kainic acid, death [[2](file:///F:\varie%20submission\plos\biologia.html#XFrancis:2004:Brain-Res-Mol-Brain-Res:15135228)][[3](file:///F:\varie%20submission\plos\biologia.html#XZhang:2009:PLoS-Genet:19680447)]

REGULATORS:

mef2 -dissociated hippocampal cultures , E18 - [[4](file:///F:\varie%20submission\plos\biologia.html#XFlavell:2008:Neuron:19109909)]

mef2 -hippocampal cultures- [[1](file:///F:\varie%20submission\plos\biologia.html#XZhang:2011:J-Neurosci:21451036)]

creb -hippocampal cultures- [[1](file:///F:\varie%20submission\plos\biologia.html#XZhang:2011:J-Neurosci:21451036)]

creb -HeLa cells, COS-1 cells- [[5](file:///F:\varie%20submission\plos\biologia.html#XLu:2007:Biochem-J:17014422)]

sp1/3 - colon cancer cell lines- [[6](file:///F:\varie%20submission\plos\biologia.html#XWilson:2010:Cancer-Res:20068171)]

creb -in vivo hippocampus KA, the gene results CREB dependent- [[7](file:///F:\varie%20submission\plos\biologia.html#XLemberger:2008:FASEB-J:18424767)]

FUNCTIONS key words:

**transcription factor, pro-survival**

**Arc** is a IEG which expression is strongly associated with synaptic activity and learning tasks, especially in the context of new memory acquisition [[8](file:///F:\varie%20submission\plos\biologia.html#XGuzowski:1999:Nat-Neurosci:10570490)][[9](file:///F:\varie%20submission\plos\biologia.html#XKelly:2002:Neuroscience:11934470)][[10](file:///F:\varie%20submission\plos\biologia.html#XWang:2006:Cell:16873068)]. Arc was shown to reduce AMPA receptor surface expression [[11](file:///F:\varie%20submission\plos\biologia.html#XChowdhury:2006:Neuron:17088211)][[12](file:///F:\varie%20submission\plos\biologia.html#XRialVerde:2006:Neuron:17088212)] in association with mGluR-dependent LTD but to positively regulate F-actin formation [[13](file:///F:\varie%20submission\plos\biologia.html#XMessaoudi:2007:J-Neurosci:17898216)] and eIF4E phosphorylation [[14](file:///F:\varie%20submission\plos\biologia.html#XBramham:2010:Exp-Brain-Res:19690847)] during late-phase LTP consolidation. To support the evidence that Arc supports only the late LTP maintenance, in Arc knockout mice it results that early phase LTP is enhanced in both the dentate gyrus in vivo and in the CA1 region of acute hippocampal slices [[15](file:///F:\varie%20submission\plos\biologia.html#XPlath:2006:Neuron:17088210)]. Arc results to be involved also in homeostatic scaling of AMPArs associated with (a 2-days) bicuculline treatment, again with a prominent role in AMPArs endocytosis [[16](file:///F:\varie%20submission\plos\biologia.html#XShepherd:2006:Neuron:17088213)]. Recently, several evidences linking Arc to a novel function related to hippocampal neurogenesis and positive regulation of newborn neuron survival have been provided [[17](file:///F:\varie%20submission\plos\biologia.html#XKuipers:2009:PLoS-One:19290048)].

REGULATORS:

mef2 -dissociated hippocampal cultures , E18 - [[4](file:///F:\varie%20submission\plos\biologia.html#XFlavell:2008:Neuron:19109909)]

sp1 -E18 rat cortical cells culures- [[18](file:///F:\varie%20submission\plos\biologia.html#XQiu:2008:Neuron:19081374)]

mef2 -E18 hippocampal cultures- [[19](file:///F:\varie%20submission\plos\biologia.html#XFlavell:2006:Science:16484497)]

srf -cortical neuron cultures- [[20](file:///F:\varie%20submission\plos\biologia.html#XPintchovski:2009:J-Neurosci:19193899)]

srf -dissociated striatal and hippocampal neuronal cultures- [[21](file:///F:\varie%20submission\plos\biologia.html#XKumar:2011:J-Biol-Chem:22179607)]

mef2 -striatopallidal medium spiny neurons - [[22](file:///F:\varie%20submission\plos\biologia.html#XTian:2010:Mol-Cell-Neurosci:20197093)]

creb -dissociated cortical neuronal culture- [[23](file:///F:\varie%20submission\plos\biologia.html#XKawashima:2009:Proc-Natl-Acad-Sci-U-S-A:19116276)]

mef2 -dissociated cortical neuronal culture- [[23](file:///F:\varie%20submission\plos\biologia.html#XKawashima:2009:Proc-Natl-Acad-Sci-U-S-A:19116276)]

srf -dissociated cortical neuronal culture- [[23](file:///F:\varie%20submission\plos\biologia.html#XKawashima:2009:Proc-Natl-Acad-Sci-U-S-A:19116276)]

srf -Primary hippocampal neurons- [[24](file:///F:\varie%20submission\plos\biologia.html#XBenito:2011:J-Neurosci:22171029)]

FUNCTIONS key words:

**effector, late phase LTP, early depotentiation, neurogenesis, pro-survival**

**Bdnf** is a neurothropin which effects are mainly mediated by the TrkB receptor. Infusion of BDNF into the adult brain promotes neurogenesis [[25](file:///F:\varie%20submission\plos\biologia.html#XScharfman:2005:Exp-Neurol:15755552)][[26](file:///F:\varie%20submission\plos\biologia.html#XPencea:2001:J-Neurosci:11517260)]. Besides, BDNF stimulates dendritic and axonal branching in dentate gyrus granule cell cultures [[27](file:///F:\varie%20submission\plos\biologia.html#XDanzer:2002:J-Neurosci:12427830)]. Bdnf promotes LTP [[28](file:///F:\varie%20submission\plos\biologia.html#XXu:2000:J-Neurosci:10995833)] both via pre-synaptic and post-synaptic actions [[29](file:///F:\varie%20submission\plos\biologia.html#XGartner:2006:J-Neurosci:16571757)] and it also activates pro-survival pathways [[30](file:///F:\varie%20submission\plos\biologia.html#XVaillant:1999:J-Cell-Biol:10477751)] [[31](file:///F:\varie%20submission\plos\biologia.html#XChen:2005:Brain-Res-Mol-Brain-Res:15857681)][[32](file:///F:\varie%20submission\plos\biologia.html#XNiu:2011:J-Neuropathol-Exp-Neurol:21666495)]. Bdnf can be harmful in conditions of sensitive to hyperexcitability, since it favors epileptogenesis [[33](file:///F:\varie%20submission\plos\biologia.html#XLahteinen:2002:Eur-J-Neurosci:11886452)] and can sensitize to Kainate-induced seizures [[34](file:///F:\varie%20submission\plos\biologia.html#XRamsden:2003:Brain-Res:12706240)].

Regulators:

creb -cortical neurons cultures E18- [[35](file:///F:\varie%20submission\plos\biologia.html#XTao:1998:Neuron:9581763)]

mef2 -E18 hippocampal cultures- [[19](file:///F:\varie%20submission\plos\biologia.html#XFlavell:2006:Science:16484497)]

mecp2 –dissociated and organotypic hippocampal cultures- [213]

creb -cortical neurons- [[36](file:///F:\varie%20submission\plos\biologia.html#XZheng:2011:PLoS-One:22174809)]

sp1 -NG108-15 neuroblastoma-glioma hybrid cells- [[37](file:///F:\varie%20submission\plos\biologia.html#XTakeuchi:2002:J-Neurochem:12358730)]

cebp/b -NG108-15 neuroblastoma-glioma hybrid cells- [[37](file:///F:\varie%20submission\plos\biologia.html#XTakeuchi:2002:J-Neurochem:12358730)]

FUNCTIONS key words:

**effector, neurogenesis, growth, pro-survival, potentiation, LTP, sensitization**

**Btg2** has shown to interact with the promoters of various proneural genes [[38](file:///F:\varie%20submission\plos\biologia.html#XCanzoniere:2004:J-Neurosci:15056715)], suggesting that it acts as a transcriptional cofactor [[39](file:///F:\varie%20submission\plos\biologia.html#XFarioliVecchioli:2007:FASEB-J:17371797)] [[40](file:///F:\varie%20submission\plos\biologia.html#XPasseri:2006:Mol-Cell-Biol:16782888)]. Btg2 is expressed in the hippocampus in proliferating and differentiating progenitor cells: in the adult hippocampus Btg2 has shown to promote neurogenesis in the DG [[41](file:///F:\varie%20submission\plos\biologia.html#XFarioliVecchioli:2009:PLoS-One:20020054)], and this process was related to the formation of contextual memories. It also protects in vivo by KA, kainic acid, death [[3](file:///F:\varie%20submission\plos\biologia.html#XZhang:2009:PLoS-Genet:19680447)].

REGULATORS:

creb -PC12 cells- [[42](file:///F:\varie%20submission\plos\biologia.html#XFass:2003:J-Biol-Chem:12939274)]

creb -in vivo hippocampus KA, the gene results CREB dependent- [[7](file:///F:\varie%20submission\plos\biologia.html#XLemberger:2008:FASEB-J:18424767)]

FUNCTIONS key words:

**neurogenesis, pro-survival, transcription factor, development?**

**cFos** is a protein which regulates transcription by heterodimerizing into the complex AP-1. Numerous evidences link cFos expression to synaptic activity associated with new memories/tasks/environments concurrence. [[43](file:///F:\varie%20submission\plos\biologia.html#XSokolova:2009:Bull-Exp-Biol-Med:20396702)][[44](file:///F:\varie%20submission\plos\biologia.html#XBisler:2002:J-Chem-Neuroanat:11861125)][[45](file:///F:\varie%20submission\plos\biologia.html#XDragunow:1989:J-Neurosci-Methods:2507830)][[46](file:///F:\varie%20submission\plos\biologia.html#XKadar:2011:Genes-Brain-Behav:20969727)][[47](file:///F:\varie%20submission\plos\biologia.html#XVanElzakker:2008:Learn-Mem:19050162)]. The final effect of cFos-regulated gene expression seems to be necessary to potentiate synaptic transmission in CA3-CA1 synapses [[48](file:///F:\varie%20submission\plos\biologia.html#XFleischmann:2003:J-Neurosci:14534245)][[49](file:///F:\varie%20submission\plos\biologia.html#XGass:2004:Brain-Res-Mol-Brain-Res:15519672)]. cFos is particularly relevant for long-term, but not short term, memories [[50](file:///F:\varie%20submission\plos\biologia.html#XKatche:2010:Proc-Natl-Acad-Sci-U-S-A:20018662)]. cFos -/- mice present impaired kindling-induced development and kindling-induce sprouting of hippocampal granule cell, probably because of loss of transcriptional activation of neurotrophic factors and growth-related genes [[51](file:///F:\varie%20submission\plos\biologia.html#XWatanabe:1996:J-Neurosci:8656277)].

REGULATORS:

srf/elk1 -oranotypic hippocampal- [[52](file:///F:\varie%20submission\plos\biologia.html#XLindecke:2006:Eur-J-Neurosci:16903857)]

mef2 -dissociated hippocampal clutures , E18 - [[4](file:///F:\varie%20submission\plos\biologia.html#XFlavell:2008:Neuron:19109909)]

srf/mkl - cortical neuron cultures - [[53](file:///F:\varie%20submission\plos\biologia.html#XKalita:2006:J-Neurosci:17005865)]

creb -E18 rat cortical cells culures- [[18](file:///F:\varie%20submission\plos\biologia.html#XQiu:2008:Neuron:19081374)]

sp1 -E18 rat cortical cells culures- [[18](file:///F:\varie%20submission\plos\biologia.html#XQiu:2008:Neuron:19081374)]

creb -hippocampal neuron cultures- inverse regulation calcineurin- [[54](file:///F:\varie%20submission\plos\biologia.html#XLam:2009:J-Biol-Chem:19270309)]

srf -hippocampal neuron cultures- inverse regulation calcineurin- [[54](file:///F:\varie%20submission\plos\biologia.html#XLam:2009:J-Biol-Chem:19270309)]

creb -PC12 cells- [[42](file:///F:\varie%20submission\plos\biologia.html#XFass:2003:J-Biol-Chem:12939274)]

c/ebps -primary cortical neurons- [[55](file:///F:\varie%20submission\plos\biologia.html#XCalella:2007:Neural-Dev:17254333)]

sp1/3 - colon cancer cell lines- [[6](file:///F:\varie%20submission\plos\biologia.html#XWilson:2010:Cancer-Res:20068171)]

srf -T98G glioblastoma- [[56](file:///F:\varie%20submission\plos\biologia.html#XTullai:2004:J-Biol-Chem:14769801)]

not/creb -in vivo hippocampus KA, the gene results CREB independent- [[7](file:///F:\varie%20submission\plos\biologia.html#XLemberger:2008:FASEB-J:18424767)]

FUNCTIONS key words:

**transcription factor, potentiation, growth**

**Cited2** is a transcription cofactor [[57](file:///F:\varie%20submission\plos\biologia.html#XBhattacharya:1999:Genes-Dev:9887100)] which is essential for development, since its knock-down is lethal for embryos [[58](file:///F:\varie%20submission\plos\biologia.html#XBamforth:2001:Nat-Genet:11694877)]. In mice cortical neurons Cited2 promotes neuronal death following DNA damage [[59](file:///F:\varie%20submission\plos\biologia.html#XGonzalez:2008:J-Neurosci:18495890)]. In conclusion, it appears that cited2 plays an important, yet unaddressed, role in neuronal differentiation.

REGULATORS:

mef2 -dissociated hippocampal clutures , E18 - [[4](file:///F:\varie%20submission\plos\biologia.html#XFlavell:2008:Neuron:19109909)]

sp1/3 -fibroblasts- [[60](file:///F:\varie%20submission\plos\biologia.html#XHan:2001:J-Biol-Chem:11114295)]

FUNCTIONS key words:

**transcription factor, development**

**Icer** is a potent repressor of cAMP mediated transactivation [[61](file:///F:\varie%20submission\plos\biologia.html#XBorlikova:2009:Mol-Neurobiol:19434522)]. Upregulated during new environment exploration, Icer can potentially repress many genes whose products are critically involved in neuronal plasticity [[61](file:///F:\varie%20submission\plos\biologia.html#XBorlikova:2009:Mol-Neurobiol:19434522)]. In addition to the competition for CRE sites, ICER is proposed decrease the stability of CREB itself [[62](file:///F:\varie%20submission\plos\biologia.html#XMouravlev:2007:Brain-Res:17169345)]. Overexpression of ICER evoked neuronal cell loss in organotypic hippocampal culture [[63](file:///F:\varie%20submission\plos\biologia.html#XMioduszewska:2008:J-Neurosci-Res:17722060)] and Icer was related to pro-apoptotic and neurodegenerative effects in many other studies [[64](file:///F:\varie%20submission\plos\biologia.html#XKlejman:2006:Acta-Neurobiol-Exp-Wars:17265688)][[65](file:///F:\varie%20submission\plos\biologia.html#XJaworski:2003:J-Neurosci:12805292)]. Icer acts to suppress excessive network activity in the kindling model and in the pilocarpine-induced status epilepticus [[66](file:///F:\varie%20submission\plos\biologia.html#XKojima:2008:J-Neurosci:18562617)][[67](file:///F:\varie%20submission\plos\biologia.html#XPorter:2008:Neuroscience:18295410)]. With respect to memory consolidation, in accordance with its CREB antagonist role, Icer seems to impair certain form of long-term memory [[66](file:///F:\varie%20submission\plos\biologia.html#XKojima:2008:J-Neurosci:18562617)].

REGULATORS:

creb -thymoma cells WEHI7.2- [[68](file:///F:\varie%20submission\plos\biologia.html#XKrueger:1999:Mol-Endocrinol:10406470)]

creb -PC12 cells- [[42](file:///F:\varie%20submission\plos\biologia.html#XFass:2003:J-Biol-Chem:12939274)]

creb – AtT20 and PC12 cells. - [[69](file:///F:\varie%20submission\plos\biologia.html#XBrooksKayal:2009:Neurotherapeutics:19332325)]

FUNCTIONS key words:

**transcription factor, anti-survival, depotentiation, anti-LTP?**

**Cyr61** is an extracellular heparin binding protein suggested to have a role in neuronal differentiation process [[70](file:///F:\varie%20submission\plos\biologia.html#XChung:1998:Neurosci-Lett:9832196)]. It is upregulated in immortalized hippocampal cells during death evoked by toxic insults, such as etoposide treatment [[71](file:///F:\varie%20submission\plos\biologia.html#XKim:2003:J-Biol-Chem:12576482)]. In glioma cells Cyr61 promotes proliferation and motility [[72](file:///F:\varie%20submission\plos\biologia.html#XSin:2008:J-Cell-Biochem:18004727)].

REGULATORS:

mef2 -dissociated hippocampal cultures , E18 - [[4](file:///F:\varie%20submission\plos\biologia.html#XFlavell:2008:Neuron:19109909)]

creb/atf2 -primary cultures smooth muscle cells- [[73](file:///F:\varie%20submission\plos\biologia.html#XHan:2003:Eur-J-Biochem:12899698)]

srf/tcf -immortalized hippocampal progenitor cells- [[71](file:///F:\varie%20submission\plos\biologia.html#XKim:2003:J-Biol-Chem:12576482)]

srf/mkl - cortical neuron cultures - [[53](file:///F:\varie%20submission\plos\biologia.html#XKalita:2006:J-Neurosci:17005865)]

creb -A375SM melanoma cell line, creb act as a cyr61 repressor- [[74](file:///F:\varie%20submission\plos\biologia.html#XDobroff:2009:J-Biol-Chem:19632997)]

srf -smooth muscle cells- [[75](file:///F:\varie%20submission\plos\biologia.html#XHanna:2009:J-Biol-Chem:19542562)]

creb -HEK 293, NIH3T3 and other cell lines- [[76](file:///F:\varie%20submission\plos\biologia.html#XMeyuhas:2008:Mol-Cancer-Res:18819928)]

not/creb -in vivo hippocampus KA, the gene results CREB independent- [[7](file:///F:\varie%20submission\plos\biologia.html#XLemberger:2008:FASEB-J:18424767)]

FUNCTIONS key words:

**effector, growth**

**Dusp1** is a protein that catalyzes the dephosphorylation and inactivation of MAP kinase: in particular, dusp1 displays phosphatase activity towards both the stress-activated kinases p38 and JNK, and to a lesser extent, Erk1/2 [[77](file:///F:\varie%20submission\plos\biologia.html#XOwens:2007:Oncogene:17496916)]. In [[78](file:///F:\varie%20submission\plos\biologia.html#XDavis:2000:J-Neurosci:10844026)] it was suggested that dusp1 is involved in the negative feedback loop responsible for fast dephosphorylation of MAPK/ERK. Dusp1 is also involved in BDNF-induced axonal branching: by deactivating JNKs, Dusp1 favors a temporary microtubule destabilization which in turn favors the subsequent initiation of axonal branching [[79](file:///F:\varie%20submission\plos\biologia.html#XJeanneteau:2010:Nat-Neurosci:20935641)].

REGULATORS:

Cebp/b -human lung carcinoma- [[80](file:///F:\varie%20submission\plos\biologia.html#XJohanssonHaque:2008:J-Mol-Endocrinol:18682532)]

creb -pituitary cell line GH4C1, only basal transcription because of calcium sensitive block - [[81](file:///F:\varie%20submission\plos\biologia.html#XRyser:2004:Biochem-J:14609431)]

sp1/3 -pituitary cell line GH4C1, only basal transcription because of calcium sensitive block- [[81](file:///F:\varie%20submission\plos\biologia.html#XRyser:2004:Biochem-J:14609431)]

creb -PC12 cells- [[42](file:///F:\varie%20submission\plos\biologia.html#XFass:2003:J-Biol-Chem:12939274)]

creb -visceral epithelial cells/ podocytes - [[82](file:///F:\varie%20submission\plos\biologia.html#XLu:2008:Mol-Cell-Biol:18625721)]

not/creb -in vivo hippocampus KA, the gene results CREB independent- [[7](file:///F:\varie%20submission\plos\biologia.html#XLemberger:2008:FASEB-J:18424767)]

FUNCTIONS key words:

**effector, erk-inactivation, anti-growth**

**Dusp5** is a nuclear dual-specificity phosphatase that, unlike Dusp1,Dusp2 and Dusp4, is specific for ERK1/2 [[83](file:///F:\varie%20submission\plos\biologia.html#XMandl:2005:Mol-Cell-Biol:15713638)] and not for p38 and JNK. Dusp5 is able to inactivate and ERK1/2 and to cause its nuclear translocation, working as a nuclear anchor for inactive ERK [[83](file:///F:\varie%20submission\plos\biologia.html#XMandl:2005:Mol-Cell-Biol:15713638)] [[84](file:///F:\varie%20submission\plos\biologia.html#XVolmat:2001:J-Cell-Sci:11682603)].

REGULATORS

srf -T98G glioblastoma- [[56](file:///F:\varie%20submission\plos\biologia.html#XTullai:2004:J-Biol-Chem:14769801)]

FUNCTIONS key words:

**effector, erk-inactivation**

**Dusp6** is a protein that catalyzes the dephosphorylation and inactivation of ERK1/2 kinases [[85](file:///F:\varie%20submission\plos\biologia.html#XTarrega:2010:Methods-Mol-Biol:20811991)]. In [[86](file:///F:\varie%20submission\plos\biologia.html#XDomercq:2011:J-Biol-Chem:21300799)] Dusp6 was found to be upregulated during oligodendrocyte death induce by AMPA treatment.

REGULATORS:

not/creb -in vivo hippocampus KA, the gene results CREB independent- [[7](file:///F:\varie%20submission\plos\biologia.html#XLemberger:2008:FASEB-J:18424767)]

FUNCTIONS key words:

**effector, erk-inactivation, pro-anti-survival?**

**Egr1**/zif268 is a zinc finger transcription factor belonging to the EGR family of transcription factors. It is rapidly induced by synaptic activity and it is essential for the proper formation of late phase LTP [[87](file:///F:\varie%20submission\plos\biologia.html#XJones:2001:Nat-Neurosci:11224546)] [[88](file:///F:\varie%20submission\plos\biologia.html#XAbraham:1991:Mol-Neurobiol:1688055)] and for the consolidation of long term memories [[89](file:///F:\varie%20submission\plos\biologia.html#XDavis:2003:Behav-Brain-Res:12798262)] [[90](file:///F:\varie%20submission\plos\biologia.html#XRibeiro:2002:J-Neurosci:12486186)].Egr1 seems not to be required for the establishment of kindling plasticity [[91](file:///F:\varie%20submission\plos\biologia.html#XZheng:1998:Neuroscience:9466414)].While Egr1 involvement in DG-TLP is well established, its involvement in CA1 LTP is less clear and could imply mechanisms different then the ERE-regulated transcription[[92](file:///F:\varie%20submission\plos\biologia.html#XCheval:2011:Hippocampus:21425206)].

REGULATORS

creb -hepatocyte progenitors cell- [[93](file:///F:\varie%20submission\plos\biologia.html#XTur:2010:Cell-Mol-Life-Sci:20582451)]

sp1 -hepatocyte progenitors cell- [[93](file:///F:\varie%20submission\plos\biologia.html#XTur:2010:Cell-Mol-Life-Sci:20582451)]

srf/elk1 -emsa on cloned promoter- [[94](file:///F:\varie%20submission\plos\biologia.html#XWatson:1997:Oncogene:9010223)]

srf/elk1 -oranotypic hippocampal- [[52](file:///F:\varie%20submission\plos\biologia.html#XLindecke:2006:Eur-J-Neurosci:16903857)]

sp1 -E18 rat cortical cells culures- [[18](file:///F:\varie%20submission\plos\biologia.html#XQiu:2008:Neuron:19081374)]

c/ebps -primary cortical neurons- [[55](file:///F:\varie%20submission\plos\biologia.html#XCalella:2007:Neural-Dev:17254333)]

sp1/3 - colon cancer cell lines- [[6](file:///F:\varie%20submission\plos\biologia.html#XWilson:2010:Cancer-Res:20068171)]

srf -T98G glioblastoma- [[56](file:///F:\varie%20submission\plos\biologia.html#XTullai:2004:J-Biol-Chem:14769801)]

creb -hippocampal neuron cultures- [[54](file:///F:\varie%20submission\plos\biologia.html#XLam:2009:J-Biol-Chem:19270309)]

srf -hippocampal neuron cultures - [[54](file:///F:\varie%20submission\plos\biologia.html#XLam:2009:J-Biol-Chem:19270309)]

creb - pancreatic beta-cell line INS-1- [[95](file:///F:\varie%20submission\plos\biologia.html#XKang:2007:Am-J-Physiol-Endocrinol-Metab:16926376)]

creb -hepatoma cells- [[96](file:///F:\varie%20submission\plos\biologia.html#XBauer:2005:Arch-Biochem-Biophys:15910736)]

sp1/3, srf, creb -follicular cells- [[97](file:///F:\varie%20submission\plos\biologia.html#XRussell:2003:Mol-Endocrinol:12554779)]

srf -Primary hippocampal neurons - [[24](file:///F:\varie%20submission\plos\biologia.html#XBenito:2011:J-Neurosci:22171029)]

not/creb -in vivo hippocampus KA, the gene results CREB independent- [[7](file:///F:\varie%20submission\plos\biologia.html#XLemberger:2008:FASEB-J:18424767)]

FUNCTIONS key words:

**transcription factor,LTP**

**Egr2**/krox20 is a zinc finger transcription factor belonging to the EGR family of transcription factors. In [[98](file:///F:\varie%20submission\plos\biologia.html#XInokuchi:1996:Biochem-Biophys-Res-Commun:8619872)] Egr2 was found to be upregulated by stimulation of rat hippocampal perforant pathway, which elicited a long term potentiation: this coincidence suggested a possible role of Egr2 for the maintenance of long-term potentiation. However, in [[99](file:///F:\varie%20submission\plos\biologia.html#XPoirier:2007:Front-Behav-Neurosci:18958188)] Egr2 deficient mice had no impairment in spatial learning and memory, instead they had improved performance in motor learning on a rotarod, and in object recognition memory [[99](file:///F:\varie%20submission\plos\biologia.html#XPoirier:2007:Front-Behav-Neurosci:18958188)].

REGULATORS:

srf/mkl -primary hippocampal cultures- [[100](file:///F:\varie%20submission\plos\biologia.html#XOSullivan:2010:Cereb-Cortex:20016002)]

mecp2 -neuroblastoma cultures- [[101](file:///F:\varie%20submission\plos\biologia.html#XSwanberg:2009:Hum-Mol-Genet:19000991)]

creb -hippocampal neuron cultures- inverse regulation calcineurin- [[54](file:///F:\varie%20submission\plos\biologia.html#XLam:2009:J-Biol-Chem:19270309)]

srf -hippocampal neuron cultures- inverse regulation calcineurin- [[54](file:///F:\varie%20submission\plos\biologia.html#XLam:2009:J-Biol-Chem:19270309)]

c/ebps -primary cortical neurons- [[55](file:///F:\varie%20submission\plos\biologia.html#XCalella:2007:Neural-Dev:17254333)]

srf -T98G glioblastoma- [[56](file:///F:\varie%20submission\plos\biologia.html#XTullai:2004:J-Biol-Chem:14769801)]

not/creb -in vivo hippocampus KA, the gene results CREB independent- [[7](file:///F:\varie%20submission\plos\biologia.html#XLemberger:2008:FASEB-J:18424767)]

FUNCTIONS key words:

**transcription factor, LTP?**

**Egr3**/zif268 is a zinc finger transcription factor belonging to the EGR family of transcription factors. It appears to be essential for hippocampal LTP and for hippocampal dependent memory [[92](file:///F:\varie%20submission\plos\biologia.html#XCheval:2011:Hippocampus:21425206)] in a Egr1 independent manner [[102](file:///F:\varie%20submission\plos\biologia.html#XLi:2007:Mol-Cell-Neurosci:17350282)]. In [[103](file:///F:\varie%20submission\plos\biologia.html#XRoberts:2006:J-Biol-Chem:16901909)] Egr3 was suggested to favor epileptogenesis by upregulating type A GABAr alpha4 subunits. Following Bdnf stimulation, egr3 upregulates NR1 subunit to increase the NMDAr population at postsynaptic compartments [[104](file:///F:\varie%20submission\plos\biologia.html#XKim:2012:J-Neurochem:22035109)].

REGULATORS:

creb -hippocampal neuron cultures- inverse regulation calcineurin- [[54](file:///F:\varie%20submission\plos\biologia.html#XLam:2009:J-Biol-Chem:19270309)]

srf -hippocampal neuron cultures- inverse regulation calcineurin- [[54](file:///F:\varie%20submission\plos\biologia.html#XLam:2009:J-Biol-Chem:19270309)]

sp3 - colon cancer cell lines- [[6](file:///F:\varie%20submission\plos\biologia.html#XWilson:2010:Cancer-Res:20068171)]

creb, srf -primary endothelial cells- [[105](file:///F:\varie%20submission\plos\biologia.html#XSuehiro:2010:Blood:19965691)]

not/creb -in vivo hippocampus KA, the gene results CREB independent- [[7](file:///F:\varie%20submission\plos\biologia.html#XLemberger:2008:FASEB-J:18424767)]

FUNCTIONS key words:

**transcription factor, LTP, potentiation**

**Egr4**/zif268 is a zinc finger transcription factor belonging to the EGR family of transcription factors. In [[106](file:///F:\varie%20submission\plos\biologia.html#XLudwig:2011:J-Neurosci:21228173)],[[107](file:///F:\varie%20submission\plos\biologia.html#XUvarov:2006:J-Neurosci:17192429)] and [[108](file:///F:\varie%20submission\plos\biologia.html#XLudwig:2011:Neural-Plast:21837281)] it was shown that Egr4 can upregulate the transcription of KCC2b, thus suggesting a role for Egr4 in mediating the postnatal reversing of chloride concentration.

REGULATORS: creb -PC12 cells- [[42](file:///F:\varie%20submission\plos\biologia.html#XFass:2003:J-Biol-Chem:12939274)]

FUNCTIONS key words:

**transcription factor, development**

**Gadd45b** is a gene which encoded protein was shown to promote adult neurogenesis and dendritic growth of newborn neurons in mouse hippocampus [[109](file:///F:\varie%20submission\plos\biologia.html#XMa:2009:Science:19119186)]. It acts by demethylating DNA at specific promoters, for instance the Bdnf one[[109](file:///F:\varie%20submission\plos\biologia.html#XMa:2009:Science:19119186)]. Gadd45b is also strongly pro-survival and protects neurons by kainic acid death [[3](file:///F:\varie%20submission\plos\biologia.html#XZhang:2009:PLoS-Genet:19680447)].

REGULATORS:

sp1 -colorectal carcinoma cells- [[110](file:///F:\varie%20submission\plos\biologia.html#XZumbrun:2009:J-Cell-Biochem:19834918)]

sp1 -hepatocellular carcinoma - [[111](file:///F:\varie%20submission\plos\biologia.html#XOu:2010:Cancer-Res:21062976)]

creb -in vivo hippocampus KA, the gene results CREB dependent- [[7](file:///F:\varie%20submission\plos\biologia.html#XLemberger:2008:FASEB-J:18424767)]

FUNCTIONS key words:

**effector, neurogenesis, growth, pro-survival**

**Homer1a** is activity-inducible short-splice variant of the homer protein that lacks the dimerization domain: due to this peculiar feature homer1a inhibits dendritic spine morphogenesis and synaptic transmission, by decreasing the size of PSD95 clusters and the number of surface AMPArs and NMDArs [[112](file:///F:\varie%20submission\plos\biologia.html#XSala:2003:J-Neurosci:12867517)]. Homer1a besides prevents the clustering of the inositol trisphosphate receptor (IP3R) and other Ca++ sensor in proximity to the Ca++ channels inside the PSD complex [[113](file:///F:\varie%20submission\plos\biologia.html#XSala:2005:J-Neurosci:15872106)]. Since the initial activity-induced disassembly of postsynaptic proteins may be prerequisite for the subsequent potentiation to occur[[114](file:///F:\varie%20submission\plos\biologia.html#XLynch:2007:Neuropharmacology:16949110)] it was suggested the Homer1a is one of the actors involved in the disassembly [[115](file:///F:\varie%20submission\plos\biologia.html#XInoue:2007:Neuroscience:18006237)]. Choerently with the previous results, a study reported that Homer1a overexpression prevents the proper formation and maintenance of LTP in the hippocampus [[116](file:///F:\varie%20submission\plos\biologia.html#XCelikel:2007:Front-Neurosci:18982121)].

REGULATORS:

mef2 -dissociated hippocampal clutures , E18 - [[4](file:///F:\varie%20submission\plos\biologia.html#XFlavell:2008:Neuron:19109909)]

FUNCTIONS key words:

**effector, regulation syn.transmission, growth**

**Irs2**, the insulin receptor substrate 2, is a mediator of the insulin effects in the central nervous system. Irs2 was reported to support LTP evoked by HFS in mice brain slices [[117](file:///F:\varie%20submission\plos\biologia.html#XMartin:2011:Cereb-Cortex:21955917)]. During development, irs2 deficiency impairs brain growth, reducing neuronal proliferation [[118](file:///F:\varie%20submission\plos\biologia.html#XSchubert:2003:J-Neurosci:12904469)]. However, in another study irs2 appears to be a negative regulator of memory formation: irs2 brain selective knockout generates mice with enhanced hippocampal spatial reference memory and with increased number of excitatory synapses per neuron in CA1 area [[119](file:///F:\varie%20submission\plos\biologia.html#XIrvine:2011:Learn-Mem:21597043)].

REGULATORS:

creb -mouse beta cells- [[120](file:///F:\varie%20submission\plos\biologia.html#XPersaud:2011:Diabetologia:21301804)],

creb -pancreatic B cell line MIN6 insulinoma cells- [[121](file:///F:\varie%20submission\plos\biologia.html#XJhala:2003:Genes-Dev:12842910)]

sp1 -neuroblastoma - [[122](file:///F:\varie%20submission\plos\biologia.html#XUdelhoven:2010:J-Endocrinol:19875459)] ,

sp1 - hepatocellular carcinoma - [[123](file:///F:\varie%20submission\plos\biologia.html#XUdelhoven:2010:J-Mol-Endocrinol:19755487)]

torc2 -hypothalamic cultures- [[124](file:///F:\varie%20submission\plos\biologia.html#XLerner:2009:EMBO-Rep:19713961)]

FUNCTIONS key words:

**effector, LTP?, growth, proliferation, development**

**Klf4** belongs to the Kruppel-like family of transcription factors, which are generally known to have roles in cell proliferation, differentiation and survival [[125](file:///F:\varie%20submission\plos\biologia.html#XGarrettSinha:1996:J-Biol-Chem:8940147)][[126](file:///F:\varie%20submission\plos\biologia.html#XRowland:2006:Nat-Rev-Cancer:16372018)][[127](file:///F:\varie%20submission\plos\biologia.html#XRowland:2005:Nat-Cell-Biol:16244670)]. In particular, Klf4 was shown to act independently of cell survival to suppress axon and dendrite initiation and elongation in hippocampal neuron in vitro [[128](file:///F:\varie%20submission\plos\biologia.html#XMoore:2009:Science:19815778)].In [[129](file:///F:\varie%20submission\plos\biologia.html#XZhu:2009:Brain-Res:19041854)] Klf4 was suggested to induce quiescent neurons to re-enter in the cell cycle, thus promoting proliferation, and to sensitize neurons to NMDA-induced caspase-3 activity.

REGULATORS:

not/creb -in vivo hippocampus KA, the gene results CREB independent- [[7](file:///F:\varie%20submission\plos\biologia.html#XLemberger:2008:FASEB-J:18424767)]

sp1 -myoblast cell line C2C12- [[130](file:///F:\varie%20submission\plos\biologia.html#XSunadome:2011:Dev-Cell:21316587)]

sp1 -smooth muscle cells- [[131](file:///F:\varie%20submission\plos\biologia.html#XDeaton:2009:Am-J-Physiol-Heart-Circ-Physiol:19168719)]

mef2 -Endothelial cell culture- [[132](file:///F:\varie%20submission\plos\biologia.html#XVillarreal:2010:Biochem-Biophys-Res-Commun:19968965)]

FUNCTIONS key words:

**transcription factor,suppress growth,sensitization**

**Mapk10**/JNK3 is a kinase belonging to the MAPK family [[133](file:///F:\varie%20submission\plos\biologia.html#XWeston:2007:Curr-Opin-Cell-Biol:17303404)] and is expressed mainly in the brain [[134](file:///F:\varie%20submission\plos\biologia.html#XBode:2007:Mol-Carcinog:17538955)]. JNKs have been related to seemingly contradictory cellular responses: they were reported to act both pro-apoptotically [[135](file:///F:\varie%20submission\plos\biologia.html#XHan:2008:FEBS-Lett:18307989)][[136](file:///F:\varie%20submission\plos\biologia.html#XZhao:2007:Cell-Signal:17161586)] and pro-survival [[137](file:///F:\varie%20submission\plos\biologia.html#XYu:2004:Mol-Cell:14967141)]. The final effect depends on the cellular context and on the duration of JNKs activation[[138](file:///F:\varie%20submission\plos\biologia.html#XVentura:2006:Mol-Cell:16507367)].Several evidences links JNK to plasticity of hippocampal formation: whereas acute over-activation of JNK by pathophysiological concentrations of cytokines is detrimental to LTP, physiologic activation of JNK appears necessary for the induction of LTD [[139](file:///F:\varie%20submission\plos\biologia.html#XCurran:2003:Neuroscience:12699771)]. In another study JNK, together with ERK, was demonstrated to promote LTP associated with pain-stumuli[[140](file:///F:\varie%20submission\plos\biologia.html#XLiu:2011:Brain-Res:21284942)]. JNK also mediates synaptic AMPA receptors removal during depotentiation [[141](file:///F:\varie%20submission\plos\biologia.html#XZhu:2005:Neuron:15953419)]. JNK3 has been recently implied in the process of neuritogenesis and reorganization of the cytoskeleton [[142](file:///F:\varie%20submission\plos\biologia.html#XBarnat:2010:J-Neurosci:20534829)][[143](file:///F:\varie%20submission\plos\biologia.html#XOliva:2006:J-Neurosci:16971530)].

FUNCTIONS key words:

**effector, pro-survival, LTP, LTD, depotentiation**

**Nfil3**/E4BP4 is a transcription factor that is involved in cell survival. In cultures of primary rat motoneurons Nfil3 was reported to protects against cell death triggered by removal of neurotrophic factors or activation of death receptors and to strongly enhance neuronal cell size and axonal growth [[144](file:///F:\varie%20submission\plos\biologia.html#XJunghans:2004:Development:15306565)]. Neuroprotective effects were then confirmed also in vivo [[144](file:///F:\varie%20submission\plos\biologia.html#XJunghans:2004:Development:15306565)].However, in primary adult DRG neurons, Nfil3 was found to reduce neurite outgrowth by repressing the expression of CREB-induced genes: since CREB itself upregulates Nfil3, it was therefore suggested the existence of a CREB-Nfil3 incoherent feedforward loop, needed to increase responsiveness of neurite outgrowth genes. [[145](file:///F:\varie%20submission\plos\biologia.html#XMacGillavry:2009:J-Neurosci:20007478)] [[146](file:///F:\varie%20submission\plos\biologia.html#XMacGillavry:2011:Mol-Cell-Neurosci:21112399)].

REGULATORS:

mef2 -dissociated hippocampal cultures , E18 - [[4](file:///F:\varie%20submission\plos\biologia.html#XFlavell:2008:Neuron:19109909)]

creb - DRG neurons in vivo - [[145](file:///F:\varie%20submission\plos\biologia.html#XMacGillavry:2009:J-Neurosci:20007478)]

creb -in vivo hippocampus KA, the gene results CREB dependent- [[7](file:///F:\varie%20submission\plos\biologia.html#XLemberger:2008:FASEB-J:18424767)]

FUNCTIONS key words:

**transcription factor, pro-survival, growth?**

**Nptx2**/narp is a member of the neuronal pentraxins. Narp was reported to induce AMPAr clustering at post-synaptic excitatory terminals [[147](file:///F:\varie%20submission\plos\biologia.html#XOBrien:1999:Neuron:10399937)][[148](file:///F:\varie%20submission\plos\biologia.html#XOBrien:2002:J-Neurosci:12040056)]. More precisely, Narp during episodes of increased neuronal activity [[149](file:///F:\varie%20submission\plos\biologia.html#XReti:2000:Neuropsychopharmacology:10989271)] recruits AMPAr at excitatory projections onto gabaergic interneurons in order to increase their inhibitory force [[150](file:///F:\varie%20submission\plos\biologia.html#XChang:2010:Nat-Neurosci:20729843)]; therefore it is characterized by a homeostatic effect. The suppression of network excitability supported by Narp protects the brain from kindling-induced seizures [[150](file:///F:\varie%20submission\plos\biologia.html#XChang:2010:Nat-Neurosci:20729843)].

REGULATORS:

Srf -Primary hippocampal neurons- [[24](file:///F:\varie%20submission\plos\biologia.html#XBenito:2011:J-Neurosci:22171029)]

FUNCTIONS key words:

**effector, depotentiation**

**Npy1r** is a G protein-coupled receptor for NPY, neuropeptide Y. NPY, acting on NPY1r, exerts trophic and attraction effects on prenatal dorsal root ganglion cells [[151](file:///F:\varie%20submission\plos\biologia.html#XHokfelt:2008:Nutrition:18725084)].Npy1r is also associated with modest sensitization to kainate-induce seizures [[152](file:///F:\varie%20submission\plos\biologia.html#XOlesen:2011:J-Neurosci-Res:21971867)]: besides, Npy1r was suggested to mediate an excitatory component of NPY neurotransmission in epilepsy [[153](file:///F:\varie%20submission\plos\biologia.html#XBrooks:1987:Brain-Res:3594219)] and to exert proconvulsant effects in seizure models[[154](file:///F:\varie%20submission\plos\biologia.html#XReibel:2001:Peptides:11287111)].NPY acting on Npy1r in the hippocampus DG promotes cellular proliferation of amplifying neural progenitors and neuroblasts, therefore promoting hippocampal neurogenesis [[155](file:///F:\varie%20submission\plos\biologia.html#XDecressac:2011:Hippocampus:20095007)].

FUNCTIONS key words:

**effector, pro-survival, potentiation**

**Nr4a1**/Nur77 is a member of the to the thyroid/steroid receptor superfamily. Evoked by synaptic activity in mice hippocampal cultures, it promotes neuronal survival by rendering mitochondria more resistant to stress and toxic insults [[3](file:///F:\varie%20submission\plos\biologia.html#XZhang:2009:PLoS-Genet:19680447)]. It also protects in vivo by KA, kainic acid, death [[3](file:///F:\varie%20submission\plos\biologia.html#XZhang:2009:PLoS-Genet:19680447)]. Nur77, upregulated by MEF2, inhibits the dendritic claw differentiation in cerebellar granule neurons [[156](file:///F:\varie%20submission\plos\biologia.html#XShalizi:2006:Science:16484498)]. Nr4a1 has recently proved to exert neuroprotective effects in mouse embryonic stem cell-derived neurons against various neurotoxic stimuli, including glutamate [[157](file:///F:\varie%20submission\plos\biologia.html#XVolakakis:2010:Proc-Natl-Acad-Sci-U-S-A:20566846)]

REGULATORS:

mef2 -pc12 cells and hippocampal neurons cultures / repressor role- [[158](file:///F:\varie%20submission\plos\biologia.html#XLam:2010:J-Neurochem:19968756)]

creb -pc12 cells and hippocampal neurons cultures- [[158](file:///F:\varie%20submission\plos\biologia.html#XLam:2010:J-Neurochem:19968756)]

mef2 -dissociated hippocampal clutures , E18 - [[4](file:///F:\varie%20submission\plos\biologia.html#XFlavell:2008:Neuron:19109909)]

creb -hippocampal neuron cultures - [[54](file:///F:\varie%20submission\plos\biologia.html#XLam:2009:J-Biol-Chem:19270309)]

mef2 -hippocampal neuron cultures - [[54](file:///F:\varie%20submission\plos\biologia.html#XLam:2009:J-Biol-Chem:19270309)]

mef2 -cerebellar cortex- [[156](file:///F:\varie%20submission\plos\biologia.html#XShalizi:2006:Science:16484498)]

creb -PC12 cells- [[42](file:///F:\varie%20submission\plos\biologia.html#XFass:2003:J-Biol-Chem:12939274)]

mef2 -striatopallidal medium spiny neurons - [[22](file:///F:\varie%20submission\plos\biologia.html#XTian:2010:Mol-Cell-Neurosci:20197093)]

srf -T98G glioblastoma- [[56](file:///F:\varie%20submission\plos\biologia.html#XTullai:2004:J-Biol-Chem:14769801)]

creb -in vivo hippocampus KA, the gene results CREB dependent- [[7](file:///F:\varie%20submission\plos\biologia.html#XLemberger:2008:FASEB-J:18424767)]

srf/tax -T-cell various lines- [[159](file:///F:\varie%20submission\plos\biologia.html#XWinter:2007:J-Virol:17376895)]

srf -NIH 3T3 fibroblasts - [[160](file:///F:\varie%20submission\plos\biologia.html#XWilliams:1993:Mol-Cell-Biol:8413214)]

srf/elk-1 -NIH 3T3 fibroblasts- [[161](file:///F:\varie%20submission\plos\biologia.html#XLatinkic:1996:Nucleic-Acids-Res:8614640)]

sp1 -macrophages - [[162](file:///F:\varie%20submission\plos\biologia.html#XKim:2003:J-Exp-Med:12782711)]

creb -neural c17.2 cells- [[157](file:///F:\varie%20submission\plos\biologia.html#XVolakakis:2010:Proc-Natl-Acad-Sci-U-S-A:20566846)]

FUNCTIONS key words:

**transcription factor, pro-survival, suppress growth**

**Nr4a2**/Nurr1/NGFI-B, unlike the related Nor-1 protein, seems not to be required for the establishment of kindling plasticity [[91](file:///F:\varie%20submission\plos\biologia.html#XZheng:1998:Neuroscience:9466414)]. Nurr1 is expressed in the hippocampus during spatial learning [[163](file:///F:\varie%20submission\plos\biologia.html#XPenadeOrtiz:2000:Neurobiol-Learn-Mem:10933901)]. When Nurr1 function is disrupted in the CA3 area spatial memory processes are compromised, possibly because of the lack of proper regulation of Nurr1 target genes, such as contactin, which is related to the establishment of synaptic connections [[164](file:///F:\varie%20submission\plos\biologia.html#XColonCesario:2006:Learn-Mem:17142303)]. Nurr1 is upregulated after KA treatment, thus there subsist a possible role in neuronal survival/vulnerability for Nurr1 [[165](file:///F:\varie%20submission\plos\biologia.html#XCrispino:1998:Brain-Res-Mol-Brain-Res:9729370)]. Indeed Nurr1 has been linked to strong antiapoptotic effects in neuro2a HEK293 and A549 cell lines [[166](file:///F:\varie%20submission\plos\biologia.html#XZhang:2009:Mol-Cancer-Res:19671681)]. Nurr1 has recently proved to exert neuroprotective effects in mouse embryonic stem cell-derived neurons, protecting them against various neurotoxic stimuli, including glutamate. [[157](file:///F:\varie%20submission\plos\biologia.html#XVolakakis:2010:Proc-Natl-Acad-Sci-U-S-A:20566846)].

REGULATORS:

mef2 -dissociated hippocampal clutures , E18 - [[4](file:///F:\varie%20submission\plos\biologia.html#XFlavell:2008:Neuron:19109909)]

creb -in vivo hippocampus KA, the gene results CREB dependent- [[7](file:///F:\varie%20submission\plos\biologia.html#XLemberger:2008:FASEB-J:18424767)]

srf – in silico - [[167](file:///F:\varie%20submission\plos\biologia.html#XCastillo:1997:Genomics:9143501)]

creb -neuroblastoma- [[168](file:///F:\varie%20submission\plos\biologia.html#XJi:2011:Br-J-Pharmacol:22188298)]

creb -embryonic fibroblasts- [[169](file:///F:\varie%20submission\plos\biologia.html#XDarragh:2005:Biochem-J:15910281)]

creb -neuroblastoma N2A and C6 glioma cell lines- [[170](file:///F:\varie%20submission\plos\biologia.html#XLee:2004:Neuroreport:15106839)]

creb -neural c17.2 cells- [[157](file:///F:\varie%20submission\plos\biologia.html#XVolakakis:2010:Proc-Natl-Acad-Sci-U-S-A:20566846)]

CRTC1,torc -in vivo hippocampus - [[171](file:///F:\varie%20submission\plos\biologia.html#XEspana:2010:J-Neurosci:20631169)]

FUNCTIONS key words:

**transcription factor, pro-survival, reg.syn.transmission**

**Nr4a3**/NOR1 Induced in CA1 and CA3 areas of the rat hippocampus following depolarization [[172](file:///F:\varie%20submission\plos\biologia.html#XKim:2006:Mol-Cells:16951544)] or following exposure to a novel environment [[173](file:///F:\varie%20submission\plos\biologia.html#XSun:2007:J-Neurochem:17116234)] , NOR-1 is a zinc-finger transcription factor belonging to the thyroid/steroid receptor superfamily. NOR-1-/- mice present defective postnatal hippocampal development, with reduced axonal projections from dentate gyrus granular and mossy cells and early postnatal death of CA1 pyramidal neurons, which in turn results in increased susceptibility to Kainate-induced seizures [[174](file:///F:\varie%20submission\plos\biologia.html#XPonnio:2004:Mol-Cell-Biol:15456880)]. NOR-1 has recently proved to exert neuroprotective effects in mouse embryonic stem cell-derived neurons against various neurotoxic stimuli, including glutamate [[157](file:///F:\varie%20submission\plos\biologia.html#XVolakakis:2010:Proc-Natl-Acad-Sci-U-S-A:20566846)].

REGULATORS:

mef2 -dissociated hippocampal clutures , E18 - [[4](file:///F:\varie%20submission\plos\biologia.html#XFlavell:2008:Neuron:19109909)]

creb -hippocampal neuron cultures- [[54](file:///F:\varie%20submission\plos\biologia.html#XLam:2009:J-Biol-Chem:19270309)]

mef2 -hippocampal neuron cultures- [[54](file:///F:\varie%20submission\plos\biologia.html#XLam:2009:J-Biol-Chem:19270309)]

creb -PC12 cells- [[42](file:///F:\varie%20submission\plos\biologia.html#XFass:2003:J-Biol-Chem:12939274)]

creb -embryonic fibroblasts- [169]

creb -neural c17.2 cells- [[157](file:///F:\varie%20submission\plos\biologia.html#XVolakakis:2010:Proc-Natl-Acad-Sci-U-S-A:20566846)]

FUNCTIONS key words:

**transcription factor, development, pro-survival**

**Ntf3** a neurothropin which effects are mainly mediated by the TrkC and TrkB receptor. Nt-3 doesn’t seem to have a role in LTP establishment [[175](file:///F:\varie%20submission\plos\biologia.html#XChen:1999:J-Neurosci:10479698)]. However, LTP inductions triggers a Nt-3 upregulation in the Dentate Gyrus[[176](file:///F:\varie%20submission\plos\biologia.html#XBramham:1996:J-Comp-Neurol:8725345)]. NT-3 was shown to promote corticospinal axon growth in organotypic cultures[177]. In hippocampal precursor cells simultaneous action of BDNF and NT3 promoted differentiation and neurite elongation [[178](file:///F:\varie%20submission\plos\biologia.html#XYoo:2007:J-Microbiol-Biotechnol:18167452)]. There are some hints that Nt-3 could enhance, like Bdnf, glutamatergic synaptic transmission hippocampus [[179](file:///F:\varie%20submission\plos\biologia.html#XHolm:1997:Proc-Natl-Acad-Sci-U-S-A:9023372)].Besides, Nt-3 protect CNS neurons against metabolic/excitotoxic insults [[180](file:///F:\varie%20submission\plos\biologia.html#XCheng:1994:Brain-Res:7911729)] [[181](file:///F:\varie%20submission\plos\biologia.html#XXu:2002:Neuroscience:12453498)]. The Nt-3 receptor, TrkC, is required for the maturation of granule cell axons;indeed, its knock-out reduces both the number and the size of mossy axonal terminals [[182](file:///F:\varie%20submission\plos\biologia.html#XOtal:2005:Cell-Tissue-Res:15726425)]. Coherently with the latter study, continuous intraventricular infusion of Nt-3 triggers mossy fiber sprouting [[181](file:///F:\varie%20submission\plos\biologia.html#XXu:2002:Neuroscience:12453498)].

REGULATORS :

Sp3/4 -primary cortical neurons cultures- [[183](file:///F:\varie%20submission\plos\biologia.html#XIshimaru:2007:J-Neurochem:17059557)]

FUNCTIONS key words:

**effector, growth, potentiation?, pro-survival**

**Pcdh8**/arcadlin is a member of the protocadherin family [[184](file:///F:\varie%20submission\plos\biologia.html#XYamagata:1999:J-Biol-Chem:10383464)] that in hippocampal neurons localizes specifically in excitatory post-synaptic terminals [[185](file:///F:\varie%20submission\plos\biologia.html#XYasuda:2007:Neuron:17988630)]: activated upon synaptic activity, pcdh8 triggers N-cadherin endocytosis, reducing the number of excitatory dendritic spines [[185](file:///F:\varie%20submission\plos\biologia.html#XYasuda:2007:Neuron:17988630)].

FUNCTIONS key words:

**effector, depotentiation**

**Plk2**/snk is an activity-inducible member of the polo-like family of serine/threonine kinases. Plk2 effects, mediated by Ras and Rap signaling, promote a reduction in spine number and spine size, together with a loss of AMPAr both in proximal and distal dendrites [[186](file:///F:\varie%20submission\plos\biologia.html#XLee:2011:Neuron:21382555)]. During epileptiform activity in rat hippocampal slices Plk2 is required for the activity-dependent reduction in membrane excitability of pyramidal neurons [[187](file:///F:\varie%20submission\plos\biologia.html#XSeeburg:2008:J-Neurosci:18579731)].

REGULATORS:

not/creb -in vivo hippocampus KA, the gene results CREB independent- [[7](file:///F:\varie%20submission\plos\biologia.html#XLemberger:2008:FASEB-J:18424767)].

FUNCTIONS key words:

**effector, depotentiation**

**Cox2**/Ptgs2 is an enzyme that catalyzes the conversion of arachidonic acid products to prostaglandin [[188](file:///F:\varie%20submission\plos\biologia.html#XYang:2008:Curr-Pharm-Des:18537667)]: increased Cox2 expression significantly enhances basal synaptic transmission and augments LTP in mouse hippocampus [[189](file:///F:\varie%20submission\plos\biologia.html#XYang:2008:Mol-Cell-Neurosci:18295507)]. The enhancement of hippocampal glutamatergic synaptic transmission promoted by Ptgs2 can be neurotoxic [[190](file:///F:\varie%20submission\plos\biologia.html#XSang:2007:J-Neurochem:17539917)]. The inhibition of Ptgs2 has shown to reduce LTP [[191](file:///F:\varie%20submission\plos\biologia.html#XSlanina:2005:Neuropharmacology:15950248)][[192](file:///F:\varie%20submission\plos\biologia.html#XChen:2002:J-Neurophysiol:12037188)]. In primary cortical neurons ptgs2 presents a protective role for DNA damage induced by oxidative, genotoxic, excitotoxic stresses and by ischemic injury [[193](file:///F:\varie%20submission\plos\biologia.html#XLee:2006:FASEB-J:17012241)].

REGULATORS:

C/EBPb -medullary interstitial cells cultures- [[194](file:///F:\varie%20submission\plos\biologia.html#XChen:2005:J-Biol-Chem:15713664)]

not/creb -in vivo hippocampus KA, the gene results CREB independent- [[7](file:///F:\varie%20submission\plos\biologia.html#XLemberger:2008:FASEB-J:18424767)]

C/ebp -epidermoid carcinoma A431 cells- [[195](file:///F:\varie%20submission\plos\biologia.html#XWang:2008:Nucleic-Acids-Res:18820298)]

sp1/3 -primary cortical neurons- [[193](file:///F:\varie%20submission\plos\biologia.html#XLee:2006:FASEB-J:17012241)]

sp1/3 -glioma cell lines- [[196](file:///F:\varie%20submission\plos\biologia.html#XXu:2007:Cancer-Res:17616668)]

c/ebpb -TR neuroblastoma cells- [[197](file:///F:\varie%20submission\plos\biologia.html#XCortesCanteli:2004:J-Biol-Chem:14736879)]

creb -in vivo mutant mice , brain- [[198](file:///F:\varie%20submission\plos\biologia.html#XLee:2007:Neurobiol-Dis:17029965)]

FUNCTIONS key words:

**effector, potentiation, LTP, survival**

**Rasl11b** is a Ras-like GTPase poorly characterized [[199](file:///F:\varie%20submission\plos\biologia.html#XStolle:2007:Biochim-Biophys-Acta:17628721)].

FUNCTIONS key words:

**effector**

**Rgs2** is one of the small members of the regulators of G protein signaling superfamily. In vitro no particular effect of Rgs2 knockout was observed with respect to Schaffer-collaterals/CA1 LTP [[200](file:///F:\varie%20submission\plos\biologia.html#XOliveiraDosSantos:2000:Proc-Natl-Acad-Sci-U-S-A:11027316)], Nonetheless, Rgs2 knockout mice studied in vivo showed enhanced LTP at CA1 pyramidal cell, possibly generated by a prolonged activation of mGluRs in mutant mice, with subsequent increased calcium release from internal stores [[201](file:///F:\varie%20submission\plos\biologia.html#XHutchison:2009:Hippocampus:19294649)]. Rgs is also involved in the regulation of presynaptic plasticity [[201](file:///F:\varie%20submission\plos\biologia.html#XHutchison:2009:Hippocampus:19294649)][[202](file:///F:\varie%20submission\plos\biologia.html#XHan:2006:Neuron:16950156)].A link between Rgs2 activity and level of phospo-ERK was found too [[203](file:///F:\varie%20submission\plos\biologia.html#XSeredenina:2011:PLoS-One:21779398)].

REGULATORS:

Sp1 -catecholaminergic- CATH.a cell line- [[204](file:///F:\varie%20submission\plos\biologia.html#XSalim:2011:FEBS-Lett:21510939)]

Sp1 - preadipocytes - [[205](file:///F:\varie%20submission\plos\biologia.html#XCheng:2008:J-Cell-Biochem:18726905)]

not/creb -in vivo hippocampus KA, the gene results CREB independent- [[7](file:///F:\varie%20submission\plos\biologia.html#XLemberger:2008:FASEB-J:18424767)]

creb - vascular smooth muscle cell primary cultures- [[206](file:///F:\varie%20submission\plos\biologia.html#XXie:2011:J-Biol-Chem:22057271)]

FUNCTIONS key words:

**effector, anti-LTP, depotentiation**

**Srf** belongs the MADS-box superfamily of transcription factors. It binds and recognizes the SRE element in the promoter regions of regulated genes: phosphorylated by Rsk2/Msk following episodes of synaptic activity [[207](file:///F:\varie%20submission\plos\biologia.html#XGreer:2008:Neuron:18817726)][[208](file:///F:\varie%20submission\plos\biologia.html#XHeidenreich:1999:J-Biol-Chem:10318869)], the SRF regulates many of the genes involved in synaptic-plasticity events[[20](file:///F:\varie%20submission\plos\biologia.html#XPintchovski:2009:J-Neurosci:19193899)][[71](file:///F:\varie%20submission\plos\biologia.html#XKim:2003:J-Biol-Chem:12576482)][[78](file:///F:\varie%20submission\plos\biologia.html#XDavis:2000:J-Neurosci:10844026)][[209](file:///F:\varie%20submission\plos\biologia.html#XJohnson:2011:Behav-Brain-Res:21329726)]. Animals in which SRF is deleted from the forebrain present a severely hypoplastic hippocampus and die at 3 weeks postnatal [[210](file:///F:\varie%20submission\plos\biologia.html#XAlberti:2005:Proc-Natl-Acad-Sci-U-S-A:15837932)]. Srf in the adult brain was suggested not to be related to survival transcriptional programs but rather to be related to plasticity and use-dependent modifications of synapses, with particular regard to LTP establishment [[211](file:///F:\varie%20submission\plos\biologia.html#XRamanan:2005:Nat-Neurosci:15880109)]. However, LTD establishment was severely impaired too in Srf forebrain-restricted deficient animals [[212](file:///F:\varie%20submission\plos\biologia.html#XEtkin:2006:Neuron:16600861)].

REGULATORS:

srf/mkl - cortical neuron cultures - [[53](file:///F:\varie%20submission\plos\biologia.html#XKalita:2006:J-Neurosci:17005865)]

srf -T98G glioblastoma- [[56](file:///F:\varie%20submission\plos\biologia.html#XTullai:2004:J-Biol-Chem:14769801)]

FUNCTIONS key words:

**transcription factor, development, LTP, LTD**

**References**

[1]   Zhang SJ, Buchthal B, Lau D, Hayer S, Dick O, et al. (2011) A signaling cascade of nuclear calcium-creb-atf3 activated by synaptic nmda receptors defines a gene repression module that protects against extrasynaptic nmda receptor-induced neuronal cell death and ischemic brain damage. J Neurosci 31: 4978-4990.

[2]   Francis JS, Dragunow M, During MJ (2004) Over expression of atf-3 protects rat hippocampal neurons from in vivo injection of kainic acid. Brain Res Mol Brain Res 124: 199-203.

[3]   Zhang SJ, Zou M, Lu L, Lau D, Ditzel DA, et al. (2009) Nuclear calcium signaling controls expression of a large gene pool: identification of a gene program for acquired neuroprotection induced by synaptic activity. PLoS Genet 5.

[4]   Flavell SW, Kim TK, Gray JM, Harmin DA, Hemberg M, et al. (2008) Genome-wide analysis of mef2 transcriptional program reveals synaptic target genes and neuronal activity-dependent polyadenylation site selection. Neuron 60: 1022-1038.

[5]   Lu D, Chen J, Hai T (2007) The regulation of atf3 gene expression by mitogen-activated protein kinases. Biochem J 401: 559-567.

[6]   Wilson AJ, Chueh AC, Tögel L, Corner GA, Ahmed N, et al. (2010) Apoptotic sensitivity of colon cancer cells to histone deacetylase inhibitors is mediated by an sp1/sp3-activated transcriptional program involving immediate-early gene induction. Cancer Res 70: 609-620.

[7]   Lemberger T, Parkitna JR, Chai M, Schütz G, Engblom D (2008) Creb has a context-dependent role in activity-regulated transcription and maintains neuronal cholesterol homeostasis. FASEB J 22: 2872-2879.

[8]   Guzowski JF, McNaughton BL, Barnes CA, Worley PF (1999) Environment-specific expression of the immediate-early gene arc in hippocampal neuronal ensembles. Nat Neurosci 2: 1120-1124.

[9]   Kelly MP, Deadwyler SA (2002) Acquisition of a novel behavior induces higher levels of arc mrna than does overtrained performance. Neuroscience 110: 617-626.

[10]   Wang KH, Majewska A, Schummers J, Farley B, Hu C, et al. (2006) In vivo two-photon imaging reveals a role of arc in enhancing orientation specificity in visual cortex. Cell 126: 389-402.

[11]   Chowdhury S, Shepherd JD, Okuno H, Lyford G, Petralia RS, et al. (2006) Arc/arg3.1 interacts with the endocytic machinery to regulate ampa receptor trafficking. Neuron 52: 445-459.

[12]   Rial Verde EM, Lee-Osbourne J, Worley PF, Malinow R, Cline HT (2006) Increased expression of the immediate-early gene arc/arg3.1 reduces ampa receptor-mediated synaptic transmission. Neuron 52: 461-474.

[13]   Messaoudi E, Kanhema T, Soulé J, Tiron A, Dagyte G, et al. (2007) Sustained arc/arg3.1 synthesis controls long-term potentiation consolidation through regulation of local actin polymerization in the dentate gyrus in vivo. J Neurosci 27: 10445-10455.

[14]   Bramham CR, Alme MN, Bittins M, Kuipers SD, Nair RR, et al. (2010) The arc of synaptic memory. Exp Brain Res 200: 125-140.

[15]   Plath N, Ohana O, Dammermann B, Errington ML, Schmitz D, et al. (2006) Arc/arg3.1 is essential for the consolidation of synaptic plasticity and memories. Neuron 52: 437-444.

[16]   Shepherd JD, Rumbaugh G, Wu J, Chowdhury S, Plath N, et al. (2006) Arc/arg3.1 mediates homeostatic synaptic scaling of ampa receptors. Neuron 52: 475-484.

[17]   Kuipers SD, Tiron A, Soule J, Messaoudi E, Trentani A, et al. (2009) Selective survival and maturation of adult-born dentate granule cells expressing the immediate early gene arc/arg3.1. PLoS One 4.

[18]   Qiu Z, Ghosh A (2008) A calcium-dependent switch in a crest-brg1 complex regulates activity-dependent gene expression. Neuron 60: 775-787.

[19]   Flavell SW, Cowan CW, Kim TK, Greer PL, Lin Y, et al. (2006) Activity-dependent regulation of mef2 transcription factors suppresses excitatory synapse number. Science 311: 1008-1012.

[20]   Pintchovski SA, Peebles CL, Kim HJ, Verdin E, Finkbeiner S (2009) The serum response factor and a putative novel transcription factor regulate expression of the immediate-early gene arc/arg3.1 in neurons. J Neurosci 29: 1525-1537.

[21]   Kumar V, Fahey PG, Jong YJ, Ramanan N, O’Malley KL (2011) Activation of the intracellular metabotropic glutamate receptor 5 in striatal neurons leads to upregulation of genes associated with sustained synaptic transmission including arc/arg3.1. J Biol Chem .

[22]   Tian X, Kai L, Hockberger PE, Wokosin DL, Surmeier DJ (2010) Mef-2 regulates activity-dependent spine loss in striatopallidal medium spiny neurons. Mol Cell Neurosci 44: 94-108.

[23]   Kawashima T, Okuno H, Nonaka M, Adachi-Morishima A, Kyo N, et al. (2009) Synaptic activity-responsive element in the arc/arg3.1 promoter essential for synapse-to-nucleus signaling in activated neurons. Proc Natl Acad Sci U S A 106: 316-321.

[24]   Benito E, Valor LM, Jimenez-Minchan M, Huber W, Barco A (2011) camp response element-binding protein is a primary hub of activity-driven neuronal gene expression. J Neurosci 31: 18237-18250.

[25]   Scharfman H, Goodman J, Macleod A, Phani S, Antonelli C, et al. (2005) Increased neurogenesis and the ectopic granule cells after intrahippocampal bdnf infusion in adult rats. Exp Neurol 192: 348-356.

[26]   Pencea V, Bingaman KD, Wiegand SJ, Luskin MB (2001) Infusion of brain-derived neurotrophic factor into the lateral ventricle of the adult rat leads to new neurons in the parenchyma of the striatum, septum, thalamus, and hypothalamus. J Neurosci 21: 6706-6717.

[27]   Danzer SC, Crooks KR, Lo DC, McNamara JO (2002) Increased expression of brain-derived neurotrophic factor induces formation of basal dendrites and axonal branching in dentate granule cells in hippocampal explant cultures. J Neurosci 22: 9754-9763.

[28]   Xu B, Gottschalk W, Chow A, Wilson RI, Schnell E, et al. (2000) The role of brain-derived neurotrophic factor receptors in the mature hippocampus: modulation of long-term potentiation through a presynaptic mechanism involving trkb. J Neurosci 20: 6888-6897.

[29]   Gärtner A, Polnau DG, Staiger V, Sciarretta C, Minichiello L, et al. (2006) Hippocampal long-term potentiation is supported by presynaptic and postsynaptic tyrosine receptor kinase b-mediated phospholipase cgamma signaling. J Neurosci 26: 3496-3504.

[30]   Vaillant AR, Mazzoni I, Tudan C, Boudreau M, Kaplan DR, et al. (1999) Depolarization and neurotrophins converge on the phosphatidylinositol 3-kinase-akt pathway to synergistically regulate neuronal survival. J Cell Biol 146: 955-966.

[31]   Chen MJ, Russo-Neustadt AA (2005) Exercise activates the phosphatidylinositol 3-kinase pathway. Brain Res Mol Brain Res 135: 181-193.

[32]   Niu C, Yip HK (2011) Neuroprotective signaling mechanisms of telomerase are regulated by brain-derived neurotrophic factor in rat spinal cord motor neurons. J Neuropathol Exp Neurol 70: 634-652.

[33]   Lähteinen S, Pitkänen A, Saarelainen T, Nissinen J, Koponen E, et al. (2002) Decreased bdnf signalling in transgenic mice reduces epileptogenesis. Eur J Neurosci 15: 721-734.

[34]   Ramsden M, Berchtold NC, Patrick Kesslak J, Cotman CW, Pike CJ (2003) Exercise increases the vulnerability of rat hippocampal neurons to kainate lesion. Brain Res 971: 239-244.

[35]   Tao X, Finkbeiner S, Arnold DB, Shaywitz AJ, Greenberg ME (1998) Ca2+ influx regulates bdnf transcription by a creb family transcription factor-dependent mechanism. Neuron 20: 709-726.

[36]   Zheng F, Zhou X, Luo Y, Xiao H, Wayman G, et al. (2011) Regulation of brain-derived neurotrophic factor exon iv transcription through calcium responsive elements in cortical neurons. PLoS One 6.

[37]   Takeuchi Y, Miyamoto E, Fukunaga K (2002) Analysis on the promoter region of exon iv brain-derived neurotrophic factor in ng108-15 cells. J Neurochem 83: 67-79.

[38]   Canzoniere D, Farioli-Vecchioli S, Conti F, Ciotti MT, Tata AM, et al. (2004) Dual control of neurogenesis by pc3 through cell cycle inhibition and induction of math1. J Neurosci 24: 3355-3369.

[39]   Farioli-Vecchioli S, Tanori M, Micheli L, Mancuso M, Leonardi L, et al. (2007) Inhibition of medulloblastoma tumorigenesis by the antiproliferative and pro-differentiative gene pc3. FASEB J 21: 2215-2225.

[40]   Passeri D, Marcucci A, Rizzo G, Billi M, Panigada M, et al. (2006) Btg2 enhances retinoic acid-induced differentiation by modulating histone h4 methylation and acetylation. Mol Cell Biol 26: 5023-5032.

[41]   Farioli-Vecchioli S, Saraulli D, Costanzi M, Leonardi L, Cinà I, et al. (2009) Impaired terminal differentiation of hippocampal granule neurons and defective contextual memory in pc3/tis21 knockout mice. PLoS One 4.

[42]   Fass DM, Butler JE, Goodman RH (2003) Deacetylase activity is required for camp activation of a subset of creb target genes. J Biol Chem 278: 43014-43019.

[43]   Sokolova OO, Shtark MB, Lisachev PD, Pustyl’nyak VO, Pan IV (2009) Time course of expression of ”early” genes during long-term posttetanic potentiation in rat hippocampal ca1 field. Bull Exp Biol Med 148: 416-418.

[44]   Bisler S, Schleicher A, Gass P, Stehle JH, Zilles K, et al. (2002) Expression of c-fos, icer, krox-24 and junb in the whisker-to-barrel pathway of rats: time course of induction upon whisker stimulation by tactile exploration of an enriched environment. J Chem Neuroanat 23: 187-198.

[45]   Dragunow M, Faull R (1989) The use of c-fos as a metabolic marker in neuronal pathway tracing. J Neurosci Methods 29: 261-265.

[46]   Kadar E, Aldavert-Vera L, Huguet G, Costa-Miserachs D, Morgado-Bernal I, et al. (2011) Intracranial self-stimulation induces expression of learning and memory-related genes in rat amygdala. Genes Brain Behav 10: 69-77.

[47]   VanElzakker M, Fevurly RD, Breindel T, Spencer RL (2008) Environmental novelty is associated with a selective increase in fos expression in the output elements of the hippocampal formation and the perirhinal cortex. Learn Mem 15: 899-908.

[48]   Fleischmann A, Hvalby O, Jensen V, Strekalova T, Zacher C, et al. (2003) Impaired long-term memory and nr2a-type nmda receptor-dependent synaptic plasticity in mice lacking c-fos in the cns. J Neurosci 23: 9116-9122.

[49]   Gass P, Fleischmann A, Hvalby O, Jensen V, Zacher C, et al. (2004) Mice with a fra-1 knock-in into the c-fos locus show impaired spatial but regular contextual learning and normal ltp. Brain Res Mol Brain Res 130: 16-22.

[50]   Katche C, Bekinschtein P, Slipczuk L, Goldin A, Izquierdo IA, et al. (2010) Delayed wave of c-fos expression in the dorsal hippocampus involved specifically in persistence of long-term memory storage. Proc Natl Acad Sci U S A 107: 349-354.

[51]   Watanabe Y, Johnson RS, Butler LS, Binder DK, Spiegelman BM, et al. (1996) Null mutation of c-fos impairs structural and functional plasticities in the kindling model of epilepsy. J Neurosci 16: 3827-3836.

[52]   Lindecke A, Korte M, Zagrebelsky M, Horejschi V, Elvers M, et al. (2006) Long-term depression activates transcription of immediate early transcription factor genes: involvement of serum response factor/elk-1. Eur J Neurosci 24: 555-563.

[53]   Kalita K, Kharebava G, Zheng JJ, Hetman M (2006) Role of megakaryoblastic acute leukemia-1 in erk1/2-dependent stimulation of serum response factor-driven transcription by bdnf or increased synaptic activity. J Neurosci 26: 10020-10032.

[54]   Lam BY, Zhang W, Enticknap N, Haggis E, Cader MZ, et al. (2009) Inverse regulation of plasticity-related immediate early genes by calcineurin in hippocampal neurons. J Biol Chem 284: 12562-12571.

[55]   Calella AM, Nerlov C, Lopez RG, Sciarretta C, von Bohlen und Halbach O, et al. (2007) Neurotrophin/trk receptor signaling mediates c/ebpalpha, -beta and neurod recruitment to immediate-early gene promoters in neuronal cells and requires c/ebps to induce immediate-early gene transcription. Neural Dev 2: 4-4.

[56]   Tullai JW, Schaffer ME, Mullenbrock S, Kasif S, Cooper GM (2004) Identification of transcription factor binding sites upstream of human genes regulated by the phosphatidylinositol 3-kinase and mek/erk signaling pathways. J Biol Chem 279: 20167-20177.

[57]   Bhattacharya S, Michels CL, Leung MK, Arany ZP, Kung AL, et al. (1999) Functional role of p35srj, a novel p300/cbp binding protein, during transactivation by hif-1. Genes Dev 13: 64-75.

[58]   Bamforth SD, Bragana J, Eloranta JJ, Murdoch JN, Marques FI, et al. (2001) Cardiac malformations, adrenal agenesis, neural crest defects and exencephaly in mice lacking cited2, a new tfap2 co-activator. Nat Genet 29: 469-474.

[59]   Gonzalez YR, Zhang Y, Behzadpoor D, Cregan S, Bamforth S, et al. (2008) Cited2 signals through peroxisome proliferator-activated receptor-gamma to regulate death of cortical neurons after dna damage. J Neurosci 28: 5559-5569.

[60]   Han B, Liu N, Yang X, Sun HB, Yang YC (2001) Mrg1 expression in fibroblasts is regulated by sp1/sp3 and an ets transcription factor. J Biol Chem 276: 7937-7942.

[61]   Borlikova G, Endo S (2009) Inducible camp early repressor (icer) and brain functions. Mol Neurobiol 40: 73-86.

[62]   Mouravlev A, Young D, During MJ (2007) Phosphorylation-dependent degradation of transgenic creb protein initiated by heterodimerization. Brain Res 1130: 31-37.

[63]   Mioduszewska B, Jaworski J, Szklarczyk AW, Klejman A, Kaczmarek L (2008) Inducible camp early repressor (icer)-evoked delayed neuronal death in the organotypic hippocampal culture. J Neurosci Res 86: 61-70.

[64]   Klejman A, Kaczmarek L (2006) Inducible camp early repressor (icer) isoforms and neuronal apoptosis in cortical in vitro culture. Acta Neurobiol Exp (Wars) 66: 267-272.

[65]   Jaworski J, Mioduszewska B, Sánchez-Capelo A, Figiel I, Habas A, et al. (2003) Inducible camp early repressor, an endogenous antagonist of camp responsive element-binding protein, evokes neuronal apoptosis in vitro. J Neurosci 23: 4519-4526.

[66]   Kojima N, Borlikova G, Sakamoto T, Yamada K, Ikeda T, et al. (2008) Inducible camp early repressor acts as a negative regulator for kindling epileptogenesis and long-term fear memory. J Neurosci 28: 6459-6472.

[67]   Porter BE, Lund IV, Varodayan FP, Wallace RW, Blendy JA (2008) The role of transcription factors cyclic-amp responsive element modulator (crem) and inducible cyclic-amp early repressor (icer) in epileptogenesis. Neuroscience 152: 829-836.

[68]   Krueger DA, Mao D, Warner EA, Dowd DR (1999) Functional analysis of the mouse icer (inducible camp early repressor) promoter: evidence for a protein that blocks calcium responsiveness of the cares (camp autoregulatory elements). Mol Endocrinol 13: 1207-1217.

[69]   Brooks-Kayal AR, Raol YH, Russek SJ (2009) Alteration of epileptogenesis genes. Neurotherapeutics 6: 312-318.

[70]   Chung KC, Ahn YS (1998) Expression of immediate early gene cyr61 during the differentiation of immortalized embryonic hippocampal neuronal cells. Neurosci Lett 255: 155-158.

[71]   Kim KH, Min YK, Baik JH, Lau LF, Chaqour B, et al. (2003) Expression of angiogenic factor cyr61 during neuronal cell death via the activation of c-jun n-terminal kinase and serum response factor. J Biol Chem 278: 13847-13854.

[72]   Sin WC, Bechberger JF, Rushlow WJ, Naus CC (2008) Dose-dependent differential upregulation of ccn1/cyr61 and ccn3/nov by the gap junction protein connexin43 in glioma cells. J Cell Biochem 103: 1772-1782.

[73]   Han JS, Macarak E, Rosenbloom J, Chung KC, Chaqour B (2003) Regulation of cyr61/ccn1 gene expression through rhoa gtpase and p38mapk signaling pathways. Eur J Biochem 270: 3408-3421.

[74]   Dobroff AS, Wang H, Melnikova VO, Villares GJ, Zigler M, et al. (2009) Silencing camp-response element-binding protein (creb) identifies cyr61 as a tumor suppressor gene in melanoma. J Biol Chem 284: 26194-26206.

[75]   Hanna M, Liu H, Amir J, Sun Y, Morris SW, et al. (2009) Mechanical regulation of the proangiogenic factor ccn1/cyr61 gene requires the combined activities of mrtf-a and creb-binding protein histone acetyltransferase. J Biol Chem 284: 23125-23136.

[76]   Meyuhas R, Pikarsky E, Tavor E, Klar A, Abramovitch R, et al. (2008) A key role for cyclic amp-responsive element binding protein in hypoxia-mediated activation of the angiogenesis factor ccn1 (cyr61) in tumor cells. Mol Cancer Res 6: 1397-1409.

[77]   Owens DM, Keyse SM (2007) Differential regulation of map kinase signalling by dual-specificity protein phosphatases. Oncogene 26: 3203-3213.

[78]   Davis S, Vanhoutte P, Pages C, Caboche J, Laroche S (2000) The mapk/erk cascade targets both elk-1 and camp response element-binding protein to control long-term potentiation-dependent gene expression in the dentate gyrus in vivo. J Neurosci 20: 4563-4572.

[79]   Jeanneteau F, Deinhardt K, Miyoshi G, Bennett AM, Chao MV (2010) The map kinase phosphatase mkp-1 regulates bdnf-induced axon branching. Nat Neurosci 13: 1373-1379.

[80]   Johansson-Haque K, Palanichamy E, Okret S (2008) Stimulation of mapk-phosphatase 1 gene expression by glucocorticoids occurs through a tethering mechanism involving c/ebp. J Mol Endocrinol 41: 239-249.

[81]   Ryser S, Massiha A, Piuz I, Schlegel W (2004) Stimulated initiation of mitogen-activated protein kinase phosphatase-1 (mkp-1) gene transcription involves the synergistic action of multiple cis-acting elements in the proximal promoter. Biochem J 378: 473-484.

[82]   Lu TC, Wang Z, Feng X, Chuang P, Fang W, et al. (2008) Retinoic acid utilizes creb and usf1 in a transcriptional feed-forward loop in order to stimulate mkp1 expression in human immunodeficiency virus-infected podocytes. Mol Cell Biol 28: 5785-5794.

[83]   Mandl M, Slack DN, Keyse SM (2005) Specific inactivation and nuclear anchoring of extracellular signal-regulated kinase 2 by the inducible dual-specificity protein phosphatase dusp5. Mol Cell Biol 25: 1830-1845.

[84]   Volmat V, Camps M, Arkinstall S, Pouysségur J, Lenormand P (2001) The nucleus, a site for signal termination by sequestration and inactivation of p42/p44 map kinases. J Cell Sci 114: 3433-3443.

[85]   Tárrega C, Nunes-Xavier C, Cejudo-Marín R, Martín-Pérez J, Pulido R (2010) Studying the regulation of map kinase by map kinase phosphatases in vitro and in cell systems. Methods Mol Biol 661: 305-321.

[86]   Domercq M, Alberdi E, Sánchez-Gómez MV, Ariz U, Pérez-Samartín A, et al. (2011) Dual-specific phosphatase-6 (dusp6) and erk mediate ampa receptor-induced oligodendrocyte death. J Biol Chem 286: 11825-11836.

[87]   Jones MW, Errington ML, French PJ, Fine A, Bliss TV, et al. (2001) A requirement for the immediate early gene zif268 in the expression of late ltp and long-term memories. Nat Neurosci 4: 289-296.

[88]   Abraham WC, Dragunow M, Tate WP (1991) The role of immediate early genes in the stabilization of long-term potentiation. Mol Neurobiol 5: 297-314.

[89]   Davis S, Bozon B, Laroche S (2003) How necessary is the activation of the immediate early gene zif268 in synaptic plasticity and learning? Behav Brain Res 142: 17-30.

[90]   Ribeiro S, Mello CV, Velho T, Gardner TJ, Jarvis ED, et al. (2002) Induction of hippocampal long-term potentiation during waking leads to increased extrahippocampal zif-268 expression during ensuing rapid-eye-movement sleep. J Neurosci 22: 10914-10923.

[91]   Zheng D, Butler LS, McNamara JO (1998) Kindling and associated mossy fibre sprouting are not affected in mice deficient of ngfi-a/ngfi-b genes. Neuroscience 83: 251-258.

[92]   Cheval H, Chagneau C, Levasseur G, Veyrac A, Faucon-Biguet N, et al. (2011) Distinctive features of egr transcription factor regulation and dna binding activity in ca1 of the hippocampus in synaptic plasticity and consolidation and reconsolidation of fear memory. Hippocampus .

[93]   Tur G, Georgieva EI, Gagete A, López-Rodas G, Rodríguez JL, et al. (2010) Factor binding and chromatin modification in the promoter of murine egr1 gene upon induction. Cell Mol Life Sci 67: 4065-4077.

[94]   Watson DK, Robinson L, Hodge DR, Kola I, Papas TS, et al. (1997) Fli1 and ews-fli1 function as ternary complex factors and elk1 and sap1a function as ternary and quaternary complex factors on the egr1 promoter serum response elements. Oncogene 14: 213-221.

[95]   Kang JH, Kim MJ, Jang HI, Koh KH, Yum KS, et al. (2007) Proximal cyclic amp response element is essential for exendin-4 induction of rat egr-1 gene. Am J Physiol Endocrinol Metab 292: 215-222.

[96]   Bauer I, Hohl M, Al-Sarraj A, Vinson C, Thiel G (2005) Transcriptional activation of the egr-1 gene mediated by tetradecanoylphorbol acetate and extracellular signal-regulated protein kinase. Arch Biochem Biophys 438: 36-52.

[97]   Russell DL, Doyle KM, Gonzales-Robayna I, Pipaon C, Richards JS (2003) Egr-1 induction in rat granulosa cells by follicle-stimulating hormone and luteinizing hormone: combinatorial regulation by transcription factors cyclic adenosine 3’,5’-monophosphate regulatory element binding protein, serum response factor, sp1, and early growth response factor-1. Mol Endocrinol 17: 520-533.

[98]   Inokuchi K, Murayama A, Ozawa F (1996) mrna differential display reveals krox-20 as a neural plasticity-regulated gene in the rat hippocampus. Biochem Biophys Res Commun 221: 430-436.

[99]   Poirier R, Cheval H, Mailhes C, Charnay P, Davis S, et al. (2007) Paradoxical role of an egr transcription factor family member, egr2/krox20, in learning and memory. Front Behav Neurosci 1: 6-6.

[100]   O’Sullivan NC, Pickering M, Di Giacomo D, Loscher JS, Murphy KJ (2010) Mkl transcription cofactors regulate structural plasticity in hippocampal neurons. Cereb Cortex 20: 1915-1925.

[101]   Swanberg SE, Nagarajan RP, Peddada S, Yasui DH, LaSalle JM (2009) Reciprocal co-regulation of egr2 and mecp2 is disrupted in rett syndrome and autism. Hum Mol Genet 18: 525-534.

[102]   Li L, Yun SH, Keblesh J, Trommer BL, Xiong H, et al. (2007) Egr3, a synaptic activity regulated transcription factor that is essential for learning and memory. Mol Cell Neurosci 35: 76-88.

[103]   Roberts DS, Hu Y, Lund IV, Brooks-Kayal AR, Russek SJ (2006) Brain-derived neurotrophic factor (bdnf)-induced synthesis of early growth response factor 3 (egr3) controls the levels of type a gaba receptor alpha 4 subunits in hippocampal neurons. J Biol Chem 281: 29431-29435.

[104]   Kim JH, Roberts DS, Hu Y, Lau GC, Brooks-Kayal AR, et al. (2012) Brain-derived neurotrophic factor uses creb and egr3 to regulate nmda receptor levels in cortical neurons. J Neurochem 120: 210-219.

[105]   Suehiro J, Hamakubo T, Kodama T, Aird WC, Minami T (2010) Vascular endothelial growth factor activation of endothelial cells is mediated by early growth response-3. Blood 115: 2520-2532.

[106]   Ludwig A, Uvarov P, Soni S, Thomas-Crusells J, Airaksinen MS, et al. (2011) Early growth response 4 mediates bdnf induction of potassium chloride cotransporter 2 transcription. J Neurosci 31: 644-649.

[107]   Uvarov P, Ludwig A, Markkanen M, Rivera C, Airaksinen MS (2006) Upregulation of the neuron-specific k+/cl- cotransporter expression by transcription factor early growth response 4. J Neurosci 26: 13463-13473.

[108]   Ludwig A, Uvarov P, Pellegrino C, Thomas-Crusells J, Schuchmann S, et al. (2011) Neurturin evokes mapk-dependent upregulation of egr4 and kcc2 in developing neurons. Neural Plast 2011: 1-8.

[109]   Ma DK, Jang MH, Guo JU, Kitabatake Y, Chang ML, et al. (2009) Neuronal activity-induced gadd45b promotes epigenetic dna demethylation and adult neurogenesis. Science 323: 1074-1077.

[110]   Zumbrun SD, Hoffman B, Liebermann DA (2009) Distinct mechanisms are utilized to induce stress sensor gadd45b by different stress stimuli. J Cell Biochem 108: 1220-1231.

[111]   Ou DL, Shen YC, Yu SL, Chen KF, Yeh PY, et al. (2010) Induction of dna damage-inducible gene gadd45beta contributes to sorafenib-induced apoptosis in hepatocellular carcinoma cells. Cancer Res 70: 9309-9318.

[112]   Sala C, Futai K, Yamamoto K, Worley PF, Hayashi Y, et al. (2003) Inhibition of dendritic spine morphogenesis and synaptic transmission by activity-inducible protein homer1a. J Neurosci 23: 6327-6337.

[113]   Sala C, Roussignol G, Meldolesi J, Fagni L (2005) Key role of the postsynaptic density scaffold proteins shank and homer in the functional architecture of ca2+ homeostasis at dendritic spines in hippocampal neurons. J Neurosci 25: 4587-4592.

[114]   Lynch G, Rex CS, Gall CM (2007) Ltp consolidation: substrates, explanatory power, and functional significance. Neuropharmacology 52: 12-23.

[115]   Inoue Y, Udo H, Inokuchi K, Sugiyama H (2007) Homer1a regulates the activity-induced remodeling of synaptic structures in cultured hippocampal neurons. Neuroscience 150: 841-852.

[116]   Celikel T, Marx V, Freudenberg F, Zivkovic A, Resnik E, et al. (2007) Select overexpression of homer1a in dorsal hippocampus impairs spatial working memory. Front Neurosci 1: 97-110.

[117]   Martín ED, Sánchez-Perez A, Trejo JL, Martin-Aldana JA, Cano Jaimez M, et al. (2011) Irs-2 deficiency impairs nmda receptor-dependent long-term potentiation. Cereb Cortex .

[118]   Schubert M, Brazil DP, Burks DJ, Kushner JA, Ye J, et al. (2003) Insulin receptor substrate-2 deficiency impairs brain growth and promotes tau phosphorylation. J Neurosci 23: 7084-7092.

[119]   Irvine EE, Drinkwater L, Radwanska K, Al-Qassab H, Smith MA, et al. (2011) Insulin receptor substrate 2 is a negative regulator of memory formation. Learn Mem 18: 375-383.

[120]   Persaud SJ, Liu B, Sampaio HB, Jones PM, Muller DS (2011) Calcium/calmodulin-dependent kinase iv controls glucose-induced irs2 expression in mouse beta cells via activation of camp response element-binding protein. Diabetologia 54: 1109-1120.

[121]   Jhala US, Canettieri G, Screaton RA, Kulkarni RN, Krajewski S, et al. (2003) camp promotes pancreatic beta-cell survival via creb-mediated induction of irs2. Genes Dev 17: 1575-1580.

[122]   Udelhoven M, Pasieka M, Leeser U, Krone W, Schubert M (2010) Neuronal insulin receptor substrate 2 (irs2) expression is regulated by zbp89 and sp1 binding to the irs2 promoter. J Endocrinol 204: 199-208.

[123]   Udelhoven M, Leeser U, Freude S, Hettich MM, Laudes M, et al. (2010) Identification of a region in the human irs2 promoter essential for stress induced transcription depending on sp1, nfi binding and erk activation in hepg2 cells. J Mol Endocrinol 44: 99-113.

[124]   Lerner RG, Depatie C, Rutter GA, Screaton RA, Balthasar N (2009) A role for the creb co-activator crtc2 in the hypothalamic mechanisms linking glucose sensing with gene regulation. EMBO Rep 10: 1175-1181.

[125]   Garrett-Sinha LA, Eberspaecher H, Seldin MF, de Crombrugghe B (1996) A gene for a novel zinc-finger protein expressed in differentiated epithelial cells and transiently in certain mesenchymal cells. J Biol Chem 271: 31384-31390.

[126]   Rowland BD, Peeper DS (2006) Klf4, p21 and context-dependent opposing forces in cancer. Nat Rev Cancer 6: 11-23.

[127]   Rowland BD, Bernards R, Peeper DS (2005) The klf4 tumour suppressor is a transcriptional repressor of p53 that acts as a context-dependent oncogene. Nat Cell Biol 7: 1074-1082.

[128]   Moore DL, Blackmore MG, Hu Y, Kaestner KH, Bixby JL, et al. (2009) Klf family members regulate intrinsic axon regeneration ability. Science 326: 298-301.

[129]   Zhu S, Tai C, MacVicar BA, Jia W, Cynader MS (2009) Glutamatergic stimulation triggers rapid krüpple-like factor 4 expression in neurons and the overexpression of klf4 sensitizes neurons to nmda-induced caspase-3 activity. Brain Res 1250: 49-62.

[130]   Sunadome K, Yamamoto T, Ebisuya M, Kondoh K, Sehara-Fujisawa A, et al. (2011) Erk5 regulates muscle cell fusion through klf transcription factors. Dev Cell 20: 192-205.

[131]   Deaton RA, Gan Q, Owens GK (2009) Sp1-dependent activation of klf4 is required for pdgf-bb-induced phenotypic modulation of smooth muscle. Am J Physiol Heart Circ Physiol 296: 1027-1037.

[132]   Villarreal G, Zhang Y, Larman HB, Gracia-Sancho J, Koo A, et al. (2010) Defining the regulation of klf4 expression and its downstream transcriptional targets in vascular endothelial cells. Biochem Biophys Res Commun 391: 984-989.

[133]   Weston CR, Davis RJ (2007) The jnk signal transduction pathway. Curr Opin Cell Biol 19: 142-149.

[134]   Bode AM, Dong Z (2007) The functional contrariety of jnk. Mol Carcinog 46: 591-598.

[135]   Han D, Zhang QG, Yong-Liu, Li C, Zong YY, et al. (2008) Co-activation of gaba receptors inhibits the jnk3 apoptotic pathway via the disassembly of the glur6-psd95-mlk3 signaling module in cerebral ischemic-reperfusion. FEBS Lett 582: 1298-1306.

[136]   Zhao J, Pei DS, Zhang QG, Zhang GY (2007) Down-regulation cdc42 attenuates neuronal apoptosis through inhibiting mlk3/jnk3 cascade during ischemic reperfusion in rat hippocampus. Cell Signal 19: 831-843.

[137]   Yu C, Minemoto Y, Zhang J, Liu J, Tang F, et al. (2004) Jnk suppresses apoptosis via phosphorylation of the proapoptotic bcl-2 family protein bad. Mol Cell 13: 329-340.

[138]   Ventura JJ, Hübner A, Zhang C, Flavell RA, Shokat KM, et al. (2006) Chemical genetic analysis of the time course of signal transduction by jnk. Mol Cell 21: 701-710.

[139]   Curran BP, Murray HJ, O’Connor JJ (2003) A role for c-jun n-terminal kinase in the inhibition of long-term potentiation by interleukin-1beta and long-term depression in the rat dentate gyrus in vitro. Neuroscience 118: 347-357.

[140]   Liu MG, Wang RR, Chen XF, Zhang FK, Cui XY, et al. (2011) Differential roles of erk, jnk and p38 mapk in pain-related spatial and temporal enhancement of synaptic responses in the hippocampal formation of rats: multi-electrode array recordings. Brain Res 1382: 57-69.

[141]   Zhu Y, Pak D, Qin Y, McCormack SG, Kim MJ, et al. (2005) Rap2-jnk removes synaptic ampa receptors during depotentiation. Neuron 46: 905-916.

[142]   Barnat M, Enslen H, Propst F, Davis RJ, Soares S, et al. (2010) Distinct roles of c-jun n-terminal kinase isoforms in neurite initiation and elongation during axonal regeneration. J Neurosci 30: 7804-7816.

[143]   Oliva AA, Atkins CM, Copenagle L, Banker GA (2006) Activated c-jun n-terminal kinase is required for axon formation. J Neurosci 26: 9462-9470.

[144]   Junghans D, Chauvet S, Buhler E, Dudley K, Sykes T, et al. (2004) The ces-2-related transcription factor e4bp4 is an intrinsic regulator of motoneuron growth and survival. Development 131: 4425-4434.

[145]   MacGillavry HD, Stam FJ, Sassen MM, Kegel L, Hendriks WT, et al. (2009) Nfil3 and camp response element-binding protein form a transcriptional feedforward loop that controls neuronal regeneration-associated gene expression. J Neurosci 29: 15542-15550.

[146]   MacGillavry HD, Cornelis J, van der Kallen LR, Sassen MM, Verhaagen J, et al. (2011) Genome-wide gene expression and promoter binding analysis identifies nfil3 as a repressor of c/ebp target genes in neuronal outgrowth. Mol Cell Neurosci 46: 460-468.

[147]   O’Brien RJ, Xu D, Petralia RS, Steward O, Huganir RL, et al. (1999) Synaptic clustering of ampa receptors by the extracellular immediate-early gene product narp. Neuron 23: 309-323.

[148]   O’Brien R, Xu D, Mi R, Tang X, Hopf C, et al. (2002) Synaptically targeted narp plays an essential role in the aggregation of ampa receptors at excitatory synapses in cultured spinal neurons. J Neurosci 22: 4487-4498.

[149]   Reti IM, Baraban JM (2000) Sustained increase in narp protein expression following repeated electroconvulsive seizure. Neuropsychopharmacology 23: 439-443.

[150]   Chang MC, Park JM, Pelkey KA, Grabenstatter HL, Xu D, et al. (2010) Narp regulates homeostatic scaling of excitatory synapses on parvalbumin-expressing interneurons. Nat Neurosci 13: 1090-1097.

[151]   Hökfelt T, Stanic D, Sanford SD, Gatlin JC, Nilsson I, et al. (2008) Npy and its involvement in axon guidance, neurogenesis, and feeding. Nutrition 24: 860-868.

[152]   Olesen MV, Christiansen SH, Gotzsche CR, Nikitidou L, Kokaia M, et al. (2011) Neuropeptide y y1 receptor hippocampal overexpression via viral vectors is associated with modest anxiolytic-like and proconvulsant effects in mice. J Neurosci Res .

[153]   Brooks PA, Kelly JS, Allen JM, Smith DA, Stone TW (1987) Direct excitatory effects of neuropeptide y (npy) on rat hippocampal neurones in vitro. Brain Res 408: 295-298.

[154]   Reibel S, Nadi S, Benmaamar R, Larmet Y, Carnahan J, et al. (2001) Neuropeptide y and epilepsy: varying effects according to seizure type and receptor activation. Peptides 22: 529-539.

[155]   Decressac M, Wright B, David B, Tyers P, Jaber M, et al. (2011) Exogenous neuropeptide y promotes in vivo hippocampal neurogenesis. Hippocampus 21: 233-238.

[156]   Shalizi A, Gaudillière B, Yuan Z, Stegmüller J, Shirogane T, et al. (2006) A calcium-regulated mef2 sumoylation switch controls postsynaptic differentiation. Science 311: 1012-1017.

[157]   Volakakis N, Kadkhodaei B, Joodmardi E, Wallis K, Panman L, et al. (2010) Nr4a orphan nuclear receptors as mediators of creb-dependent neuroprotection. Proc Natl Acad Sci U S A 107: 12317-12322.

[158]   Lam BY, Zhang W, Ng DC, Maruthappu M, Roderick HL, et al. (2010) Creb-dependent nur77 induction following depolarization in pc12 cells and neurons is modulated by mef2 transcription factors. J Neurochem 112: 1065-1073.

[159]   Winter HY, Marriott SJ (2007) Human t-cell leukemia virus type 1 tax enhances serum response factor dna binding and alters site selection. J Virol 81: 6089-6098.

[160]   Williams GT, Lau LF (1993) Activation of the inducible orphan receptor gene nur77 by serum growth factors: dissociation of immediate-early and delayed-early responses. Mol Cell Biol 13: 6124-6136.

[161]   Latinkic BV, Zeremski M, Lau LF (1996) Elk-1 can recruit srf to form a ternary complex upon the serum response element. Nucleic Acids Res 24: 1345-1351.

[162]   Kim SO, Ono K, Tobias PS, Han J (2003) Orphan nuclear receptor nur77 is involved in caspase-independent macrophage cell death. J Exp Med 197: 1441-1452.

[163]   Peña de Ortiz S, Maldonado-Vlaar CS, Carrasquillo Y (2000) Hippocampal expression of the orphan nuclear receptor gene hzf-3/nurr1 during spatial discrimination learning. Neurobiol Learn Mem 74: 161-178.

[164]   Colón-Cesario WI, Martínez-Montemayor MM, Morales S, Félix J, Cruz J, et al. (2006) Knockdown of nurr1 in the rat hippocampus: implications to spatial discrimination learning and memory. Learn Mem 13: 734-744.

[165]   Crispino M, Tocco G, Feldman JD, Herschman HR, Baudry M (1998) Nurr1 mrna expression in neonatal and adult rat brain following kainic acid-induced seizure activity. Brain Res Mol Brain Res 59: 178-188.

[166]   Zhang T, Wang P, Ren H, Fan J, Wang G (2009) Ngfi-b nuclear orphan receptor nurr1 interacts with p53 and suppresses its transcriptional activity. Mol Cancer Res 7: 1408-1415.

[167]   Castillo SO, Xiao Q, Lyu MS, Kozak CA, Nikodem VM (1997) Organization, sequence, chromosomal localization, and promoter identification of the mouse orphan nuclear receptor nurr1 gene. Genomics 41: 250-257.

[168]   Ji R, Sanchez CM, Chou CL, Chen XB, Woodward DF, et al. (2011) Ep1 prostanoid receptor mediated upregulation of the orphan nuclear receptor nurr1 by camp independent activation of protein kinase a, creb and nf-?b. Br J Pharmacol .

[169]   Darragh J, Soloaga A, Beardmore VA, Wingate AD, Wiggin GR, et al. (2005) Msks are required for the transcription of the nuclear orphan receptors nur77, nurr1 and nor1 downstream of mapk signalling. Biochem J 390: 749-759.

[170]   Lee MK, Nikodem VM (2004) Differential role of erk in camp-induced nurr1 expression in n2a and c6 cells. Neuroreport 15: 99-102.

[171]   España J, Valero J, Miñano-Molina AJ, Masgrau R, Martín E, et al. (2010) beta-amyloid disrupts activity-dependent gene transcription required for memory through the creb coactivator crtc1. J Neurosci 30: 9402-9410.

[172]   Kim Y, Hong S, Noh MR, Kim SY, Huh PW, et al. (2006) Induction of neuron-derived orphan receptor-1 in the dentate gyrus of the hippocampal formation following transient global ischemia in the rat. Mol Cells 22: 8-12.

[173]   Sun W, Choi SH, Park SK, Kim SJ, Noh MR, et al. (2007) Identification and characterization of novel activity-dependent transcription factors in rat cortical neurons. J Neurochem 100: 269-278.

[174]   Pönniö T, Conneely OM (2004) nor-1 regulates hippocampal axon guidance, pyramidal cell survival, and seizure susceptibility. Mol Cell Biol 24: 9070-9078.

[175]   Chen G, Kolbeck R, Barde YA, Bonhoeffer T, Kossel A (1999) Relative contribution of endogenous neurotrophins in hippocampal long-term potentiation. J Neurosci 19: 7983-7990.

[176]   Bramham CR, Southard T, Sarvey JM, Herkenham M, Brady LS (1996) Unilateral ltp triggers bilateral increases in hippocampal neurotrophin and trk receptor mrna expression in behaving rats: evidence for interhemispheric communication. J Comp Neurol 368: 371-382.

[177]   Kamei N, Tanaka N, Oishi Y, Hamasaki T, Nakanishi K, et al. (2007) Bdnf, nt-3, and ngf released from transplanted neural progenitor cells promote corticospinal axon growth in organotypic cocultures. Spine (Phila Pa 1976) 32: 1272-1278.

[178]   Yoo M, Joung I, Han AM, Yoon HH, Kwon YK (2007) Distinct effect of neurotrophins delivered simultaneously by an adenoviral vector on neurite outgrowth of neural precursor cells from different regions of the brain. J Microbiol Biotechnol 17: 2033-2041.

[179]   Holm NR, Christophersen P, Olesen SP, Gammeltoft S (1997) Activation of calcium-dependent potassium channels in mouse [correction of rat] brain neurons by neurotrophin-3 and nerve growth factor. Proc Natl Acad Sci U S A 94: 1002-1006.

[180]   Cheng B, Mattson MP (1994) Nt-3 and bdnf protect cns neurons against metabolic/excitotoxic insults. Brain Res 640: 56-67.

[181]   Xu B, Michalski B, Racine RJ, Fahnestock M (2002) Continuous infusion of neurotrophin-3 triggers sprouting, decreases the levels of trka and trkc, and inhibits epileptogenesis and activity-dependent axonal growth in adult rats. Neuroscience 115: 1295-1308.

[182]   Otal R, Martínez A, Soriano E (2005) Lack of trkb and trkc signaling alters the synaptogenesis and maturation of mossy fiber terminals in the hippocampus. Cell Tissue Res 319: 349-358.

[183]   Ishimaru N, Tabuchi A, Hara D, Hayashi H, Sugimoto T, et al. (2007) Regulation of neurotrophin-3 gene transcription by sp3 and sp4 in neurons. J Neurochem 100: 520-531.

[184]   Yamagata K, Andreasson KI, Sugiura H, Maru E, Dominique M, et al. (1999) Arcadlin is a neural activity-regulated cadherin involved in long term potentiation. J Biol Chem 274: 19473-11979.

[185]   Yasuda S, Tanaka H, Sugiura H, Okamura K, Sakaguchi T, et al. (2007) Activity-induced protocadherin arcadlin regulates dendritic spine number by triggering n-cadherin endocytosis via tao2beta and p38 map kinases. Neuron 56: 456-471.

[186]   Lee KJ, Lee Y, Rozeboom A, Lee JY, Udagawa N, et al. (2011) Requirement for plk2 in orchestrated ras and rap signaling, homeostatic structural plasticity, and memory. Neuron 69: 957-973.

[187]   Seeburg DP, Sheng M (2008) Activity-induced polo-like kinase 2 is required for homeostatic plasticity of hippocampal neurons during epileptiform activity. J Neurosci 28: 6583-6591.

[188]   Yang H, Chen C (2008) Cyclooxygenase-2 in synaptic signaling. Curr Pharm Des 14: 1443-1451.

[189]   Yang H, Zhang J, Andreasson K, Chen C (2008) Cox-2 oxidative metabolism of endocannabinoids augments hippocampal synaptic plasticity. Mol Cell Neurosci 37: 682-695.

[190]   Sang N, Zhang J, Chen C (2007) Cox-2 oxidative metabolite of endocannabinoid 2-ag enhances excitatory glutamatergic synaptic transmission and induces neurotoxicity. J Neurochem 102: 1966-1977.

[191]   Slanina KA, Roberto M, Schweitzer P (2005) Endocannabinoids restrict hippocampal long-term potentiation via cb1. Neuropharmacology 49: 660-668.

[192]   Chen C, Magee JC, Bazan NG (2002) Cyclooxygenase-2 regulates prostaglandin e2 signaling in hippocampal long-term synaptic plasticity. J Neurophysiol 87: 2851-2857.

[193]   Lee J, Kosaras B, Aleyasin H, Han JA, Park DS, et al. (2006) Role of cyclooxygenase-2 induction by transcription factor sp1 and sp3 in neuronal oxidative and dna damage response. FASEB J 20: 2375-2377.

[194]   Chen J, Zhao M, Rao R, Inoue H, Hao CM (2005) C/ebpbeta and its binding element are required for nfkappab-induced cox2 expression following hypertonic stress. J Biol Chem 280: 16354-16359.

[195]   Wang WL, Lee YC, Yang WM, Chang WC, Wang JM (2008) Sumoylation of lap1 is involved in the hdac4-mediated repression of cox-2 transcription. Nucleic Acids Res 36: 6066-6079.

[196]   Xu K, Shu HK (2007) Egfr activation results in enhanced cyclooxygenase-2 expression through p38 mitogen-activated protein kinase-dependent activation of the sp1/sp3 transcription factors in human gliomas. Cancer Res 67: 6121-6129.

[197]   Cortés-Canteli M, Wagner M, Ansorge W, Pérez-Castillo A (2004) Microarray analysis supports a role for ccaat/enhancer-binding protein-beta in brain injury. J Biol Chem 279: 14409-14417.

[198]   Lee B, Dziema H, Lee KH, Choi YS, Obrietan K (2007) Cre-mediated transcription and cox-2 expression in the pilocarpine model of status epilepticus. Neurobiol Dis 25: 80-91.

[199]   Stolle K, Schnoor M, Fuellen G, Spitzer M, Cullen P, et al. (2007) Cloning, genomic organization, and tissue-specific expression of the rasl11b gene. Biochim Biophys Acta 1769: 514-524.

[200]   Oliveira-Dos-Santos AJ, Matsumoto G, Snow BE, Bai D, Houston FP, et al. (2000) Regulation of t cell activation, anxiety, and male aggression by rgs2. Proc Natl Acad Sci U S A 97: 12272-12277.

[201]   Hutchison RM, Chidiac P, Leung LS (2009) Hippocampal long-term potentiation is enhanced in urethane-anesthetized rgs2 knockout mice. Hippocampus 19: 687-691.

[202]   Han J, Mark MD, Li X, Xie M, Waka S, et al. (2006) Rgs2 determines short-term synaptic plasticity in hippocampal neurons by regulating gi/o-mediated inhibition of presynaptic ca2+ channels. Neuron 51: 575-586.

[203]   Seredenina T, Gokce O, Luthi-Carter R (2011) Decreased striatal rgs2 expression is neuroprotective in huntington’s disease (hd) and exemplifies a compensatory aspect of hd-induced gene regulation. PLoS One 6.

[204]   Salim S, Asghar M, Taneja M, Hovatta I, Wu YL, et al. (2011) Novel role of rgs2 in regulation of antioxidant homeostasis in neuronal cells. FEBS Lett 585: 1375-1381.

[205]   Cheng YS, Lee TS, Hsu HC, Kou YR, Wu YL (2008) Characterization of the transcriptional regulation of the regulator of g protein signaling 2 (rgs2) gene during 3t3-l1 preadipocyte differentiation. J Cell Biochem 105: 922-930.

[206]   Xie Z, Liu D, Liu S, Calderon L, Zhao G, et al. (2011) Identification of a camp-response element in the regulator of g-protein signaling-2 (rgs2) promoter as a key cis-regulatory element for rgs2 transcriptional regulation by angiotensin ii in cultured vascular smooth muscles. J Biol Chem 286: 44646-44658.

[207]   Greer PL, Greenberg ME (2008) From synapse to nucleus: calcium-dependent gene transcription in the control of synapse development and function. Neuron 59: 846-860.

[208]   Heidenreich O, Neininger A, Schratt G, Zinck R, Cahill MA, et al. (1999) Mapkap kinase 2 phosphorylates serum response factor in vitro and in vivo. J Biol Chem 274: 14434-14443.

[209]   Johnson AW, Crombag HS, Smith DR, Ramanan N (2011) Effects of serum response factor (srf) deletion on conditioned reinforcement. Behav Brain Res 220: 312-318.

[210]   Alberti S, Krause SM, Kretz O, Philippar U, Lemberger T, et al. (2005) Neuronal migration in the murine rostral migratory stream requires serum response factor. Proc Natl Acad Sci U S A 102: 6148-6153.

[211]   Ramanan N, Shen Y, Sarsfield S, Lemberger T, Schütz G, et al. (2005) Srf mediates activity-induced gene expression and synaptic plasticity but not neuronal viability. Nat Neurosci 8: 759-767.

[212]   Etkin A, Alarcón JM, Weisberg SP, Touzani K, Huang YY, et al. (2006) A role in learning for srf: deletion in the adult forebrain disrupts ltd and the formation of an immediate memory of a novel context. Neuron 50: 127-143.

[213] Zhaolan Zhou et al. (2006) Brain-Specific Phosphorylation of MeCP2 Regulates Activity-Dependent Bdnf Transcription, Dendritic Growth, and Spine Maturation. Neuron, Volume 52, Issue 2, 255-269
